# Supplementary material for: Improving Risk Assessment for Metastatic Disease in Endometrioid Endometrial Cancer Patients Using Molecular and Clinical Features: An NRG Oncology/Gynecologic Oncology Group Study
Source: Cancers (Basel). 2022 Aug 23;14(17):4070. doi: 10.3390/cancers14174070 (PMC9454742; doi:10.3390/cancers14174070)
Supplement: Supplementary file 1 [file cancers-14-04070-s001.zip › cancers-1799702-supplementary-R Supplement.pdf]

Loading the required packages

This provides R codes to identify 23 metastasis-associated transcripts from our TCGA RNAseq training-1 dataset and GOG210 training-2 dataset and provides steps to select a 7-transcript classifier from 23 transcripts using weka software and exhaustive searching method.

Loading the required packages

```
library(stringr);
library(reshape2);
library(gdata);
library(affy);
library(simpleaffy);
library(gplots);
```

Functions

```
# filter the biomarkers with at least threshold_percentage of samples
# having expression values > threshold
# input:


# 1. exp_filename in .csv format, first column is biomarkers,
#    first row is the header, the rest of rows are expression data
# 2. sample_filename in .csv format, has columns for sample and comparison groups,
#    the samples has the same names as in the clinical filename
# 3. col_sample is the sample column in sample_filename
# 4. col_group is the comparison group in sample_filename
# 5. threshold_percentage is filtering criteria, at least threshold_percentage of
#    samples expressed in either one of compared groups
# 6. threshold is filtering criteria, the threshold of biomarker expression values
# 7. output_filename is the filename to save output

    filter_twogroups<-function(exp_filename, sample_filename, col_sample, col_group,


            threshold_percentage, threshold, output_filename) {
            # extract expression data and sample
            data_exp=read.csv(exp_filename, stringsAsFactors=FALSE, check.names=F);
            biomarkers=data_exp[, 1];
```

```
data_exps=data_exp[, -1];
rownames(data_exps)=biomarkers;
data_exp_t=t(data_exps);
samples=rownames(data_exp_t);
data_exps=cbind(samples, data_exp_t);
# extract clinical data
data_sample=read.csv(sample_filename, check.names=F);
data_sample=data_sample[, c(col_sample, col_group)];
data_exps_group=merge(data_sample, data_exps, by.x=1, by.y=1);
data_exps_group=as.matrix(data_exps_group);
sample_groups=data_exps_group[, col_group];
unique_groups=unique(sample_groups);
group1=unique_groups[1];
group2=unique_groups[2];
which_1=which(sample_groups==group1);
which_2=which(sample_groups==group2);
n_1=length(which_1);
n_2=length(which_2);
data_exps_group_biomarkers=apply(data_exps_group[, biomarkers], 2, as.numeric);

# filtering biomarkers
filter_result=apply(data_exps_group_biomarkers, 2, function(x) {

         p1=length(which(x[which_1]>threshold))/length(which_1);
         p2=length(which(x[which_2]>threshold))/length(which_2);
         if (p1>=threshold_percentage | p2>=threshold_percentage){
             return(TRUE);
         } else {

                 return(FALSE);

            }

        });
        kept_biomarkers=biomarkers[filter_result];
        n_samples=length(kept_biomarkers);
        print("number of biomarkers after filtering: ");
        print(n_samples);
        data_kept=data_exp[which(filter_result), ];
        write.csv(data_kept, file=output_filename, row.names=F);
    }

    # Perform univariate logistic regression analysis for each biomarker
    # Input:

        # 1. gene_exp_filename in .csv format has biomarker per row,
        # the first column is biomarker, the rest columns are samples
        # 2. clinical_filename in .csv format has one sample per row,
        # with comparison group information
        # 3. col_sample is the sample column in the clinical filename
        # 4. col_group has values 0, 1, the column for classes in the clinical filename
```

```
    # 5. output_filename will save the univariate logistics regression analysis
    # for each biomarker in .csv format
get_univariate_logisticRegress<- function(gene_exp_filename, clinical_filename,

      col_sample, col_group, output_filename) {
      data0<-read.csv(clinical_filename, check.names=F);
      # extract columns: sample, group from clinical file
      data0<-data0[, c(col_sample, col_group)];
      which_nNA=which(!is.na(data0[, col_group]));
      data0=data0[which_nNA, ];
      colnames(data0)<-c("samples", "Event");
      # extract gene expression data
      data1<-read.csv(gene_exp_filename, stringsAsFactors=FALSE, check.names=F);
      genes<-data1[, 1];
      data2=data1[, -1];
      rownames(data2)=genes;
      data1_t=t(data2);
      samples=rownames(data1_t);
      data1_t=cbind(samples, data1_t);
      data01=merge(data0, data1_t, by.x=1, by.y=1);
      n_which=nrow(data01);
      if (n_which<1) {
          stop("Different sample names in expression file and clinical file");
      }
      data_logistic=data01[, -1];
      data_logistic=apply(data_logistic, 2, as.numeric);
      results<-c();
      genes=colnames(data_logistic);
      for (i in 2:ncol(data_logistic)){

      # for each gene, perform univariate logistic regression
      geneID<-genes[i];
      oneGeneData<-data_logistic[, c("Event", geneID)];
      oneGeneData<-data.frame(oneGeneData);
      colnames(oneGeneData)<-c("Event", "gene");
      mylogit <- glm(Event ~ gene, data = oneGeneData, family = "binomial");
      summ<-summary(mylogit);
      confs<-summ$coefficients;
      oneResult<-c(geneID, confs[2, c(1,2,4)]);
      results<-rbind(results, oneResult);

       }
       colnames(results)<-c("biomarker", "coefficient", "std. Error", "Pvalue");
       write.csv(results, file=output_filename, quote=FALSE, row.names=F);

    }

    # Perform randomly subsampling samples


        # Input:
        # 1. clinical_filename in .csv format has one sample per row,
```

```
#    with comparison group information
# 2. col_sample is the sample column in the clinical filename
# 3. col_group has values 0, 1, the column for classes in the clinical filename
# 4. percenage_selected is percentage of samples selected
# 5. n_times is the selecting times
# 6. output_filename will selected samples in .csv format,
# each column is one selected samples

sampling_trainingDataset<-function(clinical_filename, col_sample, col_group,

  percenage_selected, n_times, output_filename){
  data0<-read.csv(clinical_filename, check.names=F);
  data0=as.matrix(data0);
  clinical_filename=basename(clinical_filename);
  filename_infos=strsplit(clinical_filename, split=".csv");
  filenameHead=unlist(filename_infos)[1];
  # extract columns: sample and stages
  samples=data0[, col_sample];
  sample_groups<-data0[, col_group];
  unique_groups=unique(sample_groups);
  group1=unique_groups[1];
  group2=unique_groups[2];
  which_1=which(sample_groups==group1);
  which_2=which(sample_groups==group2);
  samples_1=samples[which_1];
  samples_2=samples[which_2];
  n_1=length(which_1);
  n_2=length(which_2);
  # get 80% samples
  n1=round(percenage_selected*n_1);
  n2=round(percenage_selected*n_2);
  randomly_samples=c();
  for (i in 1:n_times){

    sample1=sample(samples_1, n1, replace=F);
    sample2=sample(samples_2, n2, replace=F);
    selected_samples<-c(sample1, sample2);
    randomly_samples<-cbind(randomly_samples, selected_samples);

   }
   write.csv(randomly_samples, file=output_filename, quote=FALSE, row.names=F);
}

# Perform univariate logistic regression analysis for randomly subsampling many times
# Input:

# 1. gene_exp_filename in .csv format has gene per row, the first column is
#    gene name/ID, the rest columns are samples
# 2. random_selected_filename is the selected samples, each # column is one random selection
# 3. clinical_filename in .csv format has one sample per row, with group information
# 4. col_sample is the sample column in the clinical filename
```

```
# 5. col_group has values 0, 1, the column for classes in the clinical filename
# 6. n_times is the selecting times
# 7. output_directory will save the univariate logistics regression analysis
# for each selection


runs_univariate_logisticRegress<- function(gene_exp_filename, random_selected_filename,

    clinical_filename, col_sample, col_group, n_times, output_directory) {
    data_clinical<-read.csv(clinical_filename, check.names=F);
    clinical_samples<-as.character(data_clinical[, col_sample]);
    data_selection=read.csv(random_selected_filename, check.names=F);
    for (i in 1:n_times){

         sample_selected=as.character(data_selection[, i]);
         which_in=which(clinical_samples %in% sample_selected);
         selected_clinical=data_clinical[which_in, ];
         write.csv(selected_clinical, file="tmp.csv", quote=FALSE, row.names=F);
         output_filename=paste(output_directory, "/", "univariate_logistcRegresssion_",
         i, ".csv", sep="");
         get_univariate_logisticRegress(gene_exp_filename, "tmp.csv", col_sample,
         col_group, output_filename);

         }

     }


    # get the significant genes with P-value> threshold
    # input:

# 1. Result_filename is the filename for comparison results with P-values
# 2. col_Pvalue is the column for P-value
# 3. threshold is threhold for P-value, default is 0.05
# 4. sign_filename is the file to save the significant biomarkers with P-value>threshold

     get_significant_biomarkers<-function(Result_filename, col_Pvalue,

         threshold=0.05, sign_filename){
         data_Result=read.csv(Result_filename, check.names=F);
         Pvalues=as.numeric(as.character(data_Result[, col_Pvalue]));
         which_sign=which(Pvalues<threshold);
         data_sign=data_Result[which_sign, ];
         print("the number of significant genes is: ");
         print(nrow(data_sign));
         write.csv(data_sign, file=sign_filename, quote=FALSE, row.names=F);

     }
     redblackgreen <- colorRampPalette(c("green", "black", "red"))(n = 100);
     distFunctions<-function(x){

         method0="pearson";
         corrs=cor(t(x), method=method0, use="pairwise.complete.obs");
         corrs[which(is.na(corrs))]=0;
         corr_dist=as.dist(1-corrs);
         return(corr_dist);

     }
```

Begin to run

```
# need change the directory for your data
dir<-"C:/Users/wangg/projects_2014_0211/Maxwell/prepare_codes_data_submission";
setwd(dir);

# step 1: extract log2-transformed gene expression data for Training-1 samples
print("generating training 1 ...");
```

```
## [1] "generating training 1 ..."
```

```
file_exp="input_data/Training1_RNAseqV2_normalized.csv";
file_clinical="input_data/Training1_clinical.csv";
data_exp=read.csv(file_exp, check.names=F);
data_clinical=read.csv(file_clinical, check.names=F);
samples=as.character(data_clinical[, "samples"]);
data_exp=data_exp[, c("genes", samples)];
print("The number of genes in Training-1:");
```

```
## [1] "The number of genes in Training-1:"
```

```
print(nrow(data_exp));
```

```
## [1] 20531
```

```
output_directory="generated_data";
if (!dir.exists(output_directory)){
dir.create(output_directory)
} else {

      print("Dir already exists!")
 }
```

```
outfile="generated_data/Training1_RNAseqV2_normalized.csv";
write.csv(data_exp, file=outfile, row.names=F);

# step 2: filter biomarkers for Training-1
print("filtering genes in training-1...");
```

```
## [1] "filtering genes in training-1..."
```

```
exp_filename="generated_data/Training1_RNAseqV2_normalized.csv";
sample_filename="input_data/Training1_clinical.csv";
col_sample="samples";
group_col="stages";
threshold_percentage=2.0/3.0;
threshold=0;

output_filename="generated_data/Training1_RNAseqV2_normalized_filter2thirds.csv";
filter_twogroups(exp_filename,sample_filename,col_sample,group_col,
threshold_percentage,threshold,output_filename);
```

```
## [1] "number of biomarkers after filtering: "
## [1] 17265
```

```
# step 3: perform univariate logistic regression in all of samples in TCGA training dataset
print("Performing univariate logistic regression modeling of metastasis in Training-1...");
```

```
## [1] "Performing univariate logistic regression modeling of metastasis in Training-1..."
```

```
gene_exp_filename="generated_data/Training1_RNAseqV2_normalized_filter2thirds.csv";
clinical_filename="input_data/Training1_clinical.csv";
col_sample="samples";
col_group="Event";
output_filename=
"generated_data/logistic_univariate_Training1_RNAseqV2_normalized_filter2thirds.csv";
get_univariate_logisticRegress(gene_exp_filename, clinical_filename, col_sample,
col_group, output_filename);

# step 4: getting the significant genes for training-1
print("getting the significant genes for training-1...");
```

```
## [1] "getting the significant genes for training-1..."
```

```
Result_filename=
"generated_data/logistic_univariate_Training1_RNAseqV2_normalized_filter2thirds.csv";
col_Pvalue="Pvalue";
threshold=0.05;
sign_filename="generated_data/sign_logistic_univariate_Training1.csv";
get_significant_biomarkers(Result_filename, col_Pvalue, threshold=0.05, sign_filename);
```

```
## [1] "the number of significant genes is: "
## [1] 1630
```

```
# step 5: getting the expression data for significant genes in Training-1,
# generating supplemental table 1
print("getting the expression data for significant genes in Training-1...");
```

```
## [1] "getting the expression data for significant genes in Training-1..."
```

```
gene_exp_filename="generated_data/Training1_RNAseqV2_normalized_filter2thirds.csv";
sign_filename="generated_data/sign_logistic_univariate_Training1.csv";
gene_sign_filename="generated_data/Training1_signGenes.csv";
data_exp=read.csv(gene_exp_filename, check.names=F);
data_sign=read.csv(sign_filename, check.names=F);
genes=as.character(data_exp[, 1]);
genes_inSign=as.character(data_sign[, 1]);
which_sign=which(genes %in% genes_inSign);
data_sign_exp=data_exp[which_sign, ];
write.csv(data_sign_exp, file=gene_sign_filename, row.names=F, quote=FALSE);

# step 6: sampling 80% samples in Training-1
```

```
print("generating 100 subsamples of Training-1...");
```

```
## [1] "generating 100 subsamples of Training-1..."
```

```
clinical_filename="input_data/Training1_clinical.csv";
col_group="Event";
col_sample="samples";
percenage_selected=0.8;
n_times=100;
output_filename="generated_data/randomly_pick_100times_Training_onetime.csv";
sampling_trainingDataset(clinical_filename,col_sample,col_group,
percenage_selected,n_times,output_filename);

# step 7: run 100 times logisitic regression modeling for subsamples in
# Training-1 for significant genes in step4
print("perfroming univariate logistic regression modeling in 100 subsampling...");
```

```
## [1] "perfroming univariate logistic regression modeling in 100 subsampling..."
```

```
gene_exp_filename="generated_data/Training1_signGenes.csv";
clinical_filename="input_data/Training1_clinical.csv";
# the random selection file is from the selection used in the paper in order to
# duplicate the paper results, not from the generated one by this run
random_selected_filename="input_data/randomly_pick_100times_Training1.csv";

col_sample="samples";
col_group="Event";
n_times=100;
output_directory="generated_data/logistic_Training1_100times";
if (!dir.exists(output_directory)){
dir.create(output_directory)
} else {

        print("Dir already exists!")

  }

      runs_univariate_logisticRegress(gene_exp_filename, random_selected_filename,
      clinical_filename, col_sample, col_group, n_times, output_directory);

      # step 8: get the significant genes after 100 times subsampling,
      # and generating supplemental table 2
      Results_100times=c();
      for (i in 1:100){

       head="generated_data/logistic_Training1_100times/univariate_logistcRegresssion_";
       file_oneSampling=paste(head, i, ".csv", sep="");
       OneResults=read.csv(file_oneSampling, check.names=F);
       oneResult=OneResults[, c("biomarker", "Pvalue")];
       colnames(oneResult)[2]=paste(colnames(oneResult)[2], "_run", i, sep="");
       if (i==1){

            Results_100times=oneResult;
```

```
      } else {

                Results_100times=merge(Results_100times, oneResult, by.x=1, by.y=1);

           }

        }

Results_data=apply(Results_100times[, -1], 2, as.numeric);
rownames(Results_data)=as.character(Results_100times[, 1]);
number_P005=apply(Results_data, 1, function(x) length(which(x<0.05)));
maxPvalue=apply(Results_data, 1, function(x) max(x[which(x<0.05)]));
genes=rownames(Results_data);
finalResult=cbind(genes, number_P005, maxPvalue);
which_p=which(number_P005>=80);
n_selected=length(which_p);
finalResults=finalResult[which_p, ];
colnames(finalResults)=c("GeneSymbol|ID",
    "Percent of the 100 Training-1 Models with 60 of the 75 cases with p<0.05",
    "Max P-value in the Training-1 Models that passed with p-value <0.05");
outfile="generated_data/selection_from_Training1.csv";
write.csv(finalResults, file=outfile, row.names=F, quote=FALSE);
print("the number of genes associated with metastasis with p<0.05 in at least 80 of the 100:

## [1] "the number of genes associated with metastasis with p<0.05 in at least 80 of the 100:

print(n_selected);

## [1] 311

# step 9: mapping 311 genes with probesets in Affymetrix platform where probesets
# mapping to more than one gene were removed
# download gene symbol with aliases from
# ftp://ftp.ncbi.nlm.nih.gov/gene/DATA/GENE_INFO/Mammalia/Homo_sapiens.gene_info.gz
# run internal perl script to get the mapped genes
cmd1="perl get_probesets_mapped_to_oneGene.pl";
cmd2="perl mapping_311genes_to_Affymetrix.pl";
system(cmd1);
system(cmd2);

# step 10: getting normalized expression data from Training-2
raw.data <- read.affy(path="input_data/Training2", covdesc="covdesc_Training2");
x.rma <- call.exprs(raw.data,"rma");

## Background correcting
## Normalizing
## Calculating Expression

expData<-exprs(x.rma);
colnames(expData)=gsub(".CEL", "", colnames(expData));
probesets=rownames(expData);
expData=cbind(probesets, expData);
n_probesets=nrow(expData);
```

```
print(n_probesets);

## [1] 54675

outfile="generated_data/Training2_Affy_64samples_processed.csv";
write.csv(expData, file=outfile, quote=FALSE, row.names=FALSE);

# step 11: getting the expression data for significant genes in Training-2
print("getting the expression data for significant genes in Training-2...");

## [1] "getting the expression data for significant genes in Training-2..."

gene_exp_filename="generated_data/Training2_Affy_64samples_processed.csv";
sign_filename="generated_data/selection_from_Training1_with_Affyprobesets.csv";
gene_sign_filename="generated_data/Training2_Affy_64samples_processed_signGenes.csv";
data_exp=read.csv(gene_exp_filename, check.names=F);
data_sign=read.csv(sign_filename, check.names=F);
probesets=as.character(data_exp[, 1]);
probesets_inSign=as.character(data_sign[, 1]);
which_sign=which(probesets %in% probesets_inSign);
data_sign_exp=data_exp[which_sign, ];
write.csv(data_sign_exp, file=gene_sign_filename, row.names=F, quote=FALSE);

# step 12: getting the significant genes for training-2
print("getting the significant genes for training-2 and saving to the file...");

## [1] "getting the significant genes for training-2 and saving to the file..."

gene_exp_filename="generated_data/Training2_Affy_64samples_processed_signGenes.csv";
clinical_filename="input_data/Training2_clinical.csv";
col_sample="Samples";
col_group="Matastasis_status";
output_filename=
"generated_data/logistic_univariate_Training2_Affy_64samples_processed_filteredBytraing1.csv";

get_univariate_logisticRegress(gene_exp_filename, clinical_filename,
col_sample, col_group, output_filename);


# step 13: merge with gene names, and generating supplement table 4
file1="generated_data/logistic_univariate_Training2_Affy_64samples_processed_filteredBytraing1.
data1=read.csv(file1, check.names=F);
Pvalues=as.numeric(as.character(data1[, "Pvalue"]));
which_P=which(Pvalues<0.05);
data1=data1[which_P, ];
colnames(data1)=paste(colnames(data1), "_T2", sep="");
file2="generated_data/selection_from_Training1_with_Affyprobesets.csv";
data2=read.csv(file2, check.names=F);
data2=data2[, c(1, 4)];
```

```
data12=merge(data2, data1, by.x=1, by.y=1);
file3="generated_data/sign_logistic_univariate_Training1.csv";
data3=read.csv(file3, check.names=F);
colnames(data3)=paste(colnames(data3), "_T1", sep="");
data123=merge(data3, data12, by.x=1, by.y=2);
data123_coef=data123[, c("coefficient_T1", "coefficient_T2")];
data123_coef=apply(data123_coef, 2, as.numeric);
coefs=data123_coef[, 1] * data123_coef[, 2];
#same direction

which_coef=which(coefs>0);
data123_sameDirection=data123[which_coef, ];
#pick the probesets with the lowest p-values for one gene in GOG210 cohort
genes=as.character(data123_sameDirection[, 1]);
uniqueGenes=unique(genes);
print("number of unique genes: ");
```

```
## [1] "number of unique genes: "
```

```
print(length(uniqueGenes));
```

```
## [1] 23
```

```
picked_results=c();
for (i in 1:length(uniqueGenes)){

       oneGeneData=data123_sameDirection[which(genes==uniqueGenes[i]), ];
       pvalues_oneGene=as.numeric(as.character(oneGeneData[, "Pvalue_T2"]));
       which_p=which(pvalues_oneGene==min(pvalues_oneGene));
       pickedOne=oneGeneData[which_p, ];
       picked_results=rbind(picked_results, pickedOne);

  }
  picked_result_show=picked_results[, c(1,2,4,5,6,8)];
  print(picked_result_show);
```

```
##      biomarker_T1 coefficient_T1 Pvalue_T1    probeset coefficient_T2   Pvalue_T2
## 1     APOL4|80832     -0.4659351     0.010101509 223801_s_at     -1.0615131     0.023077196
## 2    BDNFOS|497258    -0.7367480     0.008188074   239367_at     -0.7261614     0.045352239
## 3    C8orf79|57604    -0.3874238     0.008436128   239297_at     -0.3798567     0.046766093
## 6    COL18A1|80781    -0.8458287     0.007235258 209081_s_at     -0.9994259     0.011980288
## 8     GTPBP4|23560     1.4270714     0.009477272 218239_s_at      1.2855675     0.046618402
## 10     HOXD13|3239     0.3994952     0.009443663   207398_at      2.9014850     0.032552500
## 13       LDLR|3949     0.7151780     0.008430980 214170_x_at      1.8770445     0.004488865
## 16     LUC7L|55692    -1.2856259     0.001289186  1557066_at     -1.0567871     0.012328832
## 18     MLLT10|8028     1.2737948     0.005820146 216503_s_at      1.3315629     0.029502764
## 19    MRPL37|51253     1.5958576     0.007059514   218887_at      2.5091005     0.008911708
## 21    PDLIM3|27295     0.3837614     0.008955284 209621_s_at      1.8739511     0.008952823
## 22  PTPLAD1|51495     1.2719306     0.001124766 217777_s_at      0.7734904     0.030842697
## 25     RSRC1|51319     1.0763783     0.002060703 235354_s_at      0.8458092     0.023470188
```

```
## 26   SCAPER|49855      0.9406323     0.014259344   215848_at      0.9414190     0.046740568
## 27       SCD|6319      0.6976762     0.010313498 211708_s_at      1.3483646     0.001991616
## 31   SFRS2B|10929     -1.2781185     0.011357525   238929_at     -1.2498443     0.038142975
## 32 SLC35E2|728661     -1.7685339     0.008424676 217122_s_at     -1.3065196     0.014443606
## 34    TBRG1|84897     -1.3821316     0.005882552   230681_at     -0.9620054     0.039867462
## 35      TFRC|7037      0.6984345     0.004610531   237214_at      4.2190367     0.013479149
## 37    ZFP36L2|678     -1.0859963     0.002245582   201368_at     -0.5780422     0.044735737
## 38     ZNF10|7556     -0.9331635     0.007093707   229848_at     -1.0693432     0.018374335
## 40    ZNF140|7699     -1.2853229     0.007181836   204523_at     -1.6021212     0.029201949
## 41  ZNF596|169270     -0.9559010     0.009001495   232641_at     -1.1236305     0.007800549
```

```
outfile="generated_data/logistic_univariate_T1_T2_signficantwithsameDirection.csv";
write.csv(picked_results, file=outfile, row.names=F);
```

Selecting an optimal combination from 23 genes using exhausted search

1. Get all possible combination of genes There are 8,388,607 possible gene combinations for selected 23 genes.
2. For each combination, get the normalized gene expressed data with stage information in the last column
3. Run weka by following command for each normalized gene expressed data file: java -cp weka.jar weka.classifiers.functions.Logistic -t input_filename -x 10 > out_filename
4. Merge the performance result for each combination and select the optimal combination by our criteria
5. For last selected gene list, using Lasso to determine if they are final selection.

Notes:

1. Subsampling 100 times may generate slightly different subsamples each time, The selection used in the publication was saved and used here to generate the same results as in the publication. subsampling 1000 times or more may generate more consistant result.
2. The downloaded human gene_info from NCBI and affymetrix probeset annotation may be slightly different in each downloaded time.
