# Supplementary material for: Improving Risk Assessment for Metastatic Disease in Endometrioid Endometrial Cancer Patients Using Molecular and Clinical Features: An NRG Oncology/Gynecologic Oncology Group Study
Source: Cancers (Basel). 2022 Aug 23;14(17):4070. doi: 10.3390/cancers14174070 (PMC9454742; doi:10.3390/cancers14174070)
Supplement: Supplementary file 1 [file cancers-14-04070-s001.zip › cancers-1799702-supplementary.pdf]

Supplement S1 to S15

Table of Contents

Section	Title	Pages
S1	**Transcript Selection in Training-1**	**2-30**
S2	**Limit Transcript Selection with Random Re-Sampling in Training-1**	**31-36**
S3	**Evaluate Candidate Transcripts in Training-2**	**37**
S4	**Require Consistency in Candidate Transcripts in Training Cohorts**	**38**
S5	**Nodal and Distant Metastasis Prediction with Clinical Factors and/or Molecular Features in Training-1**	**39-41**
S6	**Odds of Nodal and Distant Metastasis based on the MS7 Classifier, Grade 3 and/or Myometrial Invasion**	**42**
S7	**Raw Data for Evaluating Risk of Metastasis in Validation-1 and Validation-2**	**43-51**
S8	**MS7 Classifier Correlations with Cancer Biomarker Expression**	**52**
S9	**MS7 Classifier Relationship with Mutations in Cancer Biomarkers**	**53**
S10	**Evaluations for the MS7 Classifier, Molecular Subtypes and Other Molecular Features**	**54-57**
S11	**Differential Gene Expression in Training-1 Patients with Extreme MS7 Scores**	**58-62**
S12	**Enriched Pathways in Training-1 Patients with Extreme MS7 Scores**	**63-64**
S13	**Pathway Analyses of Differentially Expressed Transcripts in Endometrioid Endometrial Cancer Patients Classified with High versus Low Risk of Metastasis.**	**65-67**
S14	**Stage Distribution of RNA Sequencing-Based MS7 Score**	**68**
S15	**Additional Characteristics of the Cohorts and Exploratory Evaluations of the Potential Prognostic Relevance of the MS7 Classifier**	**69-71**

Supplement S1 to S15

S1. Transcript-Selection in Training-1.

Table S1 provides a summary of the 1,630 transcripts (Gene Symbol |ID) that distinguished nodal (stage IIIC) or distant (stage IV) endometrioid endometrial cancer (EEC) from stage I EEC. The relationship with metastasis was assessed using univariate logistic regression modeling with individual transcripts included as the explanatory variable. Statistical significance was set at $p<0.05$. These loose criteria did not include correction for false-discovery. The coefficient, standard error and p-value for the individual transcripts in the univariate logistic regression model in the 75 cases in Training-1 were calculated using R and ranked in ascending order by p-value. The seven transcripts in the final MS7 classifier are bolded and highlighted in blue.

Table S1.

	Univariate Logistic Modeling in Training-1		
**GeneSymbol | ID**	**Coefficient**	**Standard Error**	**P-value**
FAM170A|340069	-0.982421987	0.256926531	0.000131439
PCDHGA1|56114	0.793237589	0.216812449	0.000253562
SENP3|26168	1.968204311	0.592258005	0.000889843
MAP2K1|5604	1.940295914	0.590608098	0.001018915
PTPLAD1|51495	1.271930639	0.390485902	0.001124766
WDR64|128025	-1.102892563	0.339107498	0.001144594
C6orf164|63914	-0.902868075	0.277984441	0.001162566
NLRP9|338321	-0.781259641	0.240830584	0.001178548
CDRT4|284040	-1.278540999	0.397193576	0.001286669
FGFR2|2263	-0.588564779	0.182869881	0.001288677
LUC7L|55692	-1.285625942	0.399464169	0.001289186
C14orf93|60686	-2.264781954	0.706795149	0.001353925
TXLNG|55787	1.398319103	0.437768485	0.0014022
ETF1|2107	2.189811489	0.688662405	0.001473747
CCDC130|81576	-1.349244648	0.425217714	0.001508373
ITIH3|3699	-0.861787013	0.271958704	0.001530614
FAM116B|414918	-0.890097745	0.281286077	0.001554105
TMEM22|80723	0.847209592	0.26776304	0.001556022
C12orf60|144608	-1.280096713	0.407682634	0.001689857
CDC16|8881	-2.38335072	0.762983958	0.001785783
ZNF688|146542	-1.171927148	0.37664338	0.001861373
HSCB|150274	-1.44194983	0.464713846	0.001916494
**RSRC1|51319**	**1.076378266**	**0.349321336**	**0.002060703**
CROCC|9696	-1.175205311	0.381538368	0.002068783
ZNF528|84436	-0.864922982	0.282358167	0.002189747
C22orf43|51233	-0.66377434	0.216946365	0.002216149
EIF2S2|8894	2.4444906	0.799169617	0.002222342
ZFP36L2|678	-1.085996313	0.355403649	0.002245582
KIAA0196|9897	1.378963942	0.452171369	0.00229109
WFDC8|90199	-0.518410872	0.170159736	0.002314354
PCDHAC2|56134	0.435401841	0.143140361	0.002351818
IFT27|11020	-1.038539502	0.342170687	0.002404158
MED14|9282	1.58992677	0.526277277	0.002518732

TTC21A|199223	-0.61150961	0.202621488	0.002544574
TRIM50|135892	-0.727374357	0.241131334	0.002557053
PCDHGA3|56112	0.50133265	0.166319307	0.002575915
RGP1|9827	0.92836827	0.30818057	0.002591766
KRTAP5-10|387273	-0.575230285	0.191016959	0.002600379
TRPM4|54795	-0.801324535	0.266443867	0.002634237
CUBN|8029	-0.670190963	0.223496504	0.00271166
C12orf27|283460	-0.497602388	0.16631263	0.002771836
C6orf70|55780	-1.343978471	0.449406227	0.00278462
LOC143188|143188	-1.051539976	0.353047656	0.002896964
H2AFB1|474382	-0.690404065	0.231818363	0.00289936
STX19|415117	-0.491387137	0.165221824	0.00293844
LRP5L|91355	-0.883576558	0.297096135	0.002939043
LTB4R2|56413	-1.194213211	0.40236277	0.002997431
FAM53A|152877	-0.783599818	0.264278174	0.00302627
ETS2|2114	-1.013874261	0.341943037	0.003026451
DDX24|57062	2.045308488	0.690166494	0.003041619
LRRC27|80313	-1.075726979	0.363701493	0.003099241
COL28A1|340267	-0.494862157	0.168241873	0.003267605
CPT1B|1375	-0.742175709	0.252806702	0.00332749
LOC728743|728743	-0.855069603	0.291736702	0.003379126
CYP2E1|1571	-0.463931683	0.158319084	0.003385711
EPR1|8475	0.371561531	0.126969347	0.003429233
GLTSCR2|29997	-1.189119883	0.406577155	0.003447784
CALB1|793	0.326243716	0.112325309	0.00367894
ACAD11|84129	-1.093019317	0.376551714	0.003699525
C2orf81|388963	-0.797873789	0.274949647	0.003709181
YWHAB|7529	1.982233869	0.683123017	0.003711164
QRICH1|54870	2.071723288	0.714366268	0.003730608
SPINLW1|57119	-0.349450322	0.120671767	0.003781033
PSMD8|5714	2.015838567	0.696658302	0.003808737
NAA50|80218	1.353336806	0.468130756	0.003840944
C1orf228|339541	-0.636870844	0.220441134	0.003863776
WASH3P|374666	-1.093429572	0.378579412	0.003873997
TNIP2|79155	-1.653868558	0.572660759	0.003876476
OR2A9P|441295	-0.855813307	0.297000837	0.003957644
PAFAH1B1|5048	2.153832642	0.747795199	0.003973675
GSK3B|2932	1.637249367	0.568730007	0.003992149
CCDC88A|55704	0.611716339	0.212493479	0.003992503
CCDC57|284001	-1.034090182	0.359828509	0.004055121
UROS|7390	-1.508525093	0.525746173	0.004113774
GFM1|85476	1.371378736	0.478236649	0.004136327
SKINTL|391037	-0.60081653	0.20972749	0.004173405
C19orf18|147685	-0.502137642	0.175393039	0.004197459
LRRC42|115353	1.199760176	0.419801654	0.004264259
C2CD2|25966	-1.403602701	0.491791836	0.004316457
MRPL49|740	1.652329193	0.579212612	0.004334732
MRPL13|28998	1.514230569	0.531496211	0.004385743
C1orf101|257044	-0.684851334	0.241637366	0.004593896
TFRC|7037	0.698434467	0.246530417	0.004610531
DLST|1743	1.64423539	0.580976275	0.004652984
RCE1|9986	1.975754163	0.700622986	0.004802422
LIMS3-LOC440895|100271835	-0.376410775	0.133662541	0.004860635
PSMA7|5688	1.589054407	0.564992719	0.004915467
TUBB1|81027	1.063101405	0.378246052	0.004944798
C1orf152|767846	-0.664736112	0.236810482	0.004999928
PRKDC|5591	0.789129557	0.281167047	0.005006399
XPO5|57510	1.509810148	0.538056348	0.005015374
SF3B2|10992	2.255184461	0.804148917	0.005040464

TMED6|146456	-0.373200503	0.133132484	0.005059413
IMPA2|3613	1.004867027	0.358946236	0.005118312
PCOLCE2|26577	0.325941037	0.116478265	0.00513725
HCG2P7|80867	-0.546707137	0.195383884	0.005140135
ATP5B|506	1.199247511	0.429046266	0.005187607
TBC1D3C|414060	-0.671268073	0.240233863	0.005202386
ARFGEF1|10565	1.350108207	0.483408831	0.005223928
F2R|2149	0.891570816	0.319935012	0.005324368
LDLRAD2|401944	-0.543043223	0.195003613	0.005356315
C4orf44|345222	-0.769100355	0.27626619	0.005370785
SMURF1|57154	1.711172299	0.616907003	0.005540693
PRPS1|5631	1.32913695	0.480010338	0.005623283
CCDC94|55702	-1.266959761	0.458019722	0.005671921
FAM193A|8603	-1.889907301	0.683323078	0.00567904
RP1L1|94137	-0.439834736	0.159194702	0.005729506
SUGT1L1|283507	-0.672547644	0.24348655	0.005742117
IL20|50604	-0.577127616	0.20895709	0.005745847
GIYD2|79008	-0.906487428	0.328394804	0.005773794
**MLLT10|8028**	**1.2737948**	**0.461897122**	**0.005820146**
C19orf25|148223	-1.211931885	0.439583974	0.005833481
KLK2|3817	-0.450047779	0.163398515	0.005881865
**TBRG1|84897**	**-1.38213156**	**0.501816448**	**0.005882552**
C17orf108|201229	-0.943897422	0.342907574	0.00591188
NUP155|9631	0.818638966	0.297434868	0.005917282
CHKB-CPT1B|386593	-0.744554132	0.270525062	0.00591863
PRMT5|10419	1.180618543	0.430303084	0.006075276
CYP2D6|1565	-0.488950833	0.178259277	0.006089563
FES|2242	-0.879643778	0.320947085	0.006129445
RAB5C|5878	1.216422478	0.443874523	0.006135236
SPPL2B|56928	-1.158302888	0.422926669	0.006166772
HNF1A|6927	-0.404351868	0.147721094	0.006195225
TEKT3|64518	-0.42149351	0.15406976	0.006224177
CIRBP|1153	-1.275656478	0.466311345	0.006226051
YME1L1|10730	1.375341617	0.502769264	0.006227945
CYP2C8|1558	-0.446859379	0.163404987	0.006244207
CYP21A2|1589	-0.412778069	0.151236034	0.006345677
WASH2P|375260	-1.104644476	0.40495244	0.006375099
OCM|654231	-0.688673201	0.252560081	0.006395764
SLC25A4|291	1.358966833	0.498410387	0.006399014
DPM1|8813	1.199471452	0.440540272	0.006474529
PCYOX1|51449	1.466206142	0.53852996	0.00647688
PCDHGA2|56113	0.445815248	0.163749084	0.006478002
MMGT1|93380	1.017305873	0.373730391	0.006488197
CDK4|1019	1.55621756	0.571730346	0.006489929
DDX27|55661	1.755938746	0.645322493	0.006507981
DDX21|9188	0.712793704	0.26199741	0.006516089
CCDC101|112869	-1.193000393	0.438539734	0.006520438
PNP|4860	1.295430276	0.476329552	0.006535909
HEXDC|284004	-0.971106772	0.357153785	0.006547649
ZNF446|55663	-1.310860288	0.48219978	0.006557858
FLJ39609|100130417	-0.41560041	0.152893504	0.006563129
UBQLNL|143630	-0.441962158	0.162619416	0.006572352
MST4|51765	0.531688942	0.19597959	0.006668089
DACT2|168002	-0.392080717	0.144605732	0.006700431
VILL|50853	-0.661010986	0.244007751	0.006749135
RNF135|84282	-1.511730926	0.558068518	0.00675143
C3orf26|84319	1.160118573	0.428275949	0.006752481
DHX36|170506	1.195369139	0.441474094	0.006775598
DNAJA3|9093	1.709957132	0.631548163	0.006777898

CCDC33|80125	-0.340673028	0.125910941	0.006816745
GLOD4|51031	1.73535212	0.641397968	0.006818567
HSD11B1L|374875	-0.941252777	0.34797558	0.00683169
IDUA|3425	-0.640921253	0.237032954	0.006852408
MZF1|7593	-0.895177496	0.331070173	0.006853287
CROCCL1|84809	-1.199894171	0.444132904	0.006899518
PCMT1|5110	1.58927589	0.588632714	0.006935096
SNRNP35|11066	-1.141811442	0.423091209	0.006960338
WDYHV1|55093	1.358411448	0.503382896	0.006963914
WASH5P|375690	-1.156970229	0.428988814	0.006997344
MRPL37|51253	1.595857579	0.592369533	0.007059514
SGCE|8910	0.512972756	0.190457365	0.007073305
FAM111A|63901	-1.268304351	0.47091091	0.007074858
ZNF10|7556	-0.933163492	0.346590043	0.007093707
ZNF140|7699	-1.285322946	0.478118635	0.007181836
AQP2|359	-0.468213453	0.174167806	0.007181914
WDR61|80349	1.659518798	0.617360423	0.007186258
COL18A1|80781	-0.845828665	0.314924093	0.007235258
GOLGA8F|100132565	-0.695660331	0.259022464	0.007237495
VAT1|10493	0.905489612	0.337237484	0.007252532
EIF4A3|9775	1.381909959	0.515413853	0.007336617
GPR113|165082	-0.860755513	0.321375618	0.007398646
NOXA1|10811	-0.583665004	0.218048707	0.00743369
GART|2618	1.223982334	0.45748492	0.007462691
KIAA1161|57462	0.610194196	0.2280758	0.007463994
FOXD1|2297	0.424181585	0.158566178	0.007470528
PSMD1|5707	1.615555163	0.603940413	0.007472388
ASB9|140462	0.723320219	0.270491997	0.007493211
NDUFS1|4719	1.190948482	0.445404375	0.007498398
ELMOD3|84173	-1.505897613	0.563274572	0.007507101
ERAP2|64167	-0.343767644	0.128694517	0.007558261
PDE9A|5152	-0.822888864	0.308209652	0.00758739
TTC9C|283237	1.907349611	0.714578958	0.00760336
TMX2|51075	1.605359697	0.601826912	0.007642302
NPDC1|56654	-0.612443039	0.229628947	0.007650923
NEUROD2|4761	-0.616798368	0.231343045	0.007672238
EIF2B3|8891	1.971696179	0.740681324	0.00776771
NPIPL3|23117	-0.557573616	0.209732175	0.007848865
USH2A|7399	-0.451970209	0.170015014	0.00785095
FAM193B|54540	-1.000218622	0.376331866	0.00786502
C9orf84|158401	-0.438515485	0.16532764	0.007992115
**BDNFOS|497258**	**-0.736747975**	**0.278626399**	**0.008188074**
PDZK1P1|728939	-0.376128955	0.14228824	0.008207081
MAN2A1|4124	0.630856484	0.238787921	0.008243967
LOC284900|284900	-1.110088411	0.420436307	0.00828266
TXNRD2|10587	-1.042767013	0.395292534	0.008340572
MRPL33|9553	1.06823752	0.404984777	0.008346481
PRIM1|5557	0.699848305	0.265415615	0.008369178
PTPN18|26469	-0.993304953	0.376999333	0.008419535
SLC35E2|728661	-1.768533935	0.671282828	0.008424677
LDLR|3949	0.715178001	0.271486443	0.00843098
C8orf79|57604	-0.387423797	0.147080284	0.008436128
SLC22A23|63027	-0.944467766	0.358638595	0.008451467
MUSTN1|389125	-0.519885445	0.197435946	0.008458817
FGF1|2246	-0.471812174	0.17919094	0.008463097
IDI1|3422	1.051853889	0.399646812	0.008489419
DERL1|79139	1.956794399	0.743669779	0.008506696
LMF1|64788	-0.77058638	0.292887687	0.0085135
MYH3|4621	-0.544643403	0.207121451	0.008548923

PYY|5697	-0.246407162	0.093804931	0.008619098
THOC4|10189	1.102506054	0.419800872	0.008632896
C22orf27|150291	-1.11046522	0.423022173	0.008662971
JMJD7-PLA2G4B|8681	-0.843401064	0.321591944	0.008726641
F10|2159	-0.597374991	0.227963037	0.008780336
CLSPN|63967	0.550164199	0.210030906	0.008807323
ARRDC4|91947	-0.791941928	0.302403379	0.008823286
FERMT2|10979	1.098437378	0.419650604	0.00885753
ZMAT2|153527	1.86700656	0.713460581	0.008874998
PPIL1|51645	1.23768419	0.472978893	0.008876217
AP2B1|163	1.662462086	0.635367693	0.008882714
PPT2|9374	1.470047442	0.561865164	0.008887007
CCDC48|79825	-0.589868257	0.225490801	0.008898468
SLC5A9|200010	-0.479225627	0.183317176	0.008943971
**PDLIM3|27295**	**0.383761369**	**0.14682369**	**0.008955284**
MTHFSD|64779	-1.470813932	0.562870429	0.008973567
**ZNF596|169270**	**-0.955900968**	**0.365965521**	**0.009001495**
MBTPS2|51360	1.182791064	0.453001318	0.00902752
USP9X|8239	1.379474602	0.528439854	0.009041879
WFDC6|140870	-0.323278693	0.123847595	0.009046431
ZSCAN4|201516	-0.636103185	0.243872693	0.009098222
LOC390595|390595	-0.708518609	0.271836711	0.009149619
UCP3|7352	-0.774568465	0.297299989	0.009178231
RABEP2|79874	-1.158576461	0.44471962	0.009182492
CUL2|8453	1.500654387	0.576051012	0.009185494
CIRH1A|84916	1.633674984	0.627506671	0.009229384
PDZK1|5174	-0.321974639	0.123706556	0.009248503
MGC42105|167359	-0.454924723	0.174802463	0.009254479
VPRBP|9730	1.18917315	0.456996609	0.009264166
NANOG|79923	-0.53132168	0.204330231	0.009313993
TNFRSF10A|8797	0.589626849	0.226756407	0.009315167
RPS6KA3|6197	0.961586603	0.369826278	0.009319564
C4orf31|79625	-0.430159807	0.165471457	0.009333214
STRAP|11171	1.211217733	0.466084873	0.009357578
TNFSF12-TNFSF13|407977	-0.547377587	0.210729394	0.009389476
LOC730101|730101	0.588586803	0.226722031	0.00942962
HOXD13|3239	0.399495199	0.153914777	0.009443663
CSNK2A1|1457	1.335788329	0.514866377	0.0094746
GTPBP4|23560	1.427071368	0.550071072	0.009477272
NMRAL1|57407	-1.060329365	0.408727102	0.00948049
LOC219347|219347	-0.652103734	0.25142302	0.009496265
PLS1|5357	0.608026339	0.234604254	0.009550006
ZNF137|7696	-0.887284682	0.342378493	0.009554977
RHBDD3|25807	-1.104831203	0.426532188	0.00959022
UBQLN1|29979	1.491286737	0.576483423	0.009685258
INO80E|283899	-1.34247286	0.519015795	0.00969353
ZNF582|147948	-0.545019898	0.210858763	0.0097446
PLGLB2|5342	-0.683372974	0.264516172	0.009780835
RRAGC|64121	0.606486722	0.234850186	0.009810417
TMEM219|124446	-1.471546239	0.570028676	0.009836339
ETFA|2108	1.216318447	0.471170988	0.009837771
RPL18|6141	-0.984095079	0.381377647	0.009869478
FOLH1|2346	-0.544826214	0.211365981	0.009947648
HSF5|124535	-0.655936713	0.254507862	0.009958273
ADRBK2|157	-0.904992815	0.351145118	0.009958656
DSCR3|10311	1.500932073	0.582450306	0.009968292
CORO1C|23603	1.204495932	0.467541125	0.009988266
**APOL4|80832**	**-0.465935125**	**0.181133144**	**0.010101509**
MED30|90390	1.331936664	0.517905113	0.010117792

RNF19B|127544	0.825633638	0.321114386	0.010136106
PTPN20B|26095	-0.387278452	0.150656266	0.010151968
RPL32P3|132241	-1.011034971	0.393339903	0.010158556
PIP4K2C|79837	1.339626681	0.521559469	0.010213904
RPL13|6137	-0.868883378	0.338334491	0.010225192
CYTH2|9266	-1.385225599	0.539512703	0.010242015
SIGIRR|59307	-0.931011521	0.362755912	0.010273187
DVL2|1856	1.281872806	0.499676098	0.01030547
SCD|6319	0.69767617	0.271983948	0.010313498
TAF1C|9013	-1.291682991	0.503862606	0.010360422
IFT52|51098	1.262046127	0.492400412	0.010375756
FAM114A1|92689	0.8323391	0.324961598	0.010426745
EBNA1BP2|10969	1.408109819	0.549894105	0.010446371
CALU|813	1.137786352	0.444342769	0.010449006
ANKK1|255239	-0.406654831	0.158897718	0.010490616
APOBEC2|10930	-0.661285326	0.25853285	0.010532494
TAF2|6873	0.958958449	0.37510964	0.010573864
SH3BP2|6452	-1.1237278	0.439866008	0.010627789
LOC100133612|100133612	-0.557668917	0.218328228	0.010641116
NCRNA00174|285908	-0.685108167	0.268462304	0.010711526
ECT2L|345930	-0.542946525	0.213040959	0.010817034
RPL28|6158	-0.920814466	0.361510231	0.010861218
MCM4|4173	0.629742843	0.247279945	0.010875317
PLA2G6|8398	-0.788846422	0.309926376	0.010919316
SENP2|59343	1.074963502	0.42235464	0.010922518
C20orf134|170487	-0.510332045	0.200585874	0.010952606
ZNF541|84215	-0.342363806	0.134640185	0.010996641
TMEM114|283953	-0.404460182	0.15913034	0.01103176
WISP1|8840	0.510489188	0.200863544	0.011038661
ZBED5|58486	-1.308915426	0.515047401	0.011042549
HOXB13|10481	-0.220928601	0.086956887	0.011064085
GSTA1|2938	0.2456904	0.096785189	0.011132486
WDR44|54521	0.94400139	0.371977311	0.011155397
DYSFIP1|116729	-0.525795166	0.207491585	0.011275091
SLC26A4|5172	-0.424412527	0.167543815	0.011304466
CPNE3|8895	1.475164405	0.582572583	0.011336369
VKORC1L1|154807	1.397381578	0.551882286	0.011340483
SFRS2B|10929	-1.278118474	0.50488548	0.011357525
EXD1|161829	-0.655718984	0.259061938	0.011369627
MTFR1|9650	1.169373125	0.462046143	0.011378394
P2RY11|5032	-0.707904989	0.279861776	0.011423142
UBQLN2|29978	1.401763241	0.554285335	0.011440236
KIAA1279|26128	1.387802127	0.548793447	0.011444535
LRRC48|83450	-0.400503928	0.158409968	0.01146246
P4HB|5034	-1.34164885	0.530762387	0.01147873
UBP1|7342	1.510863123	0.597727253	0.0114819
HOXA13|3209	0.394429652	0.156072676	0.011497016
GMCL1|64395	0.79776044	0.315751775	0.011519128
ADAM22|53616	0.422799341	0.16735844	0.011526764
HDAC3|8841	1.395081308	0.55230467	0.011539344
NCRNA00167|440072	-0.654368706	0.259170191	0.011574406
ZFP2|80108	-0.763003664	0.302302591	0.011603675
PCP2|126006	-0.381413519	0.151250409	0.011677749
NR1H2|7376	-1.349429636	0.535300688	0.011706125
TTC16|158248	-0.464311976	0.184228744	0.011725436
FLJ42627|645644	-0.612606333	0.243098484	0.011735737
NCRNA00204B|286967	-0.503878404	0.200144791	0.011816768
C1orf175|374977	-0.409291651	0.162592268	0.011826194
LOC387646|387646	0.616088093	0.244744846	0.011826921

ATP6V1H|51606	1.611073328	0.640787021	0.011929927
ZNF862|643641	-0.766497189	0.304916898	0.011944138
PPP2R5D|5528	1.805303955	0.71828562	0.011959025
METTL11A|28989	1.131998946	0.450423965	0.011964643
TMEM38B|55151	0.496145341	0.197479027	0.011991475
MAPRE1|22919	1.48713065	0.592000479	0.012003447
CECR1|51816	-0.44043259	0.175338356	0.012008278
SRPK1|6732	1.073118351	0.427399401	0.012045478
SLC37A4|2542	-1.527440837	0.608421512	0.012056109
LAMA1|284217	-0.297454631	0.118504186	0.012070484
GTF2E1|2960	1.384776416	0.551766638	0.0120829
SMCR5|140771	-0.591659691	0.235810846	0.012105855
FAM18A|780776	-0.850212667	0.338885805	0.01211259
PEG10|23089	0.259736424	0.103547954	0.012129011
ZNF623|9831	1.086076092	0.433006179	0.012134004
HAT1|8520	0.896055841	0.357319434	0.012151412
WBSCR17|64409	-0.298462864	0.11912231	0.012227284
OCA2|4948	-0.24929874	0.099542696	0.01226453
SLC22A18|5002	-0.578891058	0.231359647	0.012344988
MAP2K5|5607	1.295651769	0.517893166	0.01235727
CCT4|10575	1.13352001	0.453165213	0.012372454
CSAD|51380	-0.721669378	0.288548935	0.012383289
CCDC159|126075	-0.66822706	0.267248148	0.012405342
AMY1A|276	-0.235026797	0.09400097	0.012410245
LOC646982|646982	-0.568560612	0.227484779	0.012442672
PATZ1|23598	-1.71897461	0.687903756	0.012459381
ATP6V1C1|528	1.496468052	0.599014972	0.012482055
C17orf93|360205	-0.23917772	0.095741135	0.012483599
LMOD3|56203	-0.586142097	0.23469326	0.012507896
AGXT2L2|85007	-1.081256665	0.433110414	0.012542851
RFC1|5981	1.202375626	0.481685836	0.012553809
SLC9A11|284525	-0.549986235	0.220439061	0.012597198
C3orf32|51066	-0.448357215	0.179838784	0.012663091
AMY2B|280	-0.388575798	0.155881297	0.012675189
TNPO3|23534	1.053749765	0.423198301	0.012775499
GPN1|11321	1.571669093	0.631242736	0.012781447
WASH7P|653635	-0.973063656	0.391078351	0.012840766
LCA5L|150082	-0.685080211	0.275413562	0.012865893
KTI12|112970	1.617925288	0.650600679	0.012889143
DFNB59|494513	-0.586955366	0.236259101	0.012978035
PRPF8|10594	1.17755405	0.474056079	0.012991677
AES|166	-1.041595276	0.419348787	0.012997421
APBB3|10307	-0.882461953	0.355340205	0.01301245
C20orf24|55969	1.522833734	0.613248472	0.013019894
MAP6|4135	-0.390394566	0.157232106	0.013031076
SP2|6668	1.060853496	0.427397726	0.013060238
LRRC20|55222	0.883500511	0.355999111	0.013073925
GSPT1|2935	1.415338621	0.5705098	0.013107636
DPH2|1802	1.554764809	0.62673343	0.013110872
SPATA20|64847	-0.837705839	0.337795183	0.013141098
CACNA1I|8911	-0.285742133	0.115245529	0.013159604
C1orf157|284573	-0.404995255	0.163348673	0.013162949
AGAP6|414189	-0.669688315	0.270150092	0.013177013
DDX46|9879	1.134387419	0.45771137	0.013197669
CDK2|1017	0.916780127	0.370064632	0.013236152
LOC729799|729799	0.462493508	0.186697916	0.013240688
KCNQ1|3784	-0.477285213	0.192855064	0.013329629
SNORA25|684959	-0.45432193	0.183594523	0.013338767
ARFGAP2|84364	-1.934026745	0.781577864	0.013341693

FOXD4L3|286380	-0.487907765	0.197178032	0.013344039
GTF3C4|9329	0.738563747	0.298545049	0.013365568
STX18|53407	-0.476671377	0.192776166	0.013410882
AFG3L2|10939	0.906694606	0.366749942	0.013426859
ANKRD49|54851	-0.975418488	0.394937203	0.013518614
C6orf153|88745	1.756545578	0.711324267	0.013533908
ZFP64|55734	1.055229317	0.427372944	0.01354509
ZNF415|55786	-1.041123487	0.421725187	0.01355954
C8G|733	-0.485171462	0.196530165	0.013560978
SNRNP40|9410	0.962262735	0.38993832	0.01359726
FAM195B|348262	-1.083841153	0.439285102	0.013614229
GRM6|2916	-0.45346196	0.183810848	0.013624912
CLUL1|27098	-0.374023437	0.151682115	0.013669336
CSDE1|7812	1.813552108	0.735515749	0.013675052
NCRNA00110|642976	-0.450944375	0.182896021	0.013679245
RPA1|6117	1.07383519	0.435572382	0.013688226
LHB|3972	-0.443395581	0.179972346	0.013751603
RCBTB2|1102	-0.978385381	0.397213384	0.01377327
FAM83C|128876	-0.389429071	0.158247054	0.013859168
XRN2|22803	1.026548125	0.417349238	0.013905858
PARG|8505	0.985039767	0.400591651	0.013933925
JMJD6|23210	1.493685673	0.607463067	0.013936679
CSNK2A1P|283106	0.652992255	0.26579138	0.014018659
PTPLB|201562	0.422797506	0.172114776	0.014030334
PLB1|151056	-0.472187129	0.192247888	0.014043965
TMEM220|388335	-0.594796783	0.24217745	0.014047869
FBXW4|6468	-1.016333757	0.413850055	0.014057062
AMY2A|279	-0.330590402	0.134619106	0.014059228
STAC3|246329	-0.652330308	0.265917971	0.014162069
LOC100288778|100288778	-0.831368748	0.338954423	0.014177082
ZNF32|7580	-1.072619108	0.4373616	0.014187659
PARD3|56288	1.103623686	0.450258229	0.014242412
SCAPER|49855	0.940632294	0.383827702	0.014259344
MAPK14|1432	1.632016584	0.665949873	0.014259465
ALDH16A1|126133	-1.372866259	0.560318374	0.014279516
STAR|6770	-0.474090355	0.193526915	0.014295996
HSPA14|51182	1.103791578	0.450913228	0.014369057
GPRC5B|51704	0.493059633	0.201451443	0.014383681
CASK|8573	1.18365413	0.483626262	0.014386858
ZSWIM7|125150	-1.268949521	0.51850547	0.014392244
CYP2D7P1|1564	-0.591770836	0.241924775	0.014441319
MUT|4594	1.060779379	0.433903724	0.014495883
GANAB|23193	1.310525834	0.536245129	0.014529726
TBC1D3B|414059	-0.675282108	0.276480524	0.014589083
COPS4|51138	1.183153748	0.484612097	0.014628619
NUP93|9688	1.384179785	0.567321815	0.014693386
LDLRAP1|26119	-1.231415278	0.504812042	0.014713547
RALBP1|10928	1.37277465	0.562780659	0.014716919
ZNF193|7746	-0.998952301	0.409535025	0.01471838
RASL10B|91608	0.512595133	0.21021346	0.014750376
CHADL|150356	-0.464592742	0.19053176	0.014752419
ACCS|84680	-0.621644236	0.254954467	0.014758322
FXR2|9513	1.401090357	0.574747577	0.01477903
NADSYN1|55191	-1.007102406	0.413180069	0.014791592
C3orf62|375341	-1.056633477	0.433558996	0.014804938
TTC23|64927	-1.265000444	0.519123764	0.0148179
LRRC56|115399	-0.629994801	0.258681277	0.014874951
TRA2A|29896	-1.191713435	0.489378679	0.01488537
ATHL1|80162	-0.376501976	0.154719135	0.014955515

C6orf97|80129	-0.332024119	0.136460824	0.014969776
ZNF787|126208	-1.059290796	0.435400802	0.01497804
CBARA1|10367	1.962282849	0.806579319	0.014980692
SFXN1|94081	1.383621975	0.568751842	0.014985303
ITGB3|3690	0.396015004	0.162823442	0.01500848
LINGO4|339398	-0.336334045	0.138341424	0.015049393
EGFL8|80864	-0.507651705	0.20882921	0.015059624
CHRNA10|57053	-0.65683733	0.270254536	0.015080519
MPHOSPH8|54737	1.178861133	0.485228917	0.015119882
C6orf217|100131814	-0.475495945	0.195754309	0.01513867
SCN11A|11280	-0.455839937	0.187733004	0.015176947
LOC100130872|100130872	-0.533678142	0.219801514	0.015182331
KLKB1|3818	-0.304715942	0.125520388	0.015198212
BAZ2A|11176	1.350208834	0.556638496	0.0152811
FAM83D|81610	0.553178531	0.228061687	0.015284563
C1orf159|54991	-1.007305147	0.415448114	0.015324282
GJC1|10052	0.57600882	0.237576715	0.015328759
SLC39A2|29986	-0.306885106	0.126607881	0.015354745
MCM2|4171	0.659306939	0.272002996	0.015355007
FAM108B1|51104	0.986791058	0.407171774	0.015370686
BMF|90427	-0.755347371	0.311722358	0.015386934
USP17|391627	-0.431088933	0.177920496	0.015395887
LTB4R|1241	-0.52022041	0.214715836	0.015400045
PTGDS|5730	-0.344090436	0.14203124	0.015408225
SEC16B|89866	-0.424392389	0.175188145	0.015414342
ZBTB48|3104	-1.164050231	0.480588231	0.015429559
ANAPC4|29945	-0.553860826	0.228774228	0.015478171
EFTUD1|79631	1.135034759	0.468874429	0.01548789
HOXD1|3231	0.332784832	0.1375877	0.015575626
YRDC|79693	1.479765745	0.611846747	0.015583638
TMEM65|157378	0.803014515	0.332131475	0.015616332
ATP6V1A|523	1.242402571	0.513964172	0.015636377
ESYT2|57488	1.359937851	0.56264773	0.01564762
SNX30|401548	0.81267782	0.33624411	0.015652111
SIRT6|51548	-1.322745805	0.547670745	0.015725671
NUP205|23165	0.981091264	0.406240922	0.015733143
LPAL2|80350	-0.443965053	0.183856949	0.01574684
SERINC2|347735	-0.488133395	0.202151944	0.015748796
KLHL17|339451	-0.769782033	0.319197506	0.015881969
AGPAT9|84803	0.456555445	0.189409728	0.015934523
ORM1|5004	-0.188751936	0.078333147	0.015969803
HSF4|3299	-0.463680867	0.192490387	0.016002783
CPNE7|27132	-0.339645137	0.141083672	0.016066466
CISD1|55847	1.36037268	0.56526829	0.01610195
C20orf177|63939	0.671800743	0.279303652	0.016160513
PJA1|64219	0.781310748	0.324920807	0.016189334
METTL2A|339175	1.428039053	0.594167651	0.016242174
CLCN2|1181	0.812002812	0.337928709	0.016266435
SOAT2|8435	-0.44145263	0.183767486	0.01629525
PILRB|29990	-0.698516651	0.290797094	0.016302332
NUP85|79902	1.46742051	0.611649462	0.016434524
CCDC84|338657	-0.739096325	0.308170235	0.016469669
RPS9|6203	-1.047582708	0.436913121	0.016498772
PTPN1|5770	1.289147033	0.537676183	0.016501651
MPG|4350	-1.143741785	0.477033311	0.016502241
PLCH2|9651	-0.384112244	0.160333175	0.016588081
EIF5|1983	1.400044048	0.584470298	0.01660194
C10orf68|79741	-0.453434606	0.189388739	0.016656642
SRP14|6727	-1.281948	0.535577074	0.016684721

DCST2|127579	-0.392176972	0.163891589	0.016715577
CDR2L|30850	0.877289244	0.366654172	0.016725431
SLC4A1|6521	-0.411860725	0.172152533	0.016737792
IHH|3549	-0.166854448	0.069752725	0.016752961
HSN2|378465	0.628245415	0.262707519	0.016783098
LOC90246|90246	-0.399249168	0.166966603	0.016793778
SLC25A5|292	0.792638571	0.331649109	0.01684876
CHAC2|494143	0.61636098	0.258040405	0.016911694
JMJD5|79831	-1.181068779	0.494620019	0.016948155
FRYL|285527	-1.092586972	0.457748981	0.016992533
ABHD3|171586	0.822987131	0.344862542	0.017013273
PTHLH|5744	0.452266947	0.189528237	0.017019908
FAM22A|728118	-0.674728527	0.28276048	0.017022595
ABCG5|64240	-0.491868164	0.20622157	0.017072432
EIF4EBP3|8637	-0.651037961	0.27297502	0.017080391
BCHE|590	0.317060189	0.132987565	0.017119363
HSPA1A|3303	0.656387798	0.275329293	0.017125135
RACGAP1|29127	0.675872444	0.283535928	0.017138279
LOC91316|91316	-0.667757276	0.280185412	0.017159639
KIAA1683|80726	-0.385841411	0.162031262	0.017252706
RIOK1|83732	1.242004865	0.521821421	0.017306347
PYCARD|29108	-0.553600798	0.232664127	0.017340893
TCP1|6950	1.410755072	0.593064133	0.017371154
LRRN4CL|221091	-0.421087834	0.177022153	0.017372415
PGAM5|192111	0.783706059	0.329465047	0.017372729
RAPSN|5913	-0.404378028	0.170007132	0.017378782
RNF167|26001	1.810526506	0.761245711	0.01738921
TCEB1|6921	1.573861182	0.661744839	0.017390286
LOC100144604|100144604	-0.264717697	0.111303822	0.017391052
JPH1|56704	0.336651763	0.141562127	0.01740115
SAMD4B|55095	1.054951978	0.443734338	0.017433256
EIF4G1|1981	1.103712085	0.464279473	0.017441884
EIF1B|10289	0.932436342	0.392414809	0.017494362
CCNL2|81669	-0.763906115	0.321718744	0.017574962
FLJ40852|285962	-0.610544279	0.257134872	0.017576897
SLC35B2|347734	1.161175824	0.489067719	0.017583978
TINF2|26277	1.474039501	0.621052949	0.017622673
PEX26|55670	0.745997279	0.314411794	0.017659753
PRKG2|5593	0.376535877	0.15872377	0.017679094
C20orf151|140893	-0.535779486	0.225871234	0.017689352
AADACL4|343066	-0.425823018	0.179523715	0.01769402
C17orf48|56985	-1.233702953	0.520287981	0.017730794
STOML2|30968	1.440692836	0.607601213	0.017734474
MAP4|4134	1.531412941	0.645920488	0.017744827
POLR1A|25885	0.986433681	0.416111769	0.017759341
TIAF1|9220	-0.689495374	0.290928538	0.017788955
RPP14|11102	1.546131234	0.65242827	0.017797214
PGK1|5230	0.643690887	0.271814044	0.017878271
KDM6A|7403	0.839140288	0.354461949	0.017915326
SRPX|8406	0.426631199	0.1802675	0.017949597
ELP3|55140	-0.520071456	0.219836212	0.01799493
GUSBP1|728411	-1.088383686	0.460383866	0.018074973
RSAD1|55316	-1.300278285	0.550189919	0.018111717
SLC25A34|284723	-0.510963977	0.216228079	0.018123818
DHX58|79132	-0.6060943	0.256544473	0.018150618
POLM|27434	-1.384069677	0.586364175	0.018253923
SNX12|29934	0.671483803	0.284659869	0.018329257
NCRNA00093|100188954	-0.438603273	0.185995484	0.018366796
TARBP1|6894	-0.705160215	0.299049242	0.018373345

SSRP1|6749	1.12169093	0.475716329	0.018378706
IL32|9235	-0.427665933	0.181379117	0.018380796
ARMC1|55156	0.975077924	0.413647097	0.018409852
GEMIN8|54960	-1.040295171	0.441328542	0.018413829
CTNNB1|1499	0.76675746	0.325463099	0.018478052
GOLGA6L9|440295	-0.601954736	0.255533248	0.018488803
DNAJB13|374407	-0.257237907	0.109214664	0.018505655
KIAA1429|25962	1.690900713	0.717901275	0.018505825
UBXN11|91544	-0.718358022	0.305053479	0.01852972
PLXNB1|5364	-0.961978866	0.408568374	0.018547078
WDR67|93594	0.728331644	0.309462186	0.018595605
CDK10|8558	-1.046575259	0.444880375	0.018648346
DHX16|8449	1.524440103	0.648065637	0.018658084
UTP18|51096	1.010514205	0.42967047	0.018681041
BLNK|29760	-0.74006842	0.314685856	0.018684378
GGTA1|2681	-0.357622856	0.152074162	0.018691127
TRAF5|7188	-0.639711127	0.272063019	0.018706216
KRTAP5-9|3846	-0.379876716	0.161635103	0.018762735
GGT3P|2679	-0.361566159	0.153852487	0.01876921
FOXD4L1|200350	-0.507321973	0.215901378	0.018784222
RAE1|8480	1.238886023	0.527347255	0.018809788
LTB|4050	-0.384370896	0.163630008	0.018822669
RAP1GDS1|5910	1.416058894	0.602975884	0.018851715
HOMER2|9455	-0.490146005	0.208731274	0.018863605
CD1A|909	-0.328768854	0.140096178	0.018938629
UBE2G1|7326	1.407658925	0.60001759	0.018974609
MDM4|4194	-0.978789	0.417241919	0.018983496
CNR2|1269	-0.513036137	0.218724826	0.018997604
TOPBP1|11073	0.875907859	0.373452514	0.019004993
LARS2|23395	0.972277807	0.414692705	0.019048821
KLK14|43847	-0.238718756	0.101885926	0.019129367
APOM|55937	-0.744229012	0.317658804	0.019136739
GGTLC2|91227	-0.311063652	0.13280659	0.019168869
C2orf63|130162	-0.64037213	0.273431925	0.019181737
TNFRSF6B|8771	-0.403006814	0.17208271	0.019183968
ORC1L|4998	0.634451401	0.270917627	0.019187864
RASSF7|8045	-0.748664208	0.319709307	0.019195989
MED18|54797	-1.319165049	0.563349667	0.019198956
ABCB7|22	1.149786468	0.491226566	0.019250518
CAPN3|825	-0.476493598	0.203674181	0.019310117
WDHD1|11169	0.693903268	0.296828839	0.019401681
B4GALT1|2683	-0.462645414	0.197933559	0.019419573
ZNF692|55657	-0.661189306	0.283056104	0.01949672
NXF3|56000	-0.255418614	0.109374753	0.019529781
CTPS|1503	1.036264483	0.443899525	0.01957179
ADCY4|196883	-0.540629087	0.231654649	0.019607674
TNNT2|7139	-0.284153332	0.121798379	0.019649131
POU5F1|5460	-0.282863943	0.121250827	0.019654308
IMMT|10989	1.098834924	0.471091655	0.019672882
CXorf56|63932	0.75695538	0.324530975	0.019676527
FAM124A|220108	-0.550009324	0.235822459	0.01968478
C15orf48|84419	-0.406139939	0.174139398	0.019686538
MORN3|283385	-0.41664018	0.178756918	0.01976581
XPO7|23039	1.683604811	0.722447476	0.019784054
KLHL23|151230	0.412384167	0.17695855	0.019785031
ANAPC7|51434	-1.583034741	0.679759119	0.019868751
SFRS9|8683	1.166316454	0.501006088	0.01991483
LOC283663|283663	-0.548858178	0.23583837	0.019951212
FOLH1B|219595	-0.38720379	0.166381836	0.019954629

LOC441869|441869	-0.292927825	0.125912409	0.019995021
C3orf10|55845	1.803891716	0.775499334	0.02001303
LEPROT|54741	0.481526268	0.207019367	0.020018752
ITFG1|81533	0.810287399	0.348425224	0.020041479
PLEKHF1|79156	-0.637818498	0.27426465	0.020042114
TMEM31|203562	-0.401669663	0.172759917	0.020071077
LOC100129534|100129534	-0.489648148	0.210600067	0.0200712
RALGAPA2|57186	0.555862971	0.239105542	0.020084803
DUOXA2|405753	-0.297842997	0.128122025	0.020088957
FOXD4L6|653404	-0.481597883	0.207204899	0.020111609
C16orf13|84326	-0.708548204	0.304941375	0.020149331
CCDC36|339834	-0.4982104	0.214418523	0.020150065
ARPC2|10109	1.312348701	0.564897314	0.020170382
HTR7P1|93164	0.758516007	0.326508111	0.020172956
PRR22|163154	-0.536251013	0.230844938	0.020179566
TMSB10|9168	-0.913836339	0.393463982	0.020203808
MMP21|118856	-0.631558434	0.271931671	0.020206596
CHIA|27159	-0.239967272	0.103331095	0.020216022
ECE2|9718	0.980960893	0.422411676	0.020217519
PRR11|55771	0.44835666	0.193127422	0.020256706
C1QTNF7|114905	-0.419588762	0.180738462	0.020258555
RPRD1B|58490	1.046161249	0.450740788	0.020287816
PCDHGA4|56111	0.372046923	0.160352287	0.020330888
MDH2|4191	0.853025946	0.367660935	0.020333143
RNF207|388591	-0.498365552	0.214902262	0.020393218
FIZ1|84922	-1.204365278	0.519430283	0.020415249
TSPAN10|83882	-0.445747832	0.19232131	0.020464293
C1orf204|284677	-0.454112906	0.196047384	0.020539571
C9orf142|286257	-0.621188258	0.26830724	0.020601309
TNFRSF14|8764	-0.54148397	0.233893171	0.020607953
HSP90AB1|3326	1.306979732	0.564553135	0.02060913
KRTCAP2|200185	-0.756359231	0.326803357	0.020644861
CYP17A1|1586	-0.280710461	0.12132802	0.020686979
MCF2L|23263	-0.602792602	0.260625592	0.020729896
MST1P9|11223	-0.39042807	0.168870802	0.020778083
TXNRD1|7296	0.727547615	0.314764964	0.020810793
POLA2|23649	0.973713314	0.42128339	0.020816157
CDK9|1025	-1.485799572	0.64308876	0.020865391
KCNMB2|10242	-0.375326256	0.162476877	0.020886563
RNF215|200312	-1.07344536	0.464706616	0.020891362
HIST1H4J|8363	-0.556117039	0.240798505	0.020917488
COX19|90639	-0.907354391	0.392908195	0.020925317
POU5F1B|5462	-0.29821363	0.12918456	0.020975182
ACAD10|80724	-1.314048398	0.56929838	0.020988613
DHRS12|79758	-0.825808039	0.357776365	0.020989781
PELO|53918	1.001753472	0.434095934	0.021017074
DCTPP1|79077	0.924565076	0.400665668	0.021022926
LIMS3|96626	-0.237505186	0.102963287	0.021071674
C22orf46|79640	-0.884607409	0.383555048	0.021091779
ST6GALNAC3|256435	0.415048124	0.179975647	0.021103095
PLAC8L1|153770	-0.46079946	0.199833289	0.021115136
ZNF578|147660	-0.565421784	0.245322591	0.021177257
HCCS|3052	1.490580475	0.646821706	0.021196339
C21orf99|149992	-0.403656215	0.175234654	0.021249705
ZNF837|116412	-0.560930398	0.243552113	0.021271941
IRAK4|51135	-1.246496403	0.541239687	0.021276618
TIMELESS|8914	0.772246351	0.335336226	0.021284377
NDUFAB1|4706	1.552712367	0.674275936	0.021290955
OBSCN|84033	-0.394414114	0.17129972	0.021308139

GEMIN5|25929	1.01850877	0.442486564	0.021347321
SLC2A9|56606	-0.616041995	0.267644225	0.021350982
PPOX|5498	-0.811341529	0.352543913	0.021369518
C21orf2|755	-0.762966188	0.331538404	0.021375215
GSTA2|2939	0.325938544	0.14163761	0.021379501
BARD1|580	0.447655613	0.194677188	0.021477898
RNASET2|8635	-0.505411057	0.219794917	0.021478462
MUC6|4588	-0.175193733	0.076192648	0.021484949
WDR89|112840	-1.202868023	0.52334098	0.021536739
SLC16A8|23539	-0.49351893	0.214736615	0.021547431
TNNI2|7136	-0.457110304	0.198927154	0.021568741
TDRD3|81550	-1.023238644	0.445416901	0.021603915
BRF1|2972	-1.046022362	0.455467237	0.021642062
LRCH3|84859	1.052479972	0.458408345	0.021679073
APEH|327	0.980088168	0.426890694	0.021682963
MAGEE1|57692	0.382869789	0.166779424	0.021695008
CHCHD3|54927	1.114389913	0.485438687	0.021696775
CD74|972	-0.412840698	0.17993648	0.021769307
CA2|760	0.374542316	0.163267925	0.021788495
ELAC1|55520	-1.097367111	0.478360913	0.021789637
KLK1|3816	-0.259170826	0.112991177	0.02180623
ASB12|142689	-0.302814824	0.132027357	0.021814845
GOLT1A|127845	-0.316956255	0.13823156	0.021851634
UCKL1AS|100113386	-0.457215288	0.199433868	0.021873009
SLC13A5|284111	-0.231340572	0.100922438	0.021890585
LOC100272146|100272146	-0.566626548	0.247223189	0.021907744
GOLGA9P|283796	-0.481361981	0.210151577	0.02198965
SFRP5|6425	-0.244109192	0.106584679	0.022004855
DOCK7|85440	0.674437439	0.294485394	0.02200835
DAXX|1616	1.829442796	0.798923043	0.022027918
SUFU|51684	-1.043641185	0.45579265	0.02203709
GGTLC1|92086	-0.352643764	0.154078507	0.022095172
KCNQ1OT1|10984	-0.486596872	0.212613156	0.022099758
EDIL3|10085	0.346453018	0.151382379	0.022102871
C19orf66|55337	-0.619266653	0.270693058	0.022154536
GATC|283459	0.648350154	0.283468007	0.022183714
SEPT12|124404	-0.533212691	0.233141232	0.022191143
NCRNA00120|55389	0.545559317	0.23856441	0.022204997
MED1|5469	0.98460459	0.430797128	0.022281113
COMMD2|51122	1.17180989	0.512761708	0.022295751
FKBP6|8468	-0.498235277	0.218160102	0.022382954
IARS|3376	0.861821426	0.377431295	0.022407627
IGSF10|285313	0.373820023	0.163734169	0.022425003
MAD2L1BP|9587	1.77258621	0.776502448	0.022443182
ELOVL5|60481	0.706962418	0.309859729	0.022515549
TSR1|55720	1.318501543	0.578161247	0.022577557
SLC24A6|80024	-0.771804228	0.338453486	0.022584869
PXK|54899	0.653515812	0.286599069	0.02259322
RHOA|387	1.610014038	0.706199175	0.022617775
TEX14|56155	-0.578375585	0.253696864	0.022620144
PDCD10|11235	0.844809518	0.370588199	0.022628755
THOC6|79228	-1.027246189	0.450712533	0.022657528
LOC650368|650368	-0.712191142	0.312493784	0.022663678
LOC100130015|100130015	-0.728079968	0.319512711	0.022683722
NRF1|4899	-1.704190342	0.74801309	0.022709337
RPS3|6188	-1.138800476	0.499851876	0.022710145
ATAD2|29028	0.585036751	0.256794227	0.022712776
PHF11|51131	-0.905883609	0.397705444	0.022739973
FKBP5|2289	0.42400769	0.186190338	0.022769461

PLIN5|440503	-0.294301297	0.129235407	0.022771316
PIK3R1|5295	-0.410471185	0.180272951	0.022789646
MDC1|9656	0.968640935	0.425486429	0.022813139
THBS2|7058	0.353657668	0.155378994	0.022840203
HRASLS5|117245	-0.301159249	0.132354619	0.02288215
NSUN2|54888	1.47869987	0.649871565	0.022883595
NUDCD1|84955	0.797236214	0.350393302	0.022890278
PRG4|10216	-0.491172732	0.215935592	0.02292837
CDK11A|728642	-0.987549975	0.434245229	0.022955421
C1orf126|200197	-0.455484709	0.200299546	0.022964924
ITLN1|55600	-0.322742212	0.141926045	0.022964976
ZRSR2|8233	-0.727305493	0.319847076	0.022971017
USP14|9097	1.274913242	0.560704274	0.022979745
CATSPER4|378807	-0.59480001	0.261597609	0.022982759
CHRNA5|1138	0.362822504	0.159590921	0.022998878
IL4I1|259307	-0.354976928	0.156146629	0.023004715
GMPS|8833	1.027598754	0.452146777	0.023043628
PCSK1|5122	0.264245965	0.11630265	0.023083216
LOC100130933|100130933	-0.215291984	0.094785469	0.023125156
TTC28|23331	-0.734458525	0.323363216	0.02312814
ASCL2|430	-0.343702634	0.151346116	0.023148737
ZMYND12|84217	-0.369804031	0.162851572	0.023158835
AARS|16	1.194205677	0.525928503	0.023167469
PSMD2|5708	1.08547554	0.478089412	0.023180619
GOLGA4|2803	0.78965599	0.34780123	0.023181917
TMEM101|84336	-0.275987259	0.12156051	0.023185181
RNF122|79845	-0.917516487	0.40417364	0.02320123
DDX23|9416	1.505515825	0.66332317	0.023228425
MLLT6|4302	-1.279222538	0.563629547	0.023230903
DKC1|1736	1.232612772	0.543126464	0.023239362
ZNF814|730051	-0.746749018	0.329169309	0.023293489
TREH|11181	-0.289705415	0.127732816	0.023325681
C12orf47|51275	-1.41375053	0.623428019	0.023347223
SFRS8|6433	-1.453703073	0.641104215	0.023359769
PIN1L|5301	0.826990525	0.364780438	0.023384654
KLHL3|26249	-0.485956683	0.214369581	0.023395685
RPL27A|6157	-0.858103559	0.378538052	0.023397038
XRRA1|143570	-1.166298506	0.514643971	0.023437673
POLR3D|661	0.931051558	0.410871582	0.023448917
ANKAR|150709	-0.809512861	0.357369084	0.023500338
SBNO1|55206	0.542538395	0.239541946	0.023518857
DNAJC22|79962	-0.378774899	0.167305955	0.023576241
C1orf229|388759	-0.554704314	0.245023978	0.023581639
C3orf72|401089	-0.395550739	0.17476545	0.023615758
SFI1|9814	-1.032711507	0.456373186	0.023643932
NARFL|64428	-1.197217544	0.529137732	0.023661465
SBDS|51119	1.189618273	0.525831425	0.023675371
RAD21|5885	0.904683408	0.400121502	0.023757912
GOLGA2B|55592	-0.584916866	0.258730334	0.023776595
CLK4|57396	-0.907782486	0.401613873	0.023800376
KCNC4|3749	-0.563068204	0.249219066	0.023862842
DHRS7B|25979	-0.848981921	0.375794041	0.023872928
NENF|29937	-0.679137571	0.300711717	0.023918614
FAM49B|51571	0.702160225	0.31094614	0.023936871
COL6A4P2|646300	-0.29000947	0.128478694	0.023992002
IPPK|64768	1.197149423	0.530533517	0.024039271
CDK3|1018	-0.617671162	0.273823873	0.024087904
ATP11B|23200	0.450162007	0.199579812	0.024098902
GNA11|2767	0.450258094	0.199654748	0.024121813

HUWE1|10075	0.943002763	0.41826505	0.024161094
TMEM86B|255043	-0.890464015	0.395147757	0.024227873
SNORA39|677821	0.55397326	0.245859238	0.024245714
FARSA|2193	1.42293582	0.631679382	0.024282898
RNF114|55905	1.707467434	0.758453337	0.024369761
LOC643677|643677	-0.406509263	0.180642728	0.024426772
LOH12CR2|503693	-0.750048657	0.333393975	0.024465665
DR1|1810	1.017980685	0.452494169	0.024467356
GATS|352954	-0.682086252	0.303244822	0.024493903
POSTN|10631	0.343138567	0.15259746	0.024534652
INCENP|3619	0.754314668	0.335452392	0.02453484
TP53AIP1|63970	-0.281622725	0.125241932	0.024536067
ZNF497|162968	-0.727154168	0.323397982	0.024545545
CXXC1|30827	-1.105395637	0.491662766	0.024558332
SPG7|6687	-1.392203211	0.619259248	0.024565028
MRFAP1L1|114932	-1.203130852	0.535319483	0.024608053
SIL1|64374	-0.77173618	0.343435834	0.024633355
ARHGEF3|50650	-0.774864562	0.344829354	0.024633911
LOC149134|149134	-0.351737347	0.156589242	0.024688506
RC3H2|54542	0.616605905	0.274540175	0.024706651
KLHL31|401265	-0.468552825	0.208839543	0.024858018
KIAA1715|80856	0.727979502	0.324521261	0.024881257
FAM71F1|84691	-0.367924659	0.164102919	0.024959043
APOB48R|55911	-0.51343167	0.229080131	0.025008239
LOC375190|375190	-0.631992038	0.281990839	0.025014462
ZNF542|147947	-0.684513073	0.30543924	0.025021041
ADCY3|109	0.740725301	0.33066038	0.025081883
CA3|761	-0.392634359	0.175278485	0.025087015
PRR5|55615	-0.720016641	0.321488811	0.025114821
C20orf11|54994	1.614056494	0.720823374	0.025143917
PCDHGA11|56105	0.448209075	0.200280345	0.025227026
LRP2|4036	0.194216252	0.086788817	0.025233923
RRM1|6240	0.706568521	0.315779	0.02525099
PRKAR2A|5576	0.448903571	0.20066272	0.025279479
CAMK2G|818	1.523379376	0.680985752	0.025284973
DIAPH3|81624	0.477401781	0.213461032	0.025320207
TRIP13|9319	0.614937595	0.275042742	0.025365582
RALGAPB|57148	1.390741277	0.622091354	0.025378633
CRIPAK|285464	-0.653159916	0.292205562	0.025399271
RPS6KA1|6195	-0.94616073	0.423326942	0.02541347
C16orf80|29105	1.353878396	0.605775969	0.025420697
LGTN|1939	-1.120760533	0.501587227	0.025454927
C1RL|51279	-0.749091277	0.335277649	0.02546718
MRGPRX3|117195	-0.491816714	0.220152947	0.025484559
CIAPIN1|57019	1.455150722	0.651546783	0.02552398
NUDT18|79873	-0.79200904	0.35470688	0.025558506
CCDC21|64793	0.816479081	0.365685866	0.025566524
AGPAT3|56894	-1.369578933	0.613521871	0.025593628
REEP6|92840	-0.340119629	0.152388919	0.025620358
WWTR1|25937	0.575901048	0.258065	0.025640593
DGCR14|8220	-1.171197853	0.524989381	0.025687874
LOC643719|643719	-0.451990546	0.202607863	0.025690153
KCNRG|283518	-0.350420035	0.157085417	0.025696959
RRM2|6241	0.567205623	0.254312095	0.025724049
MPP4|58538	-0.504717242	0.226363725	0.025769114
NRIP2|83714	-0.673747141	0.302234285	0.025799176
AFG3L1|172	-0.854062632	0.383126099	0.02580096
ADAM17|6868	0.697685671	0.313031179	0.025826836
ASB2|51676	-0.466949192	0.209569696	0.025871636

RQCD1|9125	0.686610923	0.308179272	0.025883219
LGALS9C|654346	-0.475944211	0.213665636	0.025912624
KIF2A|3796	0.668433545	0.300106666	0.025925902
PGBD2|267002	-1.050119589	0.471609366	0.025969172
ZNF454|285676	-0.527656912	0.237039192	0.026012014
RASL11A|387496	-0.53646593	0.24103021	0.026032895
RINL|126432	-0.487651774	0.219119713	0.02604743
TRAM2|9697	0.897077595	0.403288441	0.026121079
ORM2|5005	-0.190154586	0.085501844	0.026149636
GPR89C|728932	-0.694038377	0.31224913	0.026235553
RUVBL1|8607	1.009211394	0.454049349	0.026236644
UBD|10537	-0.252298061	0.113560493	0.026303161
AS3MT|57412	-0.442346824	0.19911624	0.026313685
GUCY2C|2984	-0.343167108	0.154484593	0.026326057
CAGE1|285782	0.582947314	0.262461822	0.026345902
CFP|5199	-0.487746387	0.21967692	0.026399114
SNX3|8724	1.315094376	0.592418703	0.026427379
GUSBP3|653188	-0.714826911	0.32211198	0.026473965
PPAPDC1B|84513	-1.128312736	0.508554938	0.026509565
IL18BP|10068	-0.617761469	0.278488047	0.02653648
EVL|51466	-0.880399553	0.396893579	0.026539445
LRRC29|26231	-0.608341145	0.274346023	0.026594209
VCL|7414	0.934054415	0.421261158	0.026603887
C9orf103|414328	-0.517866584	0.233620808	0.026643793
PSD4|23550	-0.731346172	0.329934758	0.026647787
DYNLL2|140735	0.782000166	0.35282071	0.026662501
KIF21A|55605	0.757398064	0.341725261	0.026664481
AURKA|6790	0.600357792	0.270883148	0.026671097
ZFYVE28|57732	-0.584934775	0.263985096	0.026706085
LRRC69|100130742	-0.431837781	0.19489259	0.026707038
TOM1|10043	-0.791732096	0.357405148	0.026744763
LOC339047|339047	-0.620341539	0.280122393	0.026791827
PFDN5|5204	-0.98571749	0.44515179	0.026805387
ARL6IP4|51329	-0.867970748	0.39199974	0.02681416
BAX|581	0.97972075	0.442570964	0.026849204
GPCPD1|56261	-0.484795016	0.219201571	0.026991532
PPP1R3C|5507	-0.379839352	0.17176801	0.027011544
TREML3|340206	0.595287568	0.269267563	0.027052047
CENPO|79172	0.748156017	0.338467073	0.027075693
LOC100129637|100129637	-0.825561652	0.373490485	0.027077732
KIAA0090|23065	1.086869999	0.491840783	0.027119013
C14orf101|54916	-0.870156132	0.393872182	0.027158313
TNFRSF25|8718	-0.536074284	0.242675109	0.027173217
AGAP4|119016	-0.685587183	0.310382531	0.027185361
STAU1|6780	1.591337289	0.720826926	0.02726832
HEBP2|23593	-0.786977568	0.356482938	0.027271027
YDJC|150223	-0.928947875	0.420869589	0.02729934
CPB2|1361	-0.384269947	0.174098174	0.027299906
TRPM2|7226	-0.536660302	0.243259974	0.027375712
PCDHGA9|56107	0.357674729	0.162154618	0.027400567
PTPN9|5780	1.202204631	0.545125311	0.027427933
PTCD2|79810	0.982222339	0.445380372	0.027429113
AGPAT1|10554	1.248658112	0.566216294	0.027435364
CCDC17|149483	-0.267793399	0.12144399	0.027448664
POLR3A|11128	0.972131448	0.440937588	0.027475735
MLEC|9761	-1.10329862	0.500450774	0.027481496
RAP1GAP|5909	-0.441679189	0.200456446	0.027568868
PSMD3|5709	1.227850232	0.557323386	0.0275863
EDF1|8721	-0.794808989	0.360773838	0.027590016

ORAI3|93129	-1.000931784	0.45436535	0.027600191
WDR3|10885	0.926183091	0.420710577	0.027702537
CHCHD7|79145	0.828854986	0.376780746	0.027818696
MYO15B|80022	-0.401774184	0.182641787	0.027821562
ACYP2|98	-0.720899285	0.327747528	0.027838359
COLQ|8292	-0.543248859	0.246984897	0.027840785
CPT2|1376	1.069993524	0.48651307	0.027855819
PKIG|11142	-0.827321611	0.37620794	0.027870297
LOC642826|642826	-0.637026024	0.289689973	0.027878469
NECAB3|63941	0.79275236	0.360579964	0.027910078
TTC35|9694	1.20262493	0.547068142	0.027927079
XPOT|11260	0.910630039	0.414362536	0.027972988
POM121L9P|29774	-0.522769441	0.237880058	0.027976356
STARD4|134429	0.589210869	0.268127831	0.027984767
CNKSR1|10256	-0.863554621	0.392997888	0.02799533
TP53TG1|11257	-0.770881328	0.350985561	0.028068085
WDR13|64743	-0.944654808	0.430141815	0.028081398
DMRTC1|63947	-0.391678977	0.178404508	0.028131012
LOC284837|284837	-0.449893037	0.204935744	0.028142925
COLEC11|78989	-0.317338597	0.144562682	0.028151934
SPA17|53340	-0.424155382	0.193234832	0.028161769
TRHDE|29953	0.258723806	0.117874172	0.028169685
ZNF841|284371	-0.896109722	0.408523641	0.028269064
TRAPPC2P1|10597	-1.256761645	0.573051913	0.028299987
ENTPD2|954	-0.340313593	0.155198535	0.028324465
PPIF|10105	0.804468951	0.366937959	0.028351783
C20orf111|51526	1.350851313	0.616180158	0.028357904
LOC729375|729375	-0.732747489	0.334255984	0.028366899
C8orf76|84933	1.070651934	0.488503393	0.028401317
TMEM90A|646658	-0.387444307	0.176821253	0.028439951
TMEM55B|90809	1.347740089	0.615333932	0.028505528
C3orf24|115795	0.59345023	0.271130717	0.028611563
SQLE|6713	0.57478715	0.262673457	0.028653626
KPNA6|23633	1.194982872	0.546106089	0.0286559
LOC349196|349196	-0.295585442	0.135129352	0.028711448
GRM5|2915	-0.303707322	0.138859232	0.028730865
FBXO32|114907	0.47319119	0.216422556	0.028784694
AGAP5|729092	-0.545629143	0.249573905	0.02879785
SIRT3|23410	-1.162860272	0.531909916	0.02880114
WAC|51322	1.346498025	0.616015959	0.028829012
ATF7IP2|80063	-0.406705271	0.186076856	0.028838707
THSD1|55901	-0.600272743	0.274679334	0.028862591
UPF2|26019	1.002327715	0.458799474	0.028912689
POC1A|25886	0.846843483	0.387807857	0.028986677
SIM2|6493	0.277708205	0.127207043	0.029026994
C2orf82|389084	-0.416732344	0.190909978	0.029045122
MGC16703|113691	-0.356460399	0.163316804	0.029062959
ADAMTS6|11174	-0.308930517	0.14160747	0.029139244
LOC389634|389634	-0.376541588	0.172606857	0.02914657
RNMT|8731	1.038370559	0.475998184	0.029149478
KIAA1908|114796	-0.699408924	0.320754393	0.029219502
CELF6|60677	-0.40979201	0.187962205	0.029243896
AP4B1|10717	-0.931443275	0.427298696	0.029269239
DPY19L2P1|554236	-0.302619637	0.13888658	0.029339275
CCDC30|728621	-0.607005606	0.278589772	0.029342738
ZNF358|140467	-0.729990761	0.335088219	0.029368593
ANKS3|124401	-0.735795593	0.337758977	0.029371549
RBMS3|27303	-0.451957445	0.207474257	0.029377875
DGAT1|8694	-1.016266158	0.466571677	0.029394383

C22orf36|388886	-0.387598952	0.177955009	0.029400623
GTF2H3|2967	0.606560117	0.278488158	0.029402586
C20orf43|51507	1.955046904	0.897754615	0.029427825
ARHGEF10L|55160	-0.623095167	0.28617644	0.029457356
SART3|9733	1.479015966	0.679475548	0.029502789
MTHFD2|10797	0.667233423	0.306554363	0.029513544
ZHX3|23051	0.919395583	0.422516261	0.029555225
GPR176|11245	0.362355593	0.166572059	0.029602521
CSTF1|1477	1.387115245	0.63769141	0.029614088
ACSL5|51703	-0.283066621	0.13031428	0.029841772
CCDC42B|387885	-0.248304746	0.114361247	0.029913674
HSPA4L|22824	0.349931217	0.161170091	0.02991666
COL9A1|1297	-0.177920963	0.081946428	0.029917092
EFNA4|1945	-0.741098441	0.341344668	0.0299225
PLK1|5347	0.576999611	0.265887123	0.02999985
CCNA2|890	0.553169018	0.255009291	0.030066651
PAM|5066	-0.523154989	0.241276464	0.030137371
CORO6|84940	0.344933582	0.159177229	0.030236518
GAS5|60674	-0.541176801	0.249776615	0.030262072
NUP153|9972	0.824668655	0.380734747	0.03031175
PRDM15|63977	-1.228887206	0.567418254	0.030330145
ZGLP1|100125288	-0.43833833	0.202504696	0.030419594
SF3B3|23450	1.164007077	0.537776806	0.030427539
TBC1D10C|374403	-0.395178283	0.18258683	0.030438977
TMCO7|79613	0.818544385	0.37824717	0.030460797
PPM1D|8493	0.857245068	0.396155676	0.030471288
DCST1|149095	-0.488099753	0.225640552	0.030527807
MAP3K1|4214	-0.621607292	0.287372553	0.030535704
SLC25A35|399512	-0.316957124	0.14656022	0.030568811
ESR1|2099	-0.366392816	0.169436579	0.030585922
LOC728989|728989	-0.471354162	0.217977379	0.030587401
BCL7C|9274	-0.803440552	0.371556751	0.030590211
FAAH|2166	-0.522964592	0.241860309	0.03059823
LOC728875|728875	-0.56639923	0.262185093	0.030749225
UBAP2|55833	1.259909477	0.583461093	0.030821328
DUSP27|92235	0.381932845	0.176915698	0.030862512
FCRL6|343413	-0.338871581	0.156973704	0.030867294
DENND2C|163259	0.444635421	0.205982121	0.030880302
DHX33|56919	0.550772268	0.255175834	0.030896518
GNPTG|84572	-0.876448525	0.406176324	0.030943145
POM121L10P|646074	-0.565678459	0.262168631	0.030951994
GOLGA6L5|374650	-0.470793335	0.218325307	0.031053645
DNAJC4|3338	-0.772320642	0.358193823	0.03107173
SEC23B|10483	0.730910202	0.339055021	0.031104957
AMD1|262	0.971448406	0.450664021	0.031115426
GPHN|10243	0.859422958	0.398696014	0.031116122
TMEM217|221468	-0.7249671	0.336598842	0.03125574
SOLH|6650	-0.974763547	0.452591121	0.031260577
DIP2B|57609	1.082046792	0.502490505	0.031289797
VPS13B|157680	0.950215666	0.441380458	0.031332295
COG8|84342	0.956010505	0.444087309	0.031338057
C14orf182|283551	-0.521508042	0.242302941	0.031373922
STIP1|10963	1.22501164	0.569186222	0.031380354
RPL9|6133	-0.753078518	0.349950367	0.031400656
CDKL5|6792	0.383268028	0.178102858	0.031401477
KCP|375616	-0.266525644	0.123862001	0.031413498
ESYT1|23344	0.866519906	0.402765855	0.031442792
TBX19|9095	-0.610733759	0.283963091	0.031495898
CLK1|1195	-0.730202788	0.339563186	0.031522166

FBXL20|84961	1.082163063	0.503404328	0.031579773
TDRD6|221400	-0.40189765	0.187003332	0.031622749
MAPKAPK2|9261	-1.074499629	0.499985674	0.031629578
EFNA3|1944	-0.484326733	0.225484531	0.031718702
AKIRIN1|79647	0.898738243	0.418449229	0.031730978
C3orf19|51244	-1.032136917	0.480596792	0.031744346
C2orf89|129293	-0.265453713	0.123610194	0.031752969
SLED1|643036	0.521001852	0.242628486	0.031767536
FOXM1|2305	0.516384531	0.240484843	0.031772242
ZNF600|162966	-0.815064667	0.379615565	0.031786981
DLD|1738	0.814806097	0.379560297	0.031816331
IRF7|3665	-0.482480757	0.224794239	0.031847321
HMX2|3167	0.281618752	0.131256652	0.031908265
NACA2|342538	-0.857371708	0.399657053	0.031931381
PLGLA|285189	-0.416063695	0.193986177	0.031967958
LOC283392|283392	0.318512898	0.148515374	0.031981164
FOXC2|2303	0.325491331	0.151795547	0.032010894
GTPBP8|29083	1.04001219	0.485130827	0.032050779
PNO1|56902	1.081626855	0.504632321	0.032081321
WIBG|84305	-0.984683867	0.459449108	0.032098346
SLC36A2|153201	-0.476160815	0.222217975	0.032132003
OXSR1|9943	1.331091165	0.621204765	0.032132576
FANCE|2178	0.751822546	0.350959814	0.032178209
CEP72|55722	0.61811828	0.288567771	0.032191777
ZNF524|147807	-0.847325654	0.395601194	0.032204082
ATXN7L2|127002	-0.904077485	0.422130873	0.03221767
APOL2|23780	-0.60211974	0.281238732	0.032277589
RAB7A|7879	1.734885311	0.810728362	0.032362083
MYOM2|9172	-0.505095656	0.236050081	0.032372409
MRPS23|51649	1.193700912	0.557945023	0.032398453
CABIN1|23523	-1.282012935	0.599263612	0.03241026
VPS37B|79720	-0.94754987	0.44303921	0.032455965
CD2BP2|10421	-1.570914242	0.734749163	0.032514475
C10orf107|219621	-0.237669237	0.111167949	0.032522466
PHF13|148479	1.193328069	0.558219455	0.032537849
SLC39A14|23516	0.614902329	0.287670437	0.032555434
PPP2R2A|5520	1.013361144	0.474120978	0.032569763
KLK4|9622	-0.220073985	0.10308558	0.03277189
TRIM74|378108	-0.516934735	0.242161207	0.032787816
FURIN|5045	-0.765871911	0.358791671	0.032794789
FANK1|92565	-0.346643883	0.16239404	0.032794923
STAP2|55620	-0.536778366	0.251473191	0.032798999
SYTL1|84958	-0.497440109	0.233063656	0.032813898
GHITM|27069	0.85568796	0.400952059	0.032831295
UHRF2|115426	1.034466021	0.484957923	0.032916139
DPY19L2|283417	-0.253846676	0.11900959	0.032925264
DFFA|1676	1.220812318	0.572372538	0.032933084
FBXO22|26263	1.068272748	0.500904632	0.032950419
AP1G2|8906	-0.703694374	0.329997775	0.032972206
AKR7A2|8574	0.916914579	0.430119321	0.033025928
CCDC92|80212	-0.88301763	0.414402459	0.033103858
COL22A1|169044	0.313653659	0.147277246	0.033197899
SDHA|6389	0.817944152	0.384081996	0.033203977
E2F5|1875	0.623073191	0.292619624	0.033229949
CD160|11126	-0.494558058	0.232288434	0.033248568
GARS|2617	1.058310485	0.497173851	0.033283001
LEKR1|389170	-0.506132025	0.237806601	0.033309348
MAGIX|79917	-0.353136564	0.165947216	0.03333664
WASL|8976	1.168713362	0.549227609	0.033343574

C20orf202|400831	0.517929308	0.243400129	0.033345998
SPIRE1|56907	0.383038291	0.180021195	0.033358649
RTDR1|27156	-0.326752293	0.153602269	0.033398308
KIF13A|63971	0.667262795	0.313725348	0.033428299
PIGC|5279	-1.046230156	0.492011401	0.033467074
PFKFB1|5207	-0.594417048	0.279550272	0.033475518
COQ4|51117	-0.898647833	0.422696047	0.03350404
DPP8|54878	0.686623659	0.323029743	0.033538737
PTGIS|5740	0.237218964	0.111620057	0.033566925
PUS1|80324	-0.920168874	0.433001043	0.033578586
CSE1L|1434	0.987563293	0.464773425	0.03360102
C12orf10|60314	-1.172000421	0.551845091	0.033688112
GTF2IP1|2970	-1.034117796	0.48694344	0.033695923
CDK8|1024	0.634316607	0.298689165	0.033697919
PCDHGA6|56109	0.33403839	0.157335648	0.033745965
FANCF|2188	-0.988982015	0.46611594	0.033858644
LMTK2|22853	0.454312393	0.21415212	0.033884196
ANKDD1A|348094	-0.587762119	0.277116815	0.033922606
BHLHE22|27319	-0.389950428	0.183883901	0.033952626
MMAA|166785	-1.007858797	0.475334545	0.033979519
NCRNA00105|80161	-0.507979486	0.239615216	0.034007725
PMM2|5373	-1.019894703	0.481086985	0.034007747
LYSMD4|145748	-0.749488574	0.353592216	0.034036328
C9orf41|138199	0.533755272	0.251886367	0.034087763
ERCC3|2071	1.591335735	0.751035959	0.034102786
C20orf3|57136	1.031497707	0.486975052	0.034160367
SRGAP3|9901	-0.613425281	0.289612308	0.034167344
STAG3L4|64940	0.962117862	0.454273067	0.034181126
SPAG9|9043	1.035207332	0.488793745	0.034185087
RASGEF1C|255426	-0.279469068	0.131963092	0.03419353
LOC100302640|100302640	-0.404023537	0.190885358	0.034295773
FTCD|10841	-0.286487405	0.135359416	0.034302807
PCGF3|10336	-1.247257344	0.589463489	0.034351647
SLC38A10|124565	-0.820678757	0.387985686	0.034410394
PAOX|196743	-0.730991825	0.345725472	0.034483614
SULT1A3|6818	-0.715179442	0.338253621	0.034487177
ZIC2|7546	0.160608934	0.075963005	0.034489257
NCRNA00219|114915	-0.699039866	0.330662098	0.034510052
RPUSD3|285367	1.200673902	0.567951004	0.034511436
UBE4B|10277	1.305395898	0.61756418	0.034533912
PKD1L1|168507	-0.344272491	0.162913529	0.034581752
HSPA8|3312	0.520449537	0.246284542	0.034583291
SMS|6611	0.656549772	0.310712429	0.034596798
LOC728855|728855	-0.664076069	0.314314533	0.034619981
CREG2|200407	0.384522693	0.182039629	0.034660544
B4GALT5|9334	0.753930575	0.35692602	0.034661748
ZMAT5|55954	-0.802031387	0.379734241	0.034679071
MRPL45|84311	0.987623276	0.467636459	0.034691021
TRIM11|81559	-0.81777145	0.387355349	0.034758045
THRAP3|9967	1.370895049	0.649365279	0.03476107
OR7E91P|79315	-0.276801246	0.131129214	0.034780522
PGM2|55276	0.705859578	0.334400469	0.034787689
EFCAB4A|283229	-0.365176888	0.173007703	0.03479337
TECR|9524	-0.875179072	0.414758962	0.034850535
GSR|2936	0.487443965	0.231033587	0.034872117
NPTN|27020	1.04077732	0.493424053	0.034919051
FN3KRP|79672	1.338104455	0.634429991	0.03493212
HTRA2|27429	1.392468076	0.660220795	0.034936422
MSX1|4487	-0.212052856	0.100547732	0.034946565

FDXACB1|91893	-0.789539071	0.374411314	0.034966348
SNX4|8723	0.945937701	0.448600378	0.034975475
AKAP3|10566	-0.438464713	0.207958827	0.034994608
C21orf125|284836	-0.345478497	0.163912902	0.035057331
ECHDC2|55268	-0.494058198	0.234419325	0.03506707
PCDHGB2|56103	0.314599552	0.149275563	0.035073506
CCT7|10574	1.37054437	0.65043308	0.035106671
CAPS2|84698	-0.407783392	0.193560339	0.035139189
AGAP7|653268	-0.48455574	0.23002961	0.035161559
AIG1|51390	-0.992214889	0.471064039	0.03517596
NAP1L5|266812	-0.602717509	0.286189686	0.035203736
REP15|387849	-0.527833965	0.250666951	0.035228837
FAM53B|9679	-0.810269346	0.384795082	0.035228987
TTLL3|26140	-0.591397566	0.281077703	0.035375319
CDC123|8872	1.589533179	0.755486502	0.03537965
TSNAXIP1|55815	-0.346693824	0.164789965	0.035391292
NXN|64359	0.661053642	0.314296364	0.035441194
DUSP23|54935	-0.447995264	0.213019833	0.035459797
ASAP1|50807	0.649847741	0.309051604	0.035490606
NUCB2|4925	-0.651707238	0.309951985	0.035500131
ZHX1|11244	0.756077775	0.359615807	0.035513018
VWA1|64856	-0.433415387	0.20619728	0.035558075
RHD|6007	-0.459276349	0.218534828	0.035586904
FLJ10661|286042	-0.493607685	0.234877569	0.035592441
PTK2|5747	1.355903079	0.645529965	0.03568932
RPN2|6185	0.949707499	0.452150517	0.035691618
ZACN|353174	-0.376689687	0.179341072	0.035692837
EMR4P|326342	-0.383511897	0.182631502	0.035735709
GINS4|84296	0.64215966	0.305896818	0.035793208
TMCO4|255104	-0.644504612	0.307028697	0.035802152
SELO|83642	-0.82016636	0.390790361	0.035840053
FZD7|8324	0.315356241	0.150269747	0.035852089
PGA5|5222	-0.312921327	0.149165582	0.035921769
USF1|7391	-1.014256796	0.483619634	0.0359741
FLJ45340|402483	-0.759804306	0.362303499	0.035980416
ZNF205|7755	-0.774417018	0.369293533	0.035991543
ILF2|3608	1.028277095	0.490407239	0.036012874
RNF180|285671	-0.342354629	0.163315182	0.036057208
UQCRFS1|7386	0.836005964	0.398861388	0.03608386
C17orf46|124783	-0.590132615	0.28157288	0.0360961
ITIH4|3700	-0.377107029	0.179937337	0.036102719
KPNB1|3837	1.107876204	0.528663373	0.036116137
PPAT|5471	0.675408494	0.322328774	0.036135235
MCM7|4176	0.79146048	0.377726693	0.036142042
C16orf79|283870	-0.469099899	0.223944957	0.036196717
TSKS|60385	-0.34094619	0.162798867	0.036235166
ARSD|414	-0.627093102	0.299504875	0.036280837
FBXL6|26233	-0.845530356	0.403834759	0.036281963
COPS5|10987	1.691154971	0.807804461	0.036302741
SLC15A2|6565	-0.348681074	0.166553959	0.036304351
MLXIPL|51085	-0.279541197	0.133535488	0.036314794
ZNF438|220929	-1.022559204	0.488623983	0.036373056
PRRT1|80863	-0.583063938	0.278711898	0.03643895
AGPAT2|10555	-0.544205337	0.260186761	0.036474757
LIME1|54923	-0.427180563	0.204274757	0.036509617
PVR|5817	1.155493017	0.552553406	0.03651122
EFEMP2|30008	-0.432215126	0.206710784	0.03653549
LGI2|55203	0.355755873	0.170144293	0.036536413
PHLDB3|653583	-0.769461942	0.368091988	0.036581286

GGT1|2678	-0.284742197	0.136235484	0.036611183
ODF3B|440836	-0.291824479	0.139627254	0.036615536
VWA5A|4013	-0.399762943	0.191280912	0.036624452
AQP8|343	-0.434798069	0.20809033	0.036665623
SLC5A11|115584	-0.346471093	0.165828625	0.036677826
STXBP5L|9515	0.319849656	0.1531489	0.036753828
RPLP0P2|113157	-0.495097436	0.23706138	0.036754753
GLYCTK|132158	-0.360604362	0.172683266	0.036776055
GOLGA7|51125	1.00143391	0.479683858	0.036825296
POM121L8P|29797	-0.361671573	0.173241524	0.036827384
C6orf163|206412	-0.438618797	0.210101893	0.036829598
USHBP1|83878	-0.401309275	0.192241894	0.036840931
CEACAM21|90273	-0.223832846	0.107230251	0.036851639
EIF2C2|27161	0.755333336	0.362060979	0.03696008
NUP210L|91181	-0.383571149	0.183932224	0.037033555
RPS15|6209	-0.786183162	0.377053774	0.037063031
NCOA6|23054	1.324959151	0.635454204	0.037063996
CDKN3|1033	0.558987506	0.268096794	0.037067374
EYA3|2140	0.459412795	0.220354143	0.037079845
C1orf163|65260	0.957281564	0.459168712	0.037086147
ITFG3|83986	-1.014347289	0.486630737	0.037121149
IQSEC2|23096	-0.867580174	0.416321723	0.037167661
SLC1A2|6506	-0.34558686	0.165854015	0.037189193
CHCHD2|51142	1.315607743	0.631467539	0.037213585
PIK3R4|30849	1.00941326	0.484563311	0.037238456
TAF13|6884	0.576651703	0.276823777	0.037242079
ID2B|84099	-0.397010775	0.190651549	0.037306911
MCM8|84515	0.63417937	0.304562557	0.037318445
NACAP1|83955	-0.913782971	0.43885736	0.037325417
ABHD2|11057	0.518760679	0.24917929	0.037353689
DENND4A|10260	0.83131707	0.399338309	0.037366559
NPEPPS|9520	1.004222875	0.482466124	0.037393919
RSPH10B2|728194	-0.247223083	0.118808446	0.037447231
OSBPL5|114879	-0.638235208	0.306742307	0.037462422
SCUBE2|57758	-0.286574798	0.137740567	0.037476005
ZNF668|79759	-1.012777587	0.486804317	0.037483233
RSPRY1|89970	0.535777831	0.257689844	0.037602855
TMEM37|140738	-0.343145582	0.165057576	0.037622429
ATG7|10533	-1.229971358	0.59174242	0.03765793
?|388795	-0.328874248	0.158235656	0.037674126
DYRK2|8445	0.779496929	0.37519986	0.037750713
ALPK3|57538	-0.292012556	0.140599538	0.037809887
GSG2|83903	0.443244665	0.213489462	0.037876391
KIAA0467|23334	-0.948154566	0.456700972	0.037885173
NPEPL1|79716	-0.848152216	0.408831919	0.038026001
REXO2|25996	0.955024184	0.460401291	0.038048679
PDP2|57546	0.60484895	0.291746346	0.038153535
KIF12|113220	-0.208235023	0.100478123	0.038224294
MTCH1|23787	0.998562816	0.481835174	0.038226683
SF3A2|8175	-0.893677707	0.431283426	0.038252804
DCI|1632	-0.71349391	0.344445942	0.038319103
TGM1|7051	-0.39770681	0.192048737	0.03837151
ROCK1|6093	0.778841615	0.376153464	0.0384016
ZCWPW1|55063	-0.598032207	0.288906813	0.038453931
VOPP1|81552	-1.073288679	0.518617517	0.038497433
ID4|3400	-0.474092817	0.229107062	0.0385173
APOBEC3F|200316	-0.589789891	0.285042661	0.038534031
TOP1|7150	0.872534654	0.421762323	0.03856647
CELF1|10658	0.986478112	0.476843987	0.038568152

MDH1|4190	1.364062763	0.659467838	0.038599649
THBS1|7057	0.422786214	0.204468511	0.038665171
CASP4|837	-0.624718655	0.302236985	0.038735773
DNMBP|23268	-0.571715865	0.276623094	0.038755984
TYMS|7298	0.488636392	0.236448831	0.038775356
CPLX1|10815	-0.421638202	0.204052789	0.038798347
MTIF3|219402	-0.960496428	0.46486257	0.03881013
DNAJC15|29103	-0.345507913	0.167245542	0.03884055
RPL10|6134	-1.00345375	0.485825877	0.038879529
TGOLN2|10618	0.892231478	0.432128148	0.038947782
MED12L|116931	0.296603529	0.143667933	0.038969622
UFC1|51506	-0.756503932	0.366547555	0.039030709
SH3TC1|54436	-0.561497294	0.272217263	0.039142975
SPTLC2|9517	0.853604916	0.413964365	0.039205345
PPIL2|23759	-1.295747558	0.628387175	0.039205736
SARS|6301	1.217140684	0.590314796	0.039221999
PTPN6|5777	-0.755494851	0.366544308	0.0392908
NID2|22795	0.430908863	0.209108995	0.039332602
PKHD1|5314	-0.206489269	0.100213114	0.039350624
SLC25A21|89874	0.346189854	0.168032088	0.039373655
ODF2L|57489	-0.639046774	0.31022713	0.039405048
B3GNTL1|146712	-0.671609969	0.326093301	0.039440257
PLA2G4C|8605	-0.445300128	0.216215556	0.039444511
TCEB2|6923	-0.711816605	0.34563011	0.039448748
IKBKE|9641	-0.656965323	0.319059182	0.039487493
SSR4|6748	-0.627861972	0.304930358	0.039490982
EFTUD2|9343	1.200776419	0.583235777	0.03951166
SERINC3|10955	0.852330384	0.414105957	0.039566883
PPP1R1B|84152	-0.228630607	0.111084248	0.039573524
PRKCG|5582	-0.198752064	0.096570763	0.039580757
EPS8L1|54869	-0.379803433	0.184556212	0.039597026
ZDHHC24|254359	-0.760531473	0.369565511	0.039599056
TREX1|11277	-0.651712242	0.316705883	0.03961089
SLC7A9|11136	-0.391123822	0.190087486	0.039628642
RPL34|6164	-0.708523658	0.34437899	0.039648233
FAM59A|64762	0.513577772	0.249630452	0.039652355
TRPV6|55503	-0.221496561	0.107664124	0.039658129
GGT6|124975	-0.244674721	0.118996228	0.039767552
GLRX5|51218	1.40412598	0.682966379	0.039789982
HLA-DRB1|3123	-0.318353499	0.154872221	0.039822233
LOC400891|400891	-0.24038832	0.11696612	0.039860063
KLHL8|57563	0.692961549	0.33722177	0.039887366
ZNF276|92822	-0.9025324	0.439234213	0.039899659
C4BPA|722	-0.136364206	0.066371513	0.039921593
CRADD|8738	0.787324289	0.383225428	0.03993044
FUT10|84750	0.793796499	0.386399505	0.039942661
GAL3ST2|64090	-0.33349628	0.162413737	0.040036238
C16orf48|84080	-0.566664969	0.275967904	0.040036442
ERP27|121506	-0.254026645	0.123719941	0.040049374
TIMM8A|1678	1.008425582	0.49115899	0.040057538
LRRC3|81543	0.371184382	0.180809376	0.040081841
UMODL1|89766	-0.247590595	0.120621681	0.04010935
KIF23|9493	0.546164579	0.266090794	0.040116271
CCL3|6348	-0.375048906	0.182737255	0.040131355
FGFR3|2261	-0.290695025	0.141659957	0.04016363
SMARCC1|6599	0.967726032	0.471610481	0.040173511
LSG1|55341	1.219234845	0.594217582	0.04018596
RASSF3|283349	0.484530655	0.23617205	0.04020852
PSMB10|5699	-0.508929068	0.248126108	0.040258148

KHSRP|8570	1.202550379	0.586305178	0.040260559
PMS2CL|441194	-0.885896322	0.431973006	0.040285056
SYT1|6857	0.241988031	0.118050557	0.040377337
MRPL23|6150	-0.664985977	0.324476074	0.040421599
GSTM2|2946	-0.294076753	0.143520364	0.040459712
KIAA0146|23514	0.865926224	0.422649297	0.040481133
HDHD3|81932	-0.67439496	0.329229101	0.040520206
CEBPZ|10153	0.877360893	0.428426818	0.04057309
DUOX2|50506	-0.247365062	0.120795164	0.040579012
CSMD1|64478	-0.320203774	0.156420765	0.040651514
CCDC86|79080	1.176188592	0.57471694	0.040702008
C1orf97|84791	-0.619253429	0.302602526	0.040714557
PHYH|5264	0.657664591	0.321372689	0.040714731
RG9MTD3|158234	-0.92257933	0.450876503	0.040737607
PIK3CA|5290	0.705639573	0.344901728	0.040764748
BCO2|83875	-0.343219183	0.167853931	0.040879595
MPZL2|10205	-0.551028476	0.269494191	0.040886749
PRKRIP1|79706	-0.950999106	0.465123737	0.040892801
C19orf12|83636	-1.246747354	0.609813688	0.040906885
OPN3|23596	-0.537446426	0.262932211	0.040948668
CHST10|9486	-0.586695671	0.287031454	0.040952384
PIGS|94005	0.604929606	0.295966201	0.040961996
ZNF3|7551	-1.136606824	0.556121585	0.040972327
P2RY1|5028	0.388588916	0.190151711	0.040995742
HLX|3142	-0.582043104	0.284838119	0.041011159
LOC100128288|100128288	-0.464082382	0.227116709	0.041016299
LRP8|7804	0.505104844	0.247201456	0.041023507
FBXW11|23291	1.039119158	0.508563501	0.041028322
ALG14|199857	-1.073623481	0.525581348	0.041078666
ZNF213|7760	-0.79580708	0.389713383	0.041148388
LOC440896|440896	-0.381478308	0.186818589	0.04115432
GOLGA8DP|100132979	-0.365996669	0.179256502	0.041176499
FUCA1|2517	-0.673466479	0.329860321	0.041184109
BECN1|8678	1.142823705	0.559848809	0.041220249
SF3A1|10291	0.945438766	0.463184367	0.041233776
TDGF1|6997	-0.244636076	0.119908477	0.041331405
PPP3R1|5534	1.001092103	0.490811595	0.041383526
PHAX|51808	1.300596635	0.637880145	0.04145644
TUBGCP4|27229	0.748048093	0.36691696	0.041476035
WDFY2|115825	-0.96090908	0.471423866	0.041518707
KPNA1|3836	1.368377031	0.671424843	0.041547967
HSPA9|3313	1.27615601	0.626188181	0.041552409
SAMD1|90378	0.777625634	0.38161081	0.041575411
MYLPF|29895	-0.39007728	0.191475393	0.041628116
FPR1|2357	0.327561787	0.160792913	0.041633453
STT3B|201595	0.553327787	0.271662098	0.041667717
AQP6|363	-0.254001552	0.124714016	0.041682916
DLGAP5|9787	0.447782959	0.219911008	0.041730201
DHX9|1660	0.956396078	0.469754618	0.041755555
ADAM9|8754	0.616764247	0.303068751	0.041844453
COMMD1|150684	-1.08341928	0.532585162	0.041924898
?|155060	-0.553551864	0.272183792	0.041977521
THAP3|90326	-0.8038649	0.395313427	0.042003298
LOC100132287|100132287	-0.44303371	0.217899698	0.042032301
PLLP|51090	-0.414049492	0.203710083	0.042098721
SRA1|10011	-1.170881456	0.576227493	0.042155907
ECM1|1893	-0.414409593	0.203988064	0.042200405
TCTA|6988	0.905877785	0.445946368	0.04221845
RECQL|5965	0.701435517	0.345365161	0.042255325

C16orf75|116028	0.646346756	0.318255149	0.042264383
DPP7|29952	-0.498720874	0.245600499	0.042293728
LRRC36|55282	-0.304777549	0.150117149	0.042329641
SEPT10|151011	0.629150828	0.309953259	0.042374346
TMEM38A|79041	0.412717141	0.203331604	0.042379548
GSC|145258	-0.234231138	0.115423957	0.042426594
ADAM10|102	0.520687391	0.256595423	0.042436353
SATB1|6304	0.394738688	0.194587246	0.042499572
ZNF266|10781	-0.673722412	0.332244204	0.042581397
NPFF|8620	-0.378570248	0.186741331	0.042637463
POLR2D|5433	1.117354684	0.551262957	0.042672678
SLC4A1AP|22950	1.150494607	0.567831747	0.042752607
MRPS7|51081	1.467953076	0.724518169	0.042753575
NR2E3|10002	-0.30293305	0.149535224	0.042782135
SERPINA4|5267	-0.157398741	0.077718028	0.042841298
MRPL36|64979	1.161365722	0.573443087	0.04284168
ARL16|339231	-0.917043299	0.452958557	0.04291222
LHPP|64077	-0.540606751	0.267024984	0.042913085
PSMB2|5690	1.367613116	0.675793344	0.042999486
RNF168|165918	0.430648689	0.212836566	0.043034256
SNORD89|692205	-0.504214245	0.249254404	0.043084488
SMARCA5|8467	0.830591007	0.410645498	0.043109524
TYK2|7297	-1.121454892	0.554524792	0.043138032
PACSIN2|11252	0.53774039	0.265947788	0.043178698
STRADA|92335	-1.480967731	0.732453293	0.043183803
KMO|8564	-0.321523593	0.159059825	0.043238334
COX4I1|1327	-0.889455789	0.440044868	0.043250342
FCRL2|79368	-0.25511832	0.126227866	0.043270086
B3GALT6|126792	-1.040656326	0.515030061	0.043323888
SSR2|6746	-0.88052079	0.435855075	0.043361132
ZNF167|55888	-0.418824828	0.207341012	0.043385397
CTNND1|1500	1.094082879	0.541642284	0.043390002
GDF6|392255	0.335143278	0.16592149	0.043394724
ACACA|31	0.955428573	0.473047295	0.043411235
ISM1|140862	-0.30719307	0.152108388	0.043428328
NT5E|4907	-0.285634983	0.141469021	0.043480585
FPR2|2358	0.327112913	0.162066364	0.043550799
STK36|27148	-0.842214118	0.417300983	0.043566127
KCNK6|9424	-0.380281688	0.18846516	0.043613926
TH1L|51497	1.427074659	0.707841264	0.043790128
ERCC5|2073	-1.072258881	0.531919255	0.043817738
CES8|283848	-0.2533711	0.125691043	0.043818318
CWH43|80157	-0.199975486	0.099209235	0.043831955
LCAT|3931	-0.507859069	0.252110623	0.043964463
OCRL|4952	0.850603637	0.422295927	0.043984767
ACOT7|11332	0.583747516	0.289831842	0.044000048
GALNT12|79695	-0.373072708	0.185243155	0.044013505
MACF1|23499	0.90180993	0.447817223	0.044031566
C15orf57|90416	-0.941469185	0.467513501	0.044032671
PSME3|10197	1.441057429	0.715638183	0.044044443
GLI4|2738	-0.580725352	0.28849775	0.044122148
LENG9|94059	-0.514709141	0.255716507	0.044134458
MCM10|55388	0.437893112	0.217553944	0.044135416
C18orf55|29090	0.893102824	0.443748069	0.044153059
SNX2|6643	0.85456536	0.424892632	0.044299044
ABCA2|20	-0.517129467	0.257157329	0.044331175
LIPT1|51601	-0.939840807	0.467404504	0.044350242
SFT2D3|84826	-0.959604186	0.477394335	0.044421973
EMP1|2012	0.404745962	0.201379155	0.044444929

RPS11|6205	-0.895868376	0.445750154	0.044452428
DHX35|60625	1.2384969	0.616331177	0.044487676
MPST|4357	-0.632939661	0.314991004	0.044495828
GLS|2744	0.518741175	0.258205376	0.044534434
LACTB2|51110	0.656368369	0.326806095	0.044597227
ODC1|4953	0.599113224	0.298314263	0.044608329
SLC1A5|6510	0.370609229	0.18455335	0.044628265
MUC12|10071	-0.273334136	0.136115358	0.044631975
PHKG1|5260	-0.453451751	0.225847583	0.044666915
PRR14|78994	-1.268537026	0.631881679	0.044690634
C10orf12|26148	0.672633604	0.335343042	0.044876786
CSNK1G1|53944	0.895346453	0.446431621	0.044902962
POLN|353497	-0.420687102	0.209833223	0.044977574
FITM2|128486	0.616058355	0.307400275	0.045060233
C1orf93|127281	-0.616440134	0.307690911	0.045130132
C4BPB|725	-0.178693858	0.089213203	0.045177514
CBX1|10951	0.757222939	0.378066047	0.045189598
STAT6|6778	-0.860990501	0.429895151	0.045199636
CDH13|1012	0.391541017	0.195591538	0.045303014
ECT2|1894	0.441401261	0.220508469	0.045312391
NPIP|9284	-0.630854612	0.315213581	0.045354032
TNNI1|7135	-0.344963447	0.172380033	0.045373013
UBXN7|26043	0.638148507	0.318918726	0.045395047
DGCR6L|85359	-0.855415906	0.427531681	0.045411295
ASCC1|51008	1.061700428	0.530866	0.045506686
THAP7|80764	-0.776049804	0.388112839	0.045549219
CCDC61|729440	-0.624373053	0.312276885	0.04556279
UBXN2B|137886	0.834518106	0.417423135	0.045585222
TIGD6|81789	-1.232051383	0.616275628	0.045587921
SLC12A3|6559	-0.234281328	0.117191633	0.045594271
SKAP1|8631	-0.295506354	0.147822356	0.045601427
KRAS|3845	0.78367745	0.392120043	0.045655425
LOC84931|84931	-0.274962073	0.137612147	0.045706416
KIAA1009|22832	-0.63115261	0.315878664	0.04570738
C9orf130|100128782	-0.571621626	0.28608719	0.045709301
MRPL42P5|359821	-0.439282091	0.219874881	0.045730429
GAB2|9846	0.628146766	0.314409327	0.045731513
C1QTNF3|114899	-0.537427122	0.269012003	0.045740387
LTBR|4055	-0.762982987	0.381934795	0.04575151
FAM21A|387680	0.241915027	0.121101589	0.045757811
CNOT7|29883	1.013800282	0.507620267	0.045807508
RPS14|6208	-0.930482573	0.465948798	0.045829188
LOC440461|440461	-0.273678252	0.137072113	0.045868597
WFIKKN1|117166	-0.354077234	0.177345874	0.045875726
C1orf63|57035	-0.556951771	0.278967762	0.045882397
ATP6V1B2|526	1.038602925	0.520385929	0.045952205
C15orf56|644809	-0.531238059	0.26618671	0.045962806
SGK2|10110	-0.242687786	0.121621214	0.045994957
FAM13AOS|285512	-0.461941007	0.23151644	0.046011933
VHL|7428	0.724699748	0.363234603	0.046028855
RYK|6259	1.180644142	0.591885086	0.046073588
AGAP8|728404	-0.457343077	0.229341302	0.046134655
SURF4|6836	1.155613367	0.57959045	0.046169025
OGFR|11054	-0.895298785	0.449044941	0.046175624
TRAPPC5|126003	-0.519151988	0.260385988	0.046176265
IFT140|9742	-0.745662949	0.374005144	0.046182249
RPS6|6194	-0.787311349	0.394903361	0.046186921
MRPL3|11222	1.142311183	0.573028422	0.046210728
CYP51A1|1595	0.754643073	0.378569532	0.046216922

RAB9A|9367	0.887061621	0.445031912	0.046233645
FLJ11235|54508	-0.482915704	0.24229001	0.046247116
MUC2|4583	-0.193338673	0.0970066	0.046256346
RRP7B|91695	-0.687794795	0.345240476	0.046346984
PROS1|5627	0.439758287	0.220767285	0.0463761
PER1|5187	0.659054229	0.330867523	0.046382293
PAK1IP1|55003	0.78473238	0.393991812	0.046398721
LOC154822|154822	-0.269351148	0.135242295	0.046412859
HNF1B|6928	-0.178489677	0.08962741	0.046430002
HIGD1A|25994	0.759696975	0.381500551	0.046443616
CAMK1G|57172	-0.42183296	0.211855934	0.046466466
MFN1|55669	0.724326381	0.363855063	0.046513846
CD1C|911	-0.287951681	0.14470149	0.046594196
SAMD11|148398	-0.272109495	0.13674137	0.046595633
SLC46A1|113235	-0.77448107	0.389196204	0.04659642
EAF1|85403	0.92677961	0.465832758	0.046644778
RAB4B|53916	-0.754549371	0.379296139	0.046663574
U2AF1|7307	-1.15631079	0.581391156	0.046715675
SGK494|124923	-0.60354603	0.303508394	0.046749289
SIAE|54414	-0.587873472	0.29569625	0.046800722
AMAC1L2|83650	-0.392002463	0.197202453	0.046831891
SNIP1|79753	1.070335113	0.53865122	0.046915168
ZNF264|9422	-1.142104158	0.574815881	0.046933001
USP39|10713	1.424177631	0.716817017	0.046943701
C1orf216|127703	0.778500103	0.391885874	0.046972508
DNHD1|144132	-0.490908577	0.247122627	0.046978067
CCT2|10576	0.969987681	0.488293087	0.046979318
ZNF429|353088	-0.481336096	0.242335109	0.047006494
RAB2A|5862	1.117272358	0.562918313	0.047168291
NT5C1B|93034	-0.419785371	0.211506173	0.047173085
BLOC1S1|2647	-0.714059462	0.359980098	0.047299539
TRIM38|10475	-0.810784325	0.408762938	0.047310778
AP3M1|26985	1.009003228	0.50870175	0.047313075
FAM160B1|57700	-0.867541266	0.437569141	0.047407865
C5orf56|441108	-0.585925953	0.295552131	0.047425633
KDM4B|23030	-0.826370165	0.416858311	0.047437105
RAC3|5881	0.507904312	0.25622628	0.047451368
NUDT14|256281	-0.550446599	0.277692618	0.047455132
AGBL2|79841	-0.247844771	0.125049978	0.047483076
SRP54|6729	1.007194546	0.508227341	0.04750392
ACSL4|2182	-0.374391268	0.188951124	0.047544352
YIPF6|286451	0.714664656	0.360692146	0.047549777
PCSK4|54760	-0.379954061	0.191763935	0.047550524
MBD3|53615	-0.90667483	0.457692202	0.047594562
KIAA0922|23240	0.788347822	0.397965951	0.047597678
ZNF554|115196	-1.045102966	0.527654311	0.04762959
RABL2B|11158	-0.830628213	0.41947945	0.047687753
ALG2|85365	-1.009702405	0.509965713	0.047710075
DNAJC17|55192	-0.736704674	0.372145168	0.04774665
ZNF444|55311	-0.839332921	0.424046788	0.047777699
CHMP5|51510	0.980831793	0.495536965	0.047778725
PIGQ|9091	-0.83509991	0.421913241	0.047780364
UBR5|51366	1.157971511	0.585093564	0.047802279
HLTF|6596	0.541686372	0.27370528	0.04780629
ST8SIA5|29906	-0.303453447	0.153364292	0.04785602
CHD7|55636	0.7066288	0.357157457	0.04787461
CSRNP1|64651	-0.701560729	0.354601553	0.047878191
ABCD1|215	-0.551449911	0.278789797	0.047927213
KPNA2|3838	0.635216408	0.321208799	0.047975998

LOC349114|349114	-0.70028274	0.354129037	0.04798751
HNRNPR|10236	0.791434046	0.400231535	0.04799186
ANGPTL2|23452	0.576705131	0.291652588	0.047999802
AVPR1A|552	0.298231685	0.150839112	0.048024576
AKAP12|9590	0.303379507	0.153455932	0.048043745
KLF7|8609	0.472678103	0.239141075	0.048090716
MYOC|4653	-0.241017949	0.121943703	0.048101684
NUDT21|11051	0.777276322	0.393305568	0.048124687
SUCLA2|8803	0.705677177	0.357273848	0.048248671
MST1P2|11209	-0.324101413	0.164090459	0.048252412
C7orf31|136895	-0.417322968	0.211301276	0.048266565
P2RX4|5025	-0.67487705	0.341782084	0.048315504
SEC23A|10484	0.737427081	0.373488193	0.04833263
DSCC1|79075	0.558339998	0.282797864	0.048342749
PCNA|5111	0.673577899	0.341225522	0.04838217
TDGF3|6998	-0.253613955	0.12848306	0.048392014
LGALS9|3965	-0.423910403	0.214841496	0.048480646
SLC22A11|55867	-0.297195246	0.150636006	0.04850282
EFCAB6|64800	-0.317253601	0.160821882	0.048529558
LOC441089|441089	0.826486035	0.419005984	0.048553446
TSPYL3|128854	0.324562523	0.164546736	0.048556655
FLJ39653|202020	-0.613618302	0.311110198	0.048569627
NAA35|60560	0.841187492	0.426494339	0.04857197
PCDHGB3|56102	0.339415674	0.172108964	0.048598466
PRPF31|26121	-1.201176209	0.609208978	0.048644076
DPY19L2P4|442523	-0.294648147	0.149441719	0.048648519
EXOC6|54536	0.816026618	0.413945209	0.048685035
METAP2|10988	1.074446602	0.545123484	0.048722152
POLR2C|5432	1.320261941	0.669895718	0.048741383
FCF1|51077	0.898012019	0.455909271	0.048870869
C12orf45|121053	-0.699360658	0.355094248	0.048894883
CYP3A5|1577	-0.20071805	0.101918862	0.048908253
WDR27|253769	-0.60480816	0.307139273	0.048934052
DCAF12L2|340578	0.290774806	0.147677641	0.048955011
PSENEN|55851	-0.614032151	0.311886696	0.048979884
SLC30A6|55676	0.621956298	0.315913841	0.048981471
APOBEC3A|200315	-0.398241273	0.202367981	0.049078792
CES3|23491	-0.179694717	0.091332972	0.049129259
LOC100133161|100133161	-0.389547849	0.197997007	0.049132148
ASRGL1|80150	-0.309006239	0.157062642	0.049136237
TEX261|113419	1.369983071	0.696428376	0.04916526
INPP5J|27124	-0.560729854	0.285087699	0.049198438
SLC26A8|116369	-0.400810106	0.203887991	0.049317697
CXCL3|2921	-0.226949198	0.115457554	0.049338998
OTUD6A|139562	-0.400529064	0.203764974	0.049339938
C10orf32|119032	-0.736855797	0.374942879	0.049385524
SLC19A1|6573	-0.79295452	0.403541574	0.049415578
ELAVL1|1994	1.715883105	0.873235546	0.049417517
TTLL1|25809	-0.732521056	0.372844802	0.049451183
BCKDHB|594	0.68670816	0.349581149	0.049486723
PPIB|5479	-0.65566172	0.333839003	0.049529426
NCRNA00201|284702	-0.539092267	0.274498191	0.049539464
SPIN2B|474343	0.907117574	0.461892545	0.049539981
SALL2|6297	0.36224548	0.184492828	0.049591998
RRM2B|50484	0.612091308	0.311749558	0.049598885
GLTSCR1|29998	-1.156154925	0.589073426	0.049684889
ABCA7|10347	-0.428311844	0.218334344	0.049794561
RAB3GAP1|22930	1.116766169	0.569297134	0.049802337
CDC40|51362	0.795915085	0.40575292	0.049811899

RBBP4|5928	0.76317294	0.389117048	0.049844734
BTBD1|53339	0.963295117	0.491318117	0.049921706
C6orf120|387263	0.96343119	0.491399036	0.049927071
HNRNPM|4670	0.880324753	0.449151062	0.049998738

S2. Limit Transcript-Selection with Random Re-Sampling in Training-1.

Table S2 provides a summary of results of random re-sampling to limit the selection of transcripts associated with nodal and distant metastasis. Random re-sampling in R was used to generate the 100 random subsets of Training-1 with 60 of the 75 cases. Univariate logistic regression modeling was performed using R with transcripts included as the explanatory variable. A total of 311 transcripts (GeneSymbol|ID) were identified in at least 80% of the 100 logistic regression models with a $p<0.05$. The seven transcripts in the final MS7 classifier are bolded and highlighted in blue.

**Table S2.**		
**GeneSymbol|ID**	**Percent of the 100 Training-1 Models with 60 of the 75 cases with p<0.05**	**Max P-value in the Training-1 Models that passed with p-value <0.05**
C12orf60|144608	100	0.028415817
C14orf93|60686	100	0.021402299
C22orf43|51233	100	0.039154845
C6orf164|63914	100	0.026851375
C6orf70|55780	100	0.046313669
CALB1|793	100	0.03979270
CCDC130|81576	100	0.040641407
CDRT4|284040	100	0.033133363
COL28A1|340267	100	0.04011027
CUBN|8029	100	0.041236836
DDX24|57062	100	0.037308095
EIF2S2|8894	100	0.033840741
EPR1|8475	100	0.043403939
ETF1|2107	100	0.037956637
ETS2|2114	100	0.038287876
FAM116B|414918	100	0.037878831
FAM170A|340069	100	0.007149499
FAM53A|152877	100	0.048929526
FGFR2|2263	100	0.023147324
GSK3B|2932	100	0.043612411
H2AFB1|474382	100	0.035163813
HSCB|150274	100	0.043022595
IFT27|11020	100	0.040213212
IMPA2|3613	100	0.047453102
ITIH3|3699	100	0.016785322
KIAA0196|9897	100	0.037668608
KRTAP5-10|387273	100	0.045224516
LOC143188|143188	100	0.043771227
LUC7L|55692	100	0.04062051
MAP2K1|5604	100	0.035756349
MED14|9282	100	0.034047191
NLRP9|338321	100	0.02151933
PCDHGA1|56114	100	0.008516382
PTPLAD1|51495	100	0.036369051
**RSRC1|51319**	**100**	**0.023256427**
SENP3|26168	100	0.025170818
STX19|415117	100	0.036759784
TMEM22|80723	100	0.019904241

TRIM50|135892	100	0.048027923
TRPM4|54795	100	0.031563619
TXLNG|55787	100	0.027087847
WDR64|128025	100	0.032023893
ZFP36L2|678	100	0.029852691
ZNF528|84436	100	0.040486241
ZNF688|146542	100	0.03073481
C12orf27|283460	99	0.03651706
C1orf101|257044	99	0.048499385
C1orf228|339541	99	0.046849965
CDC16|8881	99	0.043765227
CROCC|9696	99	0.033991423
LOC728743|728743	99	0.042641143
PCDHAC2|56134	99	0.032836953
TTC21A|199223	99	0.045930301
WFDC8|90199	99	0.046015336
CCDC57|284001	98	0.038147308
CPT1B|1375	98	0.047794325
DACT2|168002	98	0.044549839
LTB4R2|56413	98	0.04891201
OR2A9P|441295	98	0.047074838
RGP1|9827	98	0.040094663
SMURF1|57154	98	0.049794673
SPINLW1|57119	98	0.048800018
TBC1D3C|414060	98	0.046536832
ACAD11|84129	97	0.041203113
C17orf108|201229	97	0.048747984
C19orf25|148223	97	0.048113762
CCDC88A|55704	97	0.043874386
DLST|1743	97	0.046813234
F2R|2149	97	0.044755002
GFM1|85476	97	0.044359772
GLTSCR2|29997	97	0.046932453
LRP5L|91355	97	0.032118225
LRRC27|80313	97	0.047337511
LRRC42|115353	97	0.043650234
NAA50|80218	97	0.04094403
PAFAH1B1|5048	97	0.039408495
PCDHGA3|56112	97	0.039242731
PRPS1|5631	97	0.044059517
PSMA7|5688	97	0.048920828
QRICH1|54870	97	0.038580589
WASH3P|374666	97	0.048997783
YWHAB|7529	97	0.046519772
C2orf81|388963	96	0.04501821
MRPL13|28998	96	0.049354455
PSMD8|5714	96	0.044403835
TNIP2|79155	96	0.039150764
**BDNFOS|497258**	**95**	**0.049197263**
C1orf152|767846	95	0.047047081
CCDC94|55702	95	0.043685509
CYP2E1|1571	95	0.049280888
KLK2|3817	95	0.048470943
LDLRAD2|401944	95	0.049319106
MRPL49|740	95	0.047849741
RNF135|84282	95	0.049936457
SF3B2|10992	95	0.040441275
UROS|7390	95	0.049501804
VILL|50853	95	0.048705441

C19orf18|147685	94	0.048610544
C2CD2|25966	94	0.036231619
CIRBP|1153	94	0.048548141
DDX21|9188	94	0.049459149
DDX27|55661	94	0.048671155
DNAJA3|9093	94	0.049325381
FOLH1|2346	94	0.048408887
IDUA|3425	94	0.048295577
IL20|50604	94	0.049949821
PCMT1|5110	94	0.048914118
PRIM1|5557	94	0.049812218
RCE1|9986	94	0.049731838
SGCE|8910	94	0.049900943
TEKT3|64518	94	0.046496315
UBQLNL|143630	94	0.049991482
CDK4|1019	93	0.047674922
DHX36|170506	93	0.046568634
DPM1|8813	93	0.04908066
GLOD4|51031	93	0.048741933
HCG2P7|80867	93	0.045235353
LIMS3-LOC440895|100271835	93	0.048453862
**MLLT10|8028**	**93**	**0.047834605**
MST4|51765	93	0.04835652
MZF1|7593	93	0.048207005
NUP155|9631	93	0.048334746
PDE9A|5152	93	0.049378082
**PDLIM3|27295**	**93**	**0.048764438**
PTPN18|26469	93	0.046776971
SKINTL|391037	93	0.045737942
SLC25A4|291	93	0.043508077
SPPL2B|56928	93	0.049047456
TNFSF12-TNFSF13|407977	93	0.048840254
USH2A|7399	93	0.045217579
WASH2P|375260	93	0.048635516
WDYHV1|55093	93	0.044227712
XPO5|57510	93	0.045899847
ZNF140|7699	93	0.047938151
C3orf26|84319	92	0.046671312
FERMT2|10979	92	0.048809722
HEXDC|284004	92	0.044926509
HNF1A|6927	92	0.049397811
HOXD13|3239	92	0.042987585
HSD11B1L|374875	92	0.048795801
LMF1|64788	92	0.048502219
NPDC1|56654	92	0.048365428
PCYOX1|51449	92	0.048735813
PNP|4860	92	0.04797088
PRKDC|5591	92	0.043403441
PRMT5|10419	92	0.049023636
RP1L1|94137	92	0.044585874
SUGT1L1|283507	92	0.04986353
TFRC|7037	92	0.049394118
TMED6|146456	92	0.04566104
WASH5P|375690	92	0.049753161
YME1L1|10730	92	0.043579625
AQP2|359	91	0.048929326
ARFGEF1|10565	91	0.041863538
C4orf44|345222	91	0.047829946
CCDC33|80125	91	0.04487868

CLSPN|63967	91	0.049951776
COL18A1|80781	91	0.049091137
FAM193A|8603	91	0.047949723
FES|2242	91	0.045483038
MGC42105|167359	91	0.045612844
NOXA1|10811	91	0.048396479
TUBB1|81027	91	0.045345612
ARRDC4|91947	90	0.046010258
ASB9|140462	90	0.047355008
CCDC101|112869	90	0.048764217
CROCCL1|84809	90	0.04933711
CYP21A2|1589	90	0.049781076
MRPL33|9553	90	0.047314604
NDUFS1|4719	90	0.049311476
PLS1|5357	90	0.049429357
PPT2|9374	90	0.049505249
SLC22A23|63027	90	0.043156965
TMEM219|124446	90	0.047422815
TXNRD2|10587	90	0.049387014
C4orf31|79625	89	0.049439572
CYP2D6|1565	89	0.049387255
EIF2B3|8891	89	0.048898243
GOLGA8F|100132565	89	0.049089678
PCDHGA2|56113	89	0.049434875
PCOLCE2|26577	89	0.049365388
PYY|5697	89	0.046103174
RHBDD3|25807	89	0.049303451
RPS6KA3|6197	89	0.049521673
SLC5A9|200010	89	0.048158737
SNRNP35|11066	89	0.047707578
TAF2|6873	89	0.046196162
TTC9C|283237	89	0.04716192
WFDC6|140870	89	0.048335609
ZNF446|55663	89	0.047969076
C8orf79|57604	88	0.046432352
CIRH1A|84916	88	0.046689048
CYP2C8|1558	88	0.046842961
EIF4A3|9775	88	0.048504241
FLJ39609|100130417	88	0.048949328
GIYD2|79008	88	0.049838914
GPR113|165082	88	0.048505813
MBTPS2|51360	88	0.049526697
MED30|90390	88	0.047932865
RAB5C|5878	88	0.048444644
RPL13|6137	88	0.048536328
SIGIRR|59307	88	0.045994211
THOC4|10189	88	0.047908263
VAT1|10493	88	0.049208191
ZMAT2|153527	88	0.047833456
ZNF582|147948	88	0.049517343
APOBEC2|10930	87	0.048931104
C9orf84|158401	87	0.047861663
ERAP2|64167	87	0.049582516
FAM114A1|92689	87	0.049824556
HOXB13|10481	87	0.049346261
IDI1|3422	87	0.04918764
KIAA1161|57462	87	0.04757246
LDLR|3949	87	0.049929041
MAN2A1|4124	87	0.048496679

MMGT1|93380	87	0.047390255
MTHFSD|64779	87	0.049306123
NEUROD2|4761	87	0.047731603
RRAGC|64121	87	0.049364072
SLC35E2|728661	87	0.047458713
TMX2|51075	87	0.04998282
WDR61|80349	87	0.049854712
ZNF10|7556	87	0.049527672
**APOL4|80832**	**86**	**0.047591098**
ATP5B|506	86	0.045893261
CHKB-CPT1B|386593	86	0.043638427
F10|2159	86	0.049635196
FAM111A|63901	86	0.049295129
FOXD1|2297	86	0.049500301
GART|2618	86	0.047578308
LRRC48|83450	86	0.048049037
NCRNA00167|440072	86	0.049135979
NPIPL3|23117	86	0.049668343
**TBRG1|84897**	**86**	**0.047327698**
TMEM114|283953	86	0.04843099
GTPBP4|23560	85	0.046015941
HAT1|8520	85	0.048538284
JMJD7-PLA2G4B|8681	85	0.049825898
LOC730101|730101	85	0.048185887
MCM4|4173	85	0.049880177
OCM|654231	85	0.044108146
PDZK1|5174	85	0.049739612
PDZK1P1|728939	85	0.048752096
PSMD1|5707	85	0.049590753
RFC1|5981	85	0.048622416
SMCR5|140771	85	0.049511807
STRAP|11171	85	0.043815938
UBQLN1|29979	85	0.049557676
WDR44|54521	85	0.048835574
ADRBK2|157	84	0.048582667
CCDC48|79825	84	0.048333171
CECR1|51816	84	0.048695769
CORO1C|23603	84	0.046070431
CSNK2A1|1457	84	0.049953179
MYH3|4621	84	0.048298352
NMRAL1|57407	84	0.049718896
PLGLB2|5342	84	0.049107826
SENP2|59343	84	0.049728375
USP9X|8239	84	0.048070527
ZSCAN4|201516	84	0.047597529
CUL2|8453	83	0.048354843
DERL1|79139	83	0.049802503
ELMOD3|84173	83	0.046974692
GMCL1|64395	83	0.049679161
HOXA13|3209	83	0.047251066
RPL32P3|132241	83	0.048654235
SLC22A18|5002	83	0.048600334
UBP1|7342	83	0.047100225
ZNF623|9831	83	0.048322198
ADAM22|53616	82	0.049952445
CALU|813	82	0.046955283
DVL2|1856	82	0.049391202
FGF1|2246	82	0.049527918
GTF2E1|2960	82	0.048377925

P4HB|5034	82	0.048474811
SCD|6319	82	0.049813836
TTC16|158248	82	0.048200692
VKORC1L1|154807	82	0.048711092
WISP1|8840	82	0.049265451
ZFP2|80108	82	0.049958693
**ZNF596|169270**	**82**	**0.047461422**
C20orf134|170487	81	0.049815036
CDK2|1017	81	0.049955221
LOC390595|390595	81	0.049621409
MRPL37|51253	81	0.047447651
NANOG|79923	81	0.046298914
P2RY11|5032	81	0.049457097
RPL18|6141	81	0.049436045
SCAPER|49855	81	0.049372401
SFRS2B|10929	81	0.049851399
UBQLN2|29978	81	0.04982079
VPRBP|9730	81	0.049776182
ZNF862|643641	81	0.04924443
C22orf27|150291	80	0.049581422
C6orf217|100131814	80	0.049057036
DDX46|9879	80	0.049049463
ECT2L|345930	80	0.044041086
FAM193B|54540	80	0.04695361
FLJ42627|645644	80	0.048917703
GSTA1|2938	80	0.047762018
LOC100133612|100133612	80	0.046615172
LOC219347|219347	80	0.049526139
LOC284900|284900	80	0.046422871
MUSTN1|389125	80	0.048856233
NCRNA00174|285908	80	0.049941105
NR1H2|7376	80	0.04992139
RPL28|6158	80	0.047128269
STX18|53407	80	0.048967412
TNFRSF10A|8797	80	0.04974747
TNPO3|23534	80	0.049908346
UCP3|7352	80	0.047684473

S3. Evaluate Candidate Transcripts in Training-2.

Table S3 provides a summary of the evaluation of the candidate transcripts from Training-1 in the Training-2 Cohort using microarray based data. Univariate logistic regression modeling for metastasis was performed in R in the 64 Training-2 cases. The relationship with metastasis was assessed using univariate logistic regression modeling with individual transcripts included as the explanatory variable. There were 33 transcripts that distinguished nodal or distant metastasis from stage I disease in Training-2 with $p<0.05$. These transcripts were detected using the Affymetrix Plus 2.0 probeset indicated below. The seven transcripts in the final MS7 classifier are bolded and highlighted in blue.

Table S3.

		Univariate Logistic Modeling In Training-2 (Affymetrix microarray)		
**GeneSymbol|ID**	**Probeset**	**Coefficient**	**Standard Error**	**P-value**
SCD|6319	211708_s_at	1.348364253	0.436155009	0.001991618
KLK2|3817	209854_s_at	5.12179484	1.688834903	0.002423454
LDLR|3949	214170_x_at	1.877044253	0.660560381	0.004488865
**ZNF596|169270**	**232641_at**	**-1.123630563**	**0.422324916**	**0.007800546**
MRPL37|51253	218887_at	2.509100598	0.959347925	0.008911702
**PDLIM3|27295**	**209621_s_at**	**1.873951228**	**0.71693134**	**0.008952824**
PTPN18|26469	213521_at	1.428678566	0.562959781	0.011155215
COL18A1|80781	209081_s_at	-0.999425929	0.397745913	0.011980283
LRRC27|80313	228886_at	1.283775515	0.511571383	0.012091083
LUC7L|55692	1557066_at	-1.05678706	0.422277411	0.012328841
TFRC|7037	237214_at	4.219036884	1.707523076	0.013479157
SLC35E2|728661	217122_s_at	-1.306519776	0.534137211	0.014443588
ZNF623|9831	206188_at	-1.21157478	0.513568375	0.018317816
ZNF10|7556	229848_at	-1.069343172	0.453498331	0.018374336
UROS|7390	203031_s_at	1.355658541	0.575704785	0.018533638
**APOL4|80832**	**223801_s_at**	**-1.061512979**	**0.467183571**	**0.023077216**
**RSRC1|51319**	**235354_s_at**	**0.84580908**	**0.373311459**	**0.0234702**
CCDC57|284001	1553248_at	1.031469412	0.458255601	0.024394377
HOXB13|10481	230105_at	0.868620843	0.39743493	0.028847267
ZNF140|7699	204523_at	-1.602120944	0.734665352	0.029201965
**MLLT10|8028**	**216503_s_at**	**1.331562976**	**0.611733947**	**0.029502752**
SFRS2B|10929	229471_s_at	-3.262596536	1.507603087	0.030457047
PTPLAD1|51495	217777_s_at	0.773490471	0.358247303	0.030842687
HOXD13|3239	207398_at	2.901486206	1.357382088	0.032552438
CCDC88A|55704	1562648_at	-3.867982115	1.830537722	0.034598154
**TBRG1|84897**	**230681_at**	**-0.962005274**	**0.468101902**	**0.039867479**
TAF2|6873	209523_at	-1.473791135	0.717972467	0.040100459
PCDHGA3|56112	211879_x_at	-1.637388203	0.798806994	0.040384731
ZFP36L2|678	201368_at	-0.57804217	0.287994277	0.044735743
**BDNFOS|497258**	**239367_at**	**-0.726161679**	**0.36283173**	**0.045352193**
GTPBP4|23560	218239_s_at	1.285567087	0.646094714	0.04661846
SCAPER|49855	215848_at	0.941418527	0.473397523	0.046740655
C8orf79|57604	239297_at	-0.379856706	0.191035154	0.046766077

Supplement S1 to S15

S4. Require Consistency in Candidate Transcripts in Training Cohorts.

Table S4 provides a summary of the candidate transcripts with a consistent relationship with nodal metastasis in both the Training-1 and the Training-2 Cohorts. There were 23 transcripts with concordant coefficients from univariate logistic regression modeling in Training-1 and Training-2. That is both coefficients were either negative or positive. High expression of a transcript with a negative coefficient was associated with stage I disease (no metastasis). High expression of a transcript with a positive coefficient was associated with nodal or distant metastasis. The seven transcripts in the final MS7 classifier are bolded and highlighted in blue.

Table S4.

	Univariate Logistic Modeling for Metastasis		
	Training-1 (RNA-sequencing)	Training-2 (Affymetrix microarray)	
**GeneSymbol|ID**	**Coefficient**	**Probeset**	**Coefficient**
MRPL37|51253	1.595857579	218887_at	2.509100598
GTPBP4|23560	1.427071368	218239_s_at	1.285567087
**MLLT10|8028**	1.2737948	216503_s_at	1.331562976
PTPLAD1|51495	1.271930639	217777_s_at	0.773490471
**RSRC1|51319**	1.076378266	235354_s_at	0.84580908
SCAPER|49855	0.940632294	215848_at	0.941418527
LDLR|3949	0.715178001	214170_x_at	1.877044253
TFRC|7037	0.698434467	237214_at	4.219036884
SCD|6319	0.69767617	211708_s_at	1.348364253
HOXD13|3239	0.399495199	207398_at	2.901486206
**PDLIM3|27295**	0.383761369	209621_s_at	1.873951228
C8orf79|57604	-0.387423797	239297_at	-0.379856706
**APOL4|80832**	-0.465935125	223801_s_at	-1.061512979
**BDNFOS|497258**	-0.736747975	239367_at	-0.726161679
COL18A1|80781	-0.845828665	209081_s_at	-0.999425929
ZNF10|7556	-0.933163492	229848_at	-1.069343172
**ZNF596|169270**	-0.955900968	232641_at	-1.123630563
ZFP36L2|678	-1.085996313	201368_at	-0.57804217
SFRS2B|10929	-1.278118474	229471_s_at	-3.262596536
ZNF140|7699	-1.285322946	204523_at	-1.602120944
LUC7L|55692	-1.285625942	1557066_at	-1.05678706
**TBRG1|84897**	-1.38213156	230681_at	-0.962005274
SLC35E2|728661	-1.768533935	217122_s_at	-1.306519776

Supplement S1 to S15

S5. Nodal and Distant Metastasis Prediction with Clinical Factors and/or Molecular Features in Training-1.

Table S5 illustrates the accuracy for predicting nodal and distant metastasis first for the MS7 score and a select set of clinical and molecular features in the Training-1 Cohort, and then for MS7 ± G3 ± MI to predict metastasis in the Validation-1, Training-2 and Validation-2 Cohorts. The clinical factors under investigation in the Training-1 Cohort included age at diagnosis, grade 3 (G3) disease, and myometrial invasion (MI) categorized as <50% vs ≥50%. Three aggressive molecular subtypes defined by the UCEC TCGA Research Network were evaluated in the Training-1 Cohort including copy number variant high (CNV) subtype, somatic copy number alterations (SCNA) cluster 4 subtype or the transcript-based mitotic molecular subtype. RNAseq-based transcript expression data for *ESR1*, *ARID1A*, *CTNNB1*, *KRAS*, *MKI67*, *PIK3CA* and sequencing data for mutation in TP53 or PTEN were also repurposed from the UCEC TCGA Research Network to evaluate their relationship with nodal and distant metastasis in the Training-1 Cohort.

MS7 was the best predictor of nodal and distant metastasis in the Training-1 Cohort with an AUC (95% CI) of 0.89 (0.80-0.98) as a continuous variable or 0.87 (0.97-0.96) as a categorical variable followed by 0.69 (0.59-0.80) for MI, 0.66 (0.55-0.77) for G3 disease and 0.71 (0.58-0.83) for *ESR1* transcript expression. Age at diagnosis and the other molecular features were then dropped from further consideration. Next, we compared the accuracy of companion diagnostic models that combined MS7 score ± G3 ± MI to predict nodal and distant metastasis in the Training-1 cohort. The AUC (95% CI) was 0.89 (0.80-0.98) for both the MS7+G3 combination and the model with MS7 alone and was 0.92 (0.85-0.99) for both the MS7+MI combination and the MS7+MI+G3 combination. Transcript expression of *ESR1* exhibited a higher univariate AUC than

G3 or MI but did not improve the predictive accuracy when combined with MS7 (AUC 0.89, 95% CI 0.81-0.97) or when combined with MS7 and MI (AUC 0.92, 95% CI 0.86-0.99).

Table S5. Predicting nodal and distant metastasis in endometrioid endometrial cancers.*

COHORT	Prediction Model ^	*AUC*	*95% CI*
Training-1	MS7 Score	**0.89**	**0.80-0.98**
	Age at diagnosis	0.56	0.43-0.70
	G3 (yes/no)	**0.66**	**0.55-0.77**
	MI (yes/no)	**0.69**	**0.59-0.80**
	CNV High Subtype (yes/no)	0.54	0.45-0.64
	SCNA Cluster 4 Subtype (yes/no)	0.58	0.49-0.66
	Mitotic Subtype (yes/no)	**0.62**	**0.50-0.74**
	ESR1 Expression	**0.71**	**0.58-0.83**
	ARID1A Expression	0.55	0.41-0.69
	CTNNB1 Expression	**0.65**	**0.52-0.78**
	KRAS Expression	**0.65**	**0.52-0.77**
	MKI67 Expression	0.59	0.46-0.73
	PIK3CA Expression	**0.64**	**0.51-0.78**
	TP53 Mutation (yes/no)	0.58	0.46-0.70
	PTEN Mutation (yes/no)	0.60	0.48-0.73
Training-1	MS7 Score + G3 (yes/no)	**0.89**	**0.80-0.98**
	MS7 Score + MI (yes/no)	**0.92**	**0.85-0.99**
	MS7 Score + MI (yes/no) + G3 (yes/no)	**0.92**	**0.85-0.99**
	MS7 Score + *ESR1* Expression	**0.89**	**0.81-0.97**
	ESR1 Expression + MI (yes/no)	**0.78**	**0.67-0.89**
	MS7 Score + MI (yes/no) + *ESR1* Expression	**0.92**	**0.86-0.99**

* Models made up of one, two or three variables were evaluated based on their accuracy in predicting nodal and distant metastasis using area under the curve (AUC) and 95% confidence interval (CI) from a receiver operating characteristic curve. Bolding was used to highlight significant relationships with *P-value <0.05*.

^ The MS7 score was calculated using the platform-centric algorithm presented in Methods and evaluated per unit increase in the score. Evaluations were also performed for age at diagnosis in years, grade 3 (G3) disease, ≥ 50% myometrial invasion (MI), the aggressive copy number variant (CNV) high subtype, the aggressive somatic copy number alteration (SCNA) cluster 4 subtype, the aggressive transcriptic-based mitotic subtype, RNA sequencing based transcriptic expression of estrogen receptor (*ESR1*), *ARID1A*, *CTNNB1*, *KRAS*, *MKI67* or *PIK3CA*, or mutations in *TP53* or *PTEN*. Modeling using the aggressive molecular subtypes or using molecular features other than MS7 was restricted to the Training-1 cohort. Mutations in *ARID1A*, *CTNNB1*, *KRAS*, *MKI67* and *PIK3CA* were also evaluated but none of these molecular features exhibited a significant relationship with nodal and distant metastasis in the Training-1 cohort and were not included in this table.

Supplement S1 to S15

Data for endometrioid endometrial cancer patients with local (N=55,417) vs. distant (N=3,324) disease were then downloaded from the Surveillance, Epidemiology, and End Results (SEER) Program to determine a population-based estimate for the accuracy of grade 3 (G3) disease to predict metastatic disease relative to that seen in our Training and Validation Cohorts. The area under the curve (AUC) and the 95% confidence interval (95% CI) for G3 disease were estimated from the receiver operator characteristics (ROC) analysis as 0.686 and 0.675-0.697 in SEER suggesting that the predictive accuracy for G3 in the Training-1, Validation-1 and the Training-2 cohorts were reasonable whereas that in the Validation-2 cohort was skewed. The poor performance of G3 disease in Training-2, however, likely reflected at least in part the higher proportion of stage I with G3 disease in this cohort. The converse is also true, enhanced performance of G3 in Validation-2 likely reflect the lower proportion of stage I with G3 disease in this cohort.

Receiver operating characteristics curves were also generated for age of diagnosis, race and ethnicity in the SEER cohort of endometrioid endometrial cancer patients and compared to that provided by G3 disease. The AUC ± standard error (SE) for age at diagnosis was 0.532 ± 0.005 while that for race was 0.520 ± 0.005 and ethnicity was 0.509 ± 0.005 all substantially inferior to G3. Age at diagnosis, race and ethnicity were therefore not further included in our predictive modeling for nodal and distant metastasis in endometrioid endometrial cancer patients.

S6. Odds of Nodal and Distant Metastasis based on the MS7 Classifier, Grade 3 and/or Myometrial Invasion

This section provides the odds ratio and 95% confidence interval for nodal and distant metastasis using the MS7 Classifier, grade 3 (G3) disease, and/or myometrial invasion ≥50% (MI) in the Training-1 and Training-2 Cohorts with RNA sequencing data, and for the Training-2 and Validation-2 Cohorts with Affymetrix Microarray data (Table S12).

Table S6. Odds ratio and area under the curve estimation for nodal and distant metastasis in endometrioid endometrial cancer patients using the MS7 metastasis score (based either on RNA sequencing data or Affymetrix Microarray data), grade 3 disease, and/or ≥50% myometrial invasion.

TYPE OF MODEL		**RNA Sequencing Data**		**Affymetrix Microarray Data**	
		Training-1 [75 Cases]	*Validation-1 [245 Cases]*	*Training-2 [64 Cases]*	*Validation-2 [81 Cases]*
MS7 Only		**OR 2.72**; 1.83-4.62, *P*<***0.001***	**OR 1.51**; 1.18-2.00, *P*=***0.002***	**OR 2.72**; 1.75-4.92, *P*<***0.001***	**OR 1.97**; 1.32-3.39, *P*=***0.004***
G3 Only		**OR 3.92**; 1.47-10.93, *P*=***0.007***	**OR 4.44**; 1.29-20.38, *P*=***0.028***	OR 1.64; 0.61-4.53, *P*=0.332	**OR 7.86**; 2.16-33.12, *P*=***0.003***
MI Only		**OR 6.06**; 2.07-20.76, *P*=***0.002***	**OR 6.79**; 1.90-31.5, *P*=***0.006***	**OR 3.69**; 1.28-16.01, *P*=***0.023***	**OR 9.94**; 2.62-49.00, *P*=***0.002***
MS7+G3	MS7	**OR 2.69**; 1.79-4.62, ***<0.001***	**OR 1.43**; 1.11-1.91, *P*=***0.008***	**OR 2.91**; 1.81-5.63, *P*<***0.001***	**OR 1.75**; 1.15-3.12, *P*=***0.025***
	G3	OR 1.12; 0.27-4.38, *P*=0.873	OR 2.74; 0.74-13.12, *P*=0.156	OR 0.56; 0.12-2.28, *P*=0.438	**OR 4.51**; 1.08-20.61, *P*=***0.041***
MS7+MI	MS7	**OR 2.85**; 1.83-5.26, *P*<***0.001***	**OR 1.37**; 1.06-1.82, *P*=***0.021***	**OR 2.59**; 1.69-4.67, *P*<***0.001***	**OR 1.95**; 1.23-3.57, *P*=***0.013***
	MI	**OR 7.70**; 1.59-53.48, *P*=***0.020***	**OR 4.81**; 1.26-23.21, *P*=***0.029***	**OR 5.37**; 1.28-26.35, *P*=***0.026***	**OR 7.51**; 1.76-41.80, *P*=***0.010***
MS7+G3+MI	MS7	**OR 2.91**; 1.84-5.43, *P*<***0.001***	**OR 1.32**; 1.02-1.76, *P*=***0.044***	**OR 2.86**; 1.78-5.59, *P*<***0.001***	**OR 1.75**; 1.09-3.25, *P*=***0.040***
	G3	OR 0.72; 0.15-3.03, *P*=0.656	OR 2.70; 0.70-13.11, *P*=0.169	OR 0.38; 0.06-1.79, *P*=0.249	OR 3.03; 0.63-15.03, *P*=0.163
	MI	**OR 8.20**; 1.65-58.47, *P*=***0.018***	**OR 7.72**; 1.25-22.77, *P*=***0.030***	**OR 6.42**; 1.46-34.30, *P*=***0.018***	**OR 5.92**; 1.30-34.01, *P*=***0.028***

Relationship with nodal or distant metastasis expressed as odds ratio (OR) and 95% confidence interval (CI) from logistic regression modeling using profile-likelihood method or area under the curve (AUC) and 95% CI interval from a receiver operating characteristic curve. Bolding is used to highlight significant relationships with *P-value <0.05*.

The MS7 metastasis score was calculated using the platform-centric algorithm presented in Methods and evaluated per unit increase in the score. Grade 3 disease was abbreviated G3. Myometrial invasion ≥50% was abbreviated MI.

S7. Raw Data for Evaluating Risk of Metastasis in Validation-1 and Validation-2.

Table S6 provides a summary of the data that was used to evaluate risk of metastasis using the MS7 score, grade 3 disease and/or myometrial invasion categorized as < vs ≥ 50%.

Table S7. Raw Data for Evaluating Risk of Metastasis in the 245 Validation-1 Cases and the 81 Validation-2 Cases

Cohort	Sample name	Metastasis (Stage IIIC/IV)	Grade 3 Disease	MI ≥ 50%	MS7 Score	MS7 Group
V1	GYNCOE01	No	No	Yes	-1.99991	1
V1	GYNCOE10	No	No	Yes	-1.69131	1
V1	GYNCOE11	No	No	No	2.250012	1
V1	GYNCOE12	Yes	Yes	No	-0.09079	1
V1	GYNCOE13	Yes	Yes	Yes	3.003566	1
V1	GYNCOE14	No	No	No	-1.30353	1
V1	GYNCOE15	No	Yes	No	-3.25468	0
V1	GYNCOE02	No	Yes	No	-0.08021	1
V1	GYNCOE03	Yes	No	Yes	-1.51882	1
V1	GYNCOE04	No	No	Yes	-0.43465	1
V1	GYNCOE05	No	No	No	-3.11658	0
V1	GYNCOE06	Yes	No	Yes	-2.95746	0
V1	GYNCOE07	Yes	Yes	No	1.802693	1
V1	GYNCOE08	Yes	No	No	-1.4863	1
V1	GYNCOE09	Yes	Yes	Yes	6.353986	1
V1	TCGA_A5_A0GB	No	Yes		0.034768	1
V1	TCGA_A5_A0GD	No	No	No	-8.45536	0
V1	TCGA_A5_A0GE	No	No	No	-2.15866	0
V1	TCGA_A5_A0GH	No	Yes	No	1.924428	1
V1	TCGA_A5_A0GI	No	No	No	-4.55785	0
V1	TCGA_A5_A0GJ	No	No	No	-3.48305	0
V1	TCGA_A5_A0GM	No	No	No	-4.68892	0
V1	TCGA_A5_A0GN	No	No	Yes	-3.00377	0
V1	TCGA_A5_A0GP	No	No	No	-0.97583	1
V1	TCGA_A5_A0GQ	No	No	Yes	-7.1368	0
V1	TCGA_A5_A0GR	No	No	No	-2.02109	0
V1	TCGA_A5_A0GU	No	No	No	-6.39266	0
V1	TCGA_A5_A0GV	No	No	No	-8.70338	0
V1	TCGA_A5_A0GW	No	Yes	No	-1.45237	1
V1	TCGA_A5_A0GX	No	No	No	-1.64199	1
V1	TCGA_A5_A0R7	No	No	No	-3.45006	0
V1	TCGA_A5_A0R8	No	No	Yes	-3.88646	0

V1	TCGA_A5_A0R9	No	No	No	-3.93718	0
V1	TCGA_A5_A0RA	No	No	Yes	-10.912	0
V1	TCGA_A5_A0VO	No	No	Yes	-2.43665	0
V1	TCGA_A5_A0VP	No	Yes	No	-2.97798	0
V1	TCGA_A5_A0VQ	No	Yes	No	-4.75546	0
V1	TCGA_A5_A1OJ	No	No	No	-2.85186	0
V1	TCGA_A5_A2K5	No	Yes	No	-5.84854	0
V1	TCGA_A5_A3LO	No	Yes	Yes	-0.40974	1
V1	TCGA_AJ_A23O	No	Yes	Yes	-1.42696	1
V1	TCGA_AJ_A2QK	No	Yes	No	-1.20479	1
V1	TCGA_AJ_A2QO	No	Yes	Yes	-1.54452	1
V1	TCGA_AJ_A3BH	No	Yes	Yes	0.810693	1
V1	TCGA_AJ_A3BI	No	Yes	No	-6.02388	0
V1	TCGA_AJ_A3BK	No	Yes	Yes	-4.73498	0
V1	TCGA_AJ_A3EK	No	Yes	No	1.985956	1
V1	TCGA_AJ_A3EL	No	Yes	No	-1.68418	1
V1	TCGA_AJ_A3EM	No	Yes	No	1.288767	1
V1	TCGA_AJ_A3NC	No	Yes	No	-2.91078	0
V1	TCGA_AJ_A3NE	No	Yes	No	-7.72561	0
V1	TCGA_AJ_A5DV	No	Yes	No	-2.71609	0
V1	TCGA_AJ_A5DW	No	No	No	-4.2173	0
V1	TCGA_AP_A051	No	Yes	No	-3.59781	0
V1	TCGA_AP_A053	No	Yes	No	-0.62866	1
V1	TCGA_AP_A059	No	Yes	No	-1.21211	1
V1	TCGA_AP_A05N	No	No	No	-0.9967	1
V1	TCGA_AP_A05O	No	Yes	Yes	-1.32408	1
V1	TCGA_AP_A05P	No	No	No	-2.5996	0
V1	TCGA_AP_A0LG	No	Yes	No	-2.10156	0
V1	TCGA_AP_A0LJ	No	No	No	-3.70539	0
V1	TCGA_AP_A0LL	No	No	No	-5.99915	0
V1	TCGA_AP_A0LN	No	No	No	-5.32793	0
V1	TCGA_AP_A0LO	No	No	No	-3.01879	0
V1	TCGA_AP_A0LS	No	No	No	-3.70811	0
V1	TCGA_AP_A0LV	No	No	No	-2.86553	0
V1	TCGA_AP_A1DH	No	Yes	No	-1.04252	1
V1	TCGA_AP_A1DO	No	No	No	-6.77194	0
V1	TCGA_AP_A1DP	No	No	No	-3.15469	0
V1	TCGA_AP_A1E1	No	No	No	-6.76169	0
V1	TCGA_AP_A1E4	No	No	Yes	2.015109	1

V1	TCGA_B5_A0JS	No	No	No	-2.77454	0
V1	TCGA_B5_A0JU	No	No	No	-2.29838	0
V1	TCGA_B5_A0JV	No	No	No	0.093947	1
V1	TCGA_B5_A0K0	No	No	No	-3.52513	0
V1	TCGA_B5_A0K1	No	No	No	-10.4341	0
V1	TCGA_B5_A0K3	No	Yes	No	-0.18674	1
V1	TCGA_B5_A0K4	No	No	Yes	-1.49941	1
V1	TCGA_B5_A0K6	No	No	No	-5.26702	0
V1	TCGA_B5_A0K7	No	No	No	-8.18689	0
V1	TCGA_B5_A0KB	No	Yes	Yes	-1.4714	1
V1	TCGA_B5_A11E	No	Yes	Yes	-0.16638	1
V1	TCGA_B5_A11F	No	No	No	-2.56054	0
V1	TCGA_B5_A11G	No	No	No	-5.33571	0
V1	TCGA_B5_A11J	No	No	No	-1.20889	1
V1	TCGA_B5_A11L	No	No	Yes	1.555711	1
V1	TCGA_B5_A11M	No	No	No	-1.95248	1
V1	TCGA_B5_A11N	No	No	No	-2.80691	0
V1	TCGA_B5_A11O	No	No	No	-2.6482	0
V1	TCGA_B5_A11P	No	Yes	No	-3.3191	0
V1	TCGA_B5_A11S	No	No	No	-4.05162	0
V1	TCGA_B5_A11V	No	No	No	-3.67245	0
V1	TCGA_B5_A11W	No	No	Yes	-3.32422	0
V1	TCGA_B5_A11Y	No	No	Yes	-3.53357	0
V1	TCGA_B5_A11Z	No	No	No	-6.60977	0
V1	TCGA_B5_A1MV	No	No	No	-1.59285	1
V1	TCGA_B5_A1MW	No	Yes	No	-1.3411	1
V1	TCGA_B5_A1MX	No	Yes	Yes	-2.11287	0
V1	TCGA_B5_A1MZ	No	No	No	-2.4231	0
V1	TCGA_B5_A3F9	No	Yes	No	-4.32953	0
V1	TCGA_B5_A3FA	No	Yes	Yes	-4.15976	0
V1	TCGA_B5_A3FB	No	Yes	No	-4.47101	0
V1	TCGA_B5_A3FD	No	Yes	No	-2.20035	0
V1	TCGA_B5_A3FH	No	Yes	No	-1.55053	1
V1	TCGA_BG_A0LW	No	No	No	-2.55397	0
V1	TCGA_BG_A0LX	No	No	Yes	0.004069	1
V1	TCGA_BG_A0M0	No	No	No	-8.10441	0
V1	TCGA_BG_A0M2	No	No	Yes	-2.80184	0
V1	TCGA_BG_A0M3	No	No	Yes	-4.53219	0
V1	TCGA_BG_A0M4	No	Yes	No	-1.54173	1

V1	TCGA_BG_A0M8	No	Yes	Yes	-2.56549	0
V1	TCGA_BG_A0M9	No	No	Yes	-3.676	0
V1	TCGA_BG_A0MA	No	Yes	Yes	-2.68757	0
V1	TCGA_BG_A0MC	No	No	No	-3.61877	0
V1	TCGA_BG_A0MH	No	No	Yes	2.397145	1
V1	TCGA_BG_A0MI	No	No	No	-4.19167	0
V1	TCGA_BG_A0MO	No	No	No	-2.72976	0
V1	TCGA_BG_A0MQ	No	No	No	-6.84013	0
V1	TCGA_BG_A0MT	No	No	No	-6.98581	0
V1	TCGA_BG_A0RY	No	No	Yes	-6.99606	0
V1	TCGA_BG_A0VV	No	No	Yes	-3.90228	0
V1	TCGA_BG_A0VW	No	No	Yes	-5.16103	0
V1	TCGA_BG_A0VX	No	No	No	-3.68517	0
V1	TCGA_BG_A0W2	No	No	No	-3.33433	0
V1	TCGA_BG_A0YU	No	No	Yes	-5.10484	0
V1	TCGA_BG_A187	No	No	Yes	-2.82922	0
V1	TCGA_BG_A18A	No	No	Yes	0.585632	1
V1	TCGA_BG_A18B	No	No	No	-1.72855	1
V1	TCGA_BG_A18C	No	No	No	-1.82771	1
V1	TCGA_BG_A220	No	Yes	No	-4.98516	0
V1	TCGA_BG_A221	No	Yes		0.519427	1
V1	TCGA_BG_A222	No	Yes	No	-0.97915	1
V1	TCGA_BG_A2AE	No	Yes		-0.93293	1
V1	TCGA_BG_A2L7	No	Yes	No	-3.30823	0
V1	TCGA_BK_A0C9	No	No	No	-0.61616	1
V1	TCGA_BK_A139	No	Yes		1.453986	1
V1	TCGA_BK_A13B	No	Yes		-3.02196	0
V1	TCGA_BK_A13C	No	No	No	-1.5707	1
V1	TCGA_BK_A4ZD	No	Yes	No	-2.25358	0
V1	TCGA_BK_A56F	No	No	Yes	-0.881	1
V1	TCGA_BS_A0TD	No	No	No	-4.16363	0
V1	TCGA_BS_A0U5	No	No	No	-4.66379	0
V1	TCGA_BS_A0U7	No	No	Yes	-0.39354	1
V1	TCGA_BS_A0UA	No	No	No	-2.36491	0
V1	TCGA_BS_A0UJ	No	No	No	-8.30123	0
V1	TCGA_BS_A0UL	No	No	No	1.000281	1
V1	TCGA_BS_A0UM	No	No	No	-2.81168	0
V1	TCGA_BS_A0V7	No	No	No	-9.79918	0
V1	TCGA_D1_A0ZN	No	No	No	-1.16449	1

V1	TCGA_D1_A0ZO	No	No	No	-0.03619	1
V1	TCGA_D1_A0ZQ	No	No	Yes	2.055545	1
V1	TCGA_D1_A0ZR	No	No	No	-4.09242	0
V1	TCGA_D1_A0ZS	No	Yes	No	3.118761	1
V1	TCGA_D1_A0ZU	No	No	No	-0.90316	1
V1	TCGA_D1_A0ZV	No	No	No	-4.15704	0
V1	TCGA_D1_A101	No	No	No	-0.917	1
V1	TCGA_D1_A102	No	No	No	-5.26199	0
V1	TCGA_D1_A103	No	No	No	-0.76019	1
V1	TCGA_D1_A15Z	No	No	No	-4.68719	0
V1	TCGA_D1_A160	No	No	No	-2.78923	0
V1	TCGA_D1_A161	No	No	Yes	-2.2873	0
V1	TCGA_D1_A167	No	No	Yes	-2.82223	0
V1	TCGA_D1_A16D	No	No	Yes	-2.71114	0
V1	TCGA_D1_A16E	No	No	No	-4.29673	0
V1	TCGA_D1_A16F	No	No	No	-0.61819	1
V1	TCGA_D1_A16O	No	No	No	-2.98863	0
V1	TCGA_D1_A16R	No	No	No	-3.74809	0
V1	TCGA_D1_A16X	No	No	No	-4.18105	0
V1	TCGA_D1_A16Y	No	No	No	-2.79137	0
V1	TCGA_D1_A174	No	Yes	No	-0.89365	1
V1	TCGA_D1_A175	No	Yes	Yes	-2.21332	0
V1	TCGA_D1_A176	No	No	No	-3.95064	0
V1	TCGA_D1_A177	No	No	Yes	-1.18643	1
V1	TCGA_D1_A17B	No	No	No	-0.52702	1
V1	TCGA_D1_A17C	No	No	No	-0.35747	1
V1	TCGA_D1_A17D	No	Yes	Yes	-3.35997	0
V1	TCGA_D1_A17F	No	No	No	-4.46695	0
V1	TCGA_D1_A17H	No	No	Yes	-2.12812	0
V1	TCGA_D1_A17K	No	No	No	-0.68156	1
V1	TCGA_D1_A17L	No	No	No	-9.73906	0
V1	TCGA_D1_A17N	No	No	No	-2.83232	0
V1	TCGA_D1_A17Q	No	No	No	-3.25261	0
V1	TCGA_D1_A17R	No	No	No	-2.75318	0
V1	TCGA_D1_A17S	No	No	No	-7.5764	0
V1	TCGA_D1_A17T	No	No	No	-8.23572	0
V1	TCGA_D1_A1NS	No	No	No	-4.64295	0
V1	TCGA_D1_A1NY	No	Yes	Yes	-1.18323	1
V1	TCGA_D1_A1NZ	No	No	No	-3.38689	0

V1	TCGA_D1_A1O0	No	Yes	No	-2.99752	0
V1	TCGA_D1_A1O5	No	No	No	-3.95213	0
V1	TCGA_D1_A1O7	No	No	No	-1.99442	1
V1	TCGA_D1_A2G5	No	Yes	No	-1.80681	1
V1	TCGA_D1_A3DH	No	Yes	No	-1.14067	1
V1	TCGA_DF_A2KN	No	Yes	Yes	0.575592	1
V1	TCGA_DF_A2KR	Yes	Yes	Yes	-1.04372	1
V1	TCGA_DF_A2KS	No	Yes	No	1.859235	1
V1	TCGA_DF_A2KU	No	Yes	No	-5.66909	0
V1	TCGA_DF_A2KV	No	Yes	No	-7.36277	0
V1	TCGA_DF_A2KY	Yes	Yes	Yes	-0.58299	1
V1	TCGA_DF_A2KZ	No	Yes	No	-1.00846	1
V1	TCGA_DF_A2L0	Yes	Yes	Yes	-7.13944	0
V1	TCGA_DI_A0WH	No	Yes	No	-5.39984	0
V1	TCGA_DI_A1C3	No	No	No	-3.43945	0
V1	TCGA_E6_A1LX	No	Yes	No	0.882908	1
V1	TCGA_E6_A2P9	No	Yes	No	-0.74738	1
V1	TCGA_EC_A1NJ	No	No	No	-1.96284	1
V1	TCGA_EC_A1QX	No	Yes		-1.27372	1
V1	TCGA_EO_A1Y7	No	Yes	Yes	-1.31768	1
V1	TCGA_EO_A22R	No	Yes	Yes	0.91137	1
V1	TCGA_EO_A22S	No	Yes	Yes	0.759266	1
V1	TCGA_EO_A22U	No	Yes	Yes	0.775061	1
V1	TCGA_EO_A22Y	No	Yes	Yes	-2.54972	0
V1	TCGA_EO_A3AS	No	Yes	No	-1.76708	1
V1	TCGA_EO_A3AV	Yes	Yes	Yes	-0.9307	1
V1	TCGA_EO_A3AY	No	Yes	Yes	-5.9897	0
V1	TCGA_EO_A3B0	Yes	Yes		1.396153	1
V1	TCGA_EO_A3KX	No	Yes	No	-3.32707	0
V1	TCGA_EO_A3L0	No	Yes	No	-2.26909	0
V1	TCGA_EY_A1G8	No	Yes	No	-5.67838	0
V1	TCGA_EY_A1GC	No	Yes	No	-2.04149	0
V1	TCGA_EY_A1GD	No	No	No	-2.61499	0
V1	TCGA_EY_A1GE	No	No	No	-6.66196	0
V1	TCGA_EY_A1GF	No	No	No	-3.31335	0
V1	TCGA_EY_A1GH	No	No	No	-4.70853	0
V1	TCGA_EY_A1GI	No	No	Yes	0.91291	1
V1	TCGA_EY_A1GK	No	Yes	Yes	0.698147	1
V1	TCGA_EY_A1GP	No	No	No	-0.06008	1

V1	TCGA_EY_A1GQ	No	Yes	Yes	0.944542	1
V1	TCGA_EY_A1GR	No	Yes	No	-0.09252	1
V1	TCGA_EY_A1GU	No	No	Yes	-3.83009	0
V1	TCGA_EY_A215	No	Yes	Yes	-3.13588	0
V1	TCGA_EY_A2OM	No	Yes	No	-3.16227	0
V1	TCGA_EY_A2OP	No	Yes	No	-0.80962	1
V1	TCGA_EY_A2OQ	No	Yes	No	-3.85787	0
V1	TCGA_EY_A547	No	Yes	No	-1.15968	1
V1	TCGA_EY_A548	No	No	No	-4.63278	0
V1	TCGA_EY_A549	No	Yes	No	-4.61489	0
V1	TCGA_EY_A5W2	No	Yes	Yes	-3.44798	0
V1	TCGA_FI_A2D0	No	Yes	No	-3.1207	0
V1	TCGA_FI_A2D6	No	Yes	Yes	1.570423	1
V1	TCGA_FI_A2F4	No	Yes	Yes	-6.76582	0
V1	TCGA_FI_A2F9	No	Yes	No	0.3813	1
V1	TCGA_H5_A2HR	No	Yes	No	-6.7738	0
V1	TCGA_SL_A6J9	No	Yes	Yes	-1.99676	1
V1	TCGA_SL_A6JA	No	Yes	Yes	-10.9793	0
V2	V2-001	Yes	Yes	Yes	-2.3382	1
V2	V2-002	Yes	Yes	Yes	-3.79634	1
V2	V2-003	No	No	Yes	-4.64996	0
V2	V2-004	Yes	Yes	No	-0.28308	1
V2	V2-005	No	No	Yes	-6.4336	0
V2	V2-006	No	No	No	-5.11437	0
V2	V2-007	No	Yes	Yes	-3.92491	1
V2	V2-008	Yes	No	Yes	-4.79312	0
V2	V2-009	No	No	Yes	-3.43526	1
V2	V2-010	No	Yes	No	-4.09785	1
V2	V2-011	No	Yes	No	-9.95483	0
V2	V2-012	Yes	No	Yes	-5.51439	0
V2	V2-013	No	No	No	-3.63301	1
V2	V2-014	Yes	Yes	Yes	-0.51336	1
V2	V2-015	No	No	No	-4.33161	0
V2	V2-016	No	Yes	No	-5.19149	0
V2	V2-017	No	No	Yes	-5.36758	0
V2	V2-018	Yes	No	Yes	-4.15388	1
V2	V2-019	No	No	No	-3.91468	1
V2	V2-020	No	No	No	-12.3511	0
V2	V2-021	No	No	No	-7.58965	0

V2	V2-022	No	No	No	-4.90618	0
V2	V2-023	No	No	No	-2.64352	1
V2	V2-024	No	No	No	-4.50662	0
V2	V2-025	No	Yes	Yes	-3.626	1
V2	V2-026	No	No	No	-10.4927	0
V2	V2-027	No	Yes	No	-3.91705	1
V2	V2-028	No	No	No	-3.93342	1
V2	V2-029	No	No	No	-8.19999	0
V2	V2-030	No	Yes	Yes	-6.52674	0
V2	V2-031	No	No	No	-5.79328	0
V2	V2-032	No	No	No	-6.66609	0
V2	V2-033	No	No	No	-2.53269	1
V2	V2-034	No	No	No	-5.5572	0
V2	V2-035	No	No	No	-3.37735	1
V2	V2-036	No	No	No	-6.14482	0
V2	V2-037	No	Yes	Yes	-3.28338	1
V2	V2-038	No	Yes	No	-4.16479	1
V2	V2-039	No	Yes	No	-5.56891	0
V2	V2-040	No	No	No	-6.37766	0
V2	V2-041	No	Yes	Yes	-2.83556	1
V2	V2-042	No	No	No	-4.49953	0
V2	V2-043	No	Yes	Yes	-5.19278	0
V2	V2-044	No	No	Yes	-5.05525	0
V2	V2-045	No	No	No	-5.06386	0
V2	V2-046	No	No	No	-7.0635	0
V2	V2-047	No	No	No	-6.39939	0
V2	V2-048	No	No	No	-4.60869	0
V2	V2-049	No	No	No	-3.59034	1
V2	V2-050	Yes	No	No	-3.39768	1
V2	V2-051	No	No	No	-8.18358	0
V2	V2-052	No	No	No	-4.76607	0
V2	V2-053	No	No	No	-5.79173	0
V2	V2-054	No	No	Yes	-3.72208	1
V2	V2-055	No	No	Yes	-5.38245	0
V2	V2-056	No	No	No	-3.31822	1
V2	V2-057	No	No	No	-4.72735	0
V2	V2-058	No	No	No	-7.87303	0
V2	V2-059	No	No	No	-8.12095	0
V2	V2-060	No	No	No	-3.84654	1

V2	V2-061	No	No	No	-3.40411	1
V2	V2-062	No	No	No	-4.3869	0
V2	V2-063	No	No	No	-5.35806	0
V2	V2-064	No	No	No	-7.54247	0
V2	V2-065	No	No	No	-8.24553	0
V2	V2-066	No	Yes	No	-3.41936	1
V2	V2-067	No	No	Yes	-2.68217	1
V2	V2-068	No	No	Yes	-4.57305	0
V2	V2-069	No	No	No	-1.96757	1
V2	V2-070	No	No	Yes	-4.09893	1
V2	V2-071	No	No	No	-9.27302	0
V2	V2-072	No	No	No	-3.74638	1
V2	V2-073	No	No	No	-8.95212	0
V2	V2-074	No	No	No	-7.83637	0
V2	V2-075	No	No	No	-7.51532	0
V2	V2-076	No	Yes	No	-4.90483	0
V2	V2-077	No	No	No	-3.27616	1
V2	V2-078	Yes	Yes	No	-4.22075	1
V2	V2-079	Yes	Yes	Yes	1.284991	1
V2	V2-080	Yes	Yes	Yes	-5.64037	0
V2	V2-081	Yes	Yes	Yes	-3.55309	1

S8. MS7 Classifier Correlations with Cancer Biomarker Expression.

Table S8 provides the discovery and then validation of the correlation between the MS7 classifier and transcript-based expression of cancer biomarkers using RNAseq data in the Training-1 Cohort and then in the Validation-1 Cohort, respectively.

Table S8.

	Training-1 (N=75)		Validation-1 (N=245)	
RNA-sequence based expression	*R*	P	*R*	p
ARID1A	0.303	0.008	0.236	<0.001
CTNNB1	0.396	<0.001	0.246	<0.001
KRAS	0.265	0.022	0.335	<0.001
MKI67	0.387	<0.001	0.315	<0.001
PIK3CA	0.265	0.022	0.335	<0.001
ESR1	-0.421	<0.001	-0.235	<0.001

Spearman's correlation coefficient (*R*).

Supplement S1 to S15

S9. MS7 Classifier Relationship with Mutations in Cancer Biomarkers.

Table S9.1 provides a summary of the relationship between the MS7 classifier and mutations in *ARID1A*, *CTNNB1*, *KRAS*, *PIK3CA*, *TP53* and *PTEN* in 197 TCGA cases that underwent sequencing and mutational analysis by the UCEC TCGA Research network. Table S9.2 shows the triangulated relationship between MS7 level and mutation status for CTNNB1 and TP53 in this cohort.

Table S9.1. Relationship between the MS7 classifier and mutations in TCGA.

	MS7 Low with Cases (%)	MS7 High with Cases (%)	*P* value [1]
ARID1A mutation			0.363
No	80 (63.5)	40 (56.3)	
Yes	46 (36.5)	31 (43.7)	
CTNNB1 mutation			**0.014**
No	71 (56.4)	53 (74.7)	
Yes	55 (43.7)	18 (25.4)	
KRAS mutation			0.124
No	99 (78.6)	48 (67.6)	
Yes	27 (21.4)	23 (32.4)	
PIK3CA mutation			0.655
No	55 (43.7)	34 (47.9)	
Yes	71 (56.4)	37 (52.1)	
TP53 mutation			**0.0009**
No	117 (92.9)	53 (74.7)	
Yes	9 (7.1)	18 (25.4)	
PTEN mutation			0.466
No	24 (19.1)	17 (23.9)	
Yes	102 (81.0)	54 (76.1)	

1. Difference in proportion compared using Fisher's exact test.

Table S9.2. Association between MS7 Level, CTNNB1 Mutation Status and TP53 Mutation Status in TCGA

		CTNNB1 Mutation	
	TP53 Mutation	No	Yes
MS7 Low Patients			
	No	63 (88.7)	54 (98.2)
	Yes	8 (11.3)	1 (1.8)
MS7 High Patients			
	No	39 (73.6)	14 (77.8)
	Yes	14 (26.4)	4 (22.2)
All Patients			
	No	102 (82.3)	68 (93.2)
	Yes	22 (17.7)	5 (6.9)

S10. Evaluations for the MS7 Classifier, Molecular Subtypes and Other Molecular Features

This section shows the significant relationship observed between categorized MS7 score and the aggressive molecular subtypes as defined by the Cancer Genome Atlas (TCGA) Research Network using cases from the Uterine Corpus Endometrial Carcinoma (UCEC) project. **Table S10.1** shows the relationship in the stage I, IIIC or IV cases from TCGA in Training-1 and Validation -1 cohorts with molecular subtype data. The results from **Table S10.1** was presented in the manuscript. **Table S10.2** shows the relationship in all the endometrioid endometrial cancer cases in the TCGA UCEC project with molecular subtype data. **Table S10.3** then displays the relationship with the MS7 classifier or the SCNA Cluster 4 Subtype and nodal and distant metastasis in the stage I, IIIC, or IV cases from TCGA in Training-1 and Validation -1 cohorts with molecular subtype data. The relationship between different biomarkers evaluated by transcript expression categorized by the median or mutation status and either the MS7 classifier categorized using the optimal RNA sequencing-based transcript or with nodal and distant metastasis are displayed in **Table S10.4** for cases in Training-1 or in **Table S10.5** for cases in Validation-1 from TCGA.

Table S10.1 Association of MS7 with TCGA Molecular Subtypes in Stage I, IIIC or IV Endometrioid Endometrial Cancers in the Training-1 or Validation -1 Cohorts

	MS7 Low with Cases (%)	MS7 High with Cases (%)	OR (95% C.I.)	*P* value [1]
CNV High				0.072
No	93 (94.9)	49 (86.0)		
Yes	5 (5.1)	8 (14.0)	3.04 (0.94 – 9.78)	
SCNA Cluster 4				0.015
No	124 (92.5)	83 (81.4)		
Yes	10 (7.5)	19 (18.6)	2.84 (1.26 – 6.41)	
Mitotic				<0.0001
No	111 (91.7)	52 (53.6)		
Yes	10 (8.3)	23 (46.4)	9.61 (4.49 – 20.55)	

Copy number variant (CNV), somatic copy number alterations (SCNA)
1. Difference in proportion compared using Fisher's exact test.

Table S10.2. Association of MS7 with TCGA Molecular Subtypes in Stage I-IV Endometrioid Endometrial Cancer Cases				
	MS7 Low with Cases (%)	MS7 High with Cases (%)	OR (95% C.I.)	*P* value [1]
CNV High				0.027
No	114 (95.0)	56 (84.9)		
Yes	6 (5.0)	10 (15.2)	3.39 (1.17 – 9.81)	
SCNA Cluster 4				0.002
No	154 (93.3)	105 (81.4)		
Yes	11 (6.7)	24 (18.6)	3.20 (1.50 – 6.81)	
Mitotic				<0.0001
No	139 (92.7)	63 (52.1)		
Yes	11 (7.3)	58 (47.9)	11.63 (5.72 – 23.66)	

Copy number variant (CNV), somatic copy number alterations (SCNA)
1. Difference in proportion compared using Fisher's exact test.

Table S10.3 the relationship with the MS7 classifier or the Copy Number Variant (CNV) High Subtype and nodal and distant metastasis in the in the stage I, IIIC or IV cases from TCGA in Training-1 and Validation -1 cohorts with molecular subtype data.			
	Metastasis		
	No. (%)	No. (%)	*P* value
MS7 Group			<0.0001
Low	96 (98.0)	2 (2.0)	
High	38 (66.7)	19 (33.3)	
TCGA Subtype			0.387
Non-CNV high	124 (87.3)	18 (12.7)	
CNV high	10 (76.9)	3 (23.1)	

Table S10.4. The relationship between cancer biomarkers or aggressive molecular subtypes with either MS7 metastasis score based on RNA sequencing data or with nodal and distant metastasis in stage I, stage IIIC or IV endometrioid endometrial cancers from the Cancer Genome Atlas Uterine Corpus Endometrial Cancer Research Network (Training-1)

	**MS7 Score**			**Nodal or Distant Metastasis**			
	Low	High	*P-value*	No	Yes	*P-value*	AUC (95% CI)
Low MS7 Score	25	-	-	23	2	<0.001	0.87 (0.79-0.96)
High MS7 Score	-	50		6	44		
Low *ESR1* Transcripts	10	35	0.023	12	33	0.015	0.65 (0.54-0.76)
High *ESR1* Transcripts	15	15		17	13		
Low *ARID1A* Transcripts	18	25	0.086	17	26	1.000	0.51 (0.39-0.63)
High *ARID1A* Transcripts	7	25		12	20		
Low *CTNNB1* Transcripts	18	23	0.049	18	23	0.348	0.56 (0.44-0.68)
High *CTNNB1* Transcripts	7	27		11	23		
Low *KRAS* Transcripts	15	19	0.088	17	17	0.096	0.61 (0.49-0.72)
High *KRAS* Transcripts	10	31		12	29		
Low *MKI67* Transcripts	15	15	0.023	15	15	0.146	0.60 (0.48-0.71)
High *MKI67* Transcripts	10	35		14	31		
Low *PIK3CA* Transcripts	17	15	0.003	16	16	0.098	0.60 (0.49-0.72)
High *PIK3CA* Transcripts	8	35		13	30		
No *ARID1A* Mutation	9	14	0.523	8	15	0.200	0.62 (0.46-0.77)
ARID1A Mutation	9	8		10	7		
No *CTNNB1* mutation	10	16	0.327	11	15	0.744	0.54 (0.38-0.69)
CTNNB1 Mutation	8	6		7	7		
No *KRAS* Mutation	14	14	0.491	12	16	0.739	0.53 (0.38-0.68)
KRAS Mutation	4	8		6	6		
No *PIK3CA* Mutation	6	12	0.216	6	12	0.216	0.61 (0.45-0.76)
PIK3CA Mutation	12	10		12	10		
No *TP53* Mutation	15	17	0.709	16	16	0.258	0.58 (0.46-0.70)
TP53 Mutation	3	5		2	6		
No *PTEN* Mutation	1	8	0.027	2	7	0.149	0.60 (0.48-0.73)
PTEN Mutation	17	14		16	15		
Non-CNV High Subtype	16	18	0.613	16	18	0.613	0.54 (0.45-0.64)
CNV High Subtype	1	3		1	3		
Non-SCNA Cluster 4 Subtype	22	33	0.303	25	30	0.177	0.58 (0.49-0.66)
SCNA Cluster 4 Subtype	2	9		2	9		
Non-Mitotic Subtype	17	19	0.008	18	18	0.071	0.62 (0.50-0.74)
Mitotic Subtype	4	23		7	20		

MS7 score was categorized as low < -2.00356 (optimal RNA sequencing score) and high with ≥-2.00356. Transcripts categorized at the median with low < median and high as ≥ median. Relationships between categorized variables were evaluated using Fisher' exact method. Relationship with metastasis was also evaluated using area of curve (AUC) with 95% confidence interval (CI) displayed from receiver operator characteristics curve for nodal and distant metastasis. Significant relationships with *P-value* <0.05 were highlighted in bold.

Table S10.5. Relationships between cancer biomarkers or aggressive molecular subtypes with either MS7 metastasis score based on RNA sequencing data or with metastasis in stage I, IIIC, or IV endometrioid endometrial cancers from the Cancer Genome Atlas Uterine Corpus Endometrial Cancer Research Network (Validation-1).

	**MS7 Score**			**Nodal or Distant Metastasis**			
	Low	High	*P-value*	No	Yes	*P-value*	AUC (95% CI)
Low MS7 Score	142	-	-	141	1	0.072	0.71 (0.51-0.91)
High MS7 Score	-	88		84	4		
Low *ESR1* Transcripts	55	53	0.002	105	3	0.668	0.57 (0.32-0.81)
High *ESR1* Transcripts	87	35		120	2		
Low *ARID1A* Transcripts	74	36	0.105	106	4	0.196	0.66 (0.46-0.86)
High *ARID1A* Transcripts	68	52		119	1		
Low *CTNNB1* Transcripts	77	34	0.030	107	4	0.199	0.66 (0.46-0.86)
High *CTNNB1* Transcripts	65	54		118	1		
Low *KRAS* Transcripts	87	32	<0.001	116	3	1.000	0.54 (0.30-0.78)
High *KRAS* Transcripts	55	56		109	2		
Low *MKI67* Transcripts	91	32	<0.001	122	1	0.186	0.67 (0.47-0.87)
High *MKI67* Transcripts	51	56		103	4		
Low *PIK3CA* Transcripts	91	29	<0.001	117	3	1.000	0.54 (0.30-0.78)
High *PIK3CA* Transcripts	51	59		108	2		
No *ARID1A* Mutation	55	20	0.241	75	0	-	-
ARID1A Mutation	31	18		49	0		
No *CTNNB1* mutation	51	28	0.157	79	0	-	-
CTNNB1 Mutation	35	10		45	0		
No *KRAS* Mutation	67	25	0.184	92	0	-	-
KRAS Mutation	19	13		32	0		
No *PIK3CA* Mutation	40	18	1.000	58	0	-	-
PIK3CA Mutation	46	20		66	0		
No *TP53* Mutation	81	29	0.010	110	0	-	-
TP53 Mutation	5	9		14	0		
No *PTEN* Mutation	18	7	0.813	25	0	-	-
PTEN Mutation	68	31		99	0		
Non-CNV High Subtype	77	31	0.131	108	0	-	-
CNV High Subtype	4	5		9	0		
Non-SCNA Cluster 4 Subtype	102	50	0.070	152	0	-	-
SCNA Cluster 4 Subtype	8	10		18	0		
Non-Mitotic Subtype	94	33	<0.001	127	0	-	-
Mitotic Subtype	6	22		28	0		

MS7 score was categorized as low < -2.00356 (optimal RNAseq score) and high with ≥-2.00356. Transcripts categorized as low < median or high ≥ median. Relationships between categorized variables were evaluated using Fisher' exact method. Relationship with metastasis was evaluated using area of curve (AUC) with a 95% confidence interval (CI) displayed from receiver operator characteristics curve for nodal and distant metastasis. Significance set at (*P* <0.05 was highlighted in bold.

S11. Differential Gene Expression in Training-1 Patients with Extreme MS7 Scores

Training-1 patients were first categorized based on MS7 score. Table S9 provides a summary of the genes that were either significantly down regulated or upregulated with a log2 ratio of gene expression less than -2 or greater than +2 in patients with an upper quartile (Q4) vs. a lower quartile (Q1) MS7 score for nodal/distant metastasis, respectively. "Curated Gene Symbol" lists TCGA gene identifiers that were hand-curated using public data sources, such as NCBI, to afford mapping of all significant differentially expressed genes in the Ingenuity Pathway Analysis (IPA) knowledgebase. "Gene symbol IPA" denotes IPA-mapped gene symbols.

Table S11. Differential Gene Expression in Training-1 Patients with Extreme MS7 Scores

Curated Gene Symbol	Gene Symbol IPA	Entrez Gene Name	Q4 versus Q1 (Log_2 Ratio Gene Expression)	q-value
IHH	IHH	indian hedgehog	-5.118	0.006136477
SCGB2A1	SCGB2A1	secretoglobin, family 2A, member 1	-4.627	0.005839523
C4BPA	C4BPA	complement component 4 binding protein, alpha	-4.594	0.0128412
MUC5B	MUC5B	mucin 5B, oligomeric mucus/gel-forming	-4.373	0.02280869
HP	HP	Haptoglobin	-4.193	0.02280869
MSX1	MSX1	msh homeobox 1	-4.153	0.00093901
SERPINA5	SERPINA5	serpin peptidase inhibitor, clade A (alpha-1 antiproteinase, antitrypsin), member 5	-3.936	0.0110201
PYY	PYY	peptide YY	-3.912	0.005133647
C1orf186	C1orf186	chromosome 1 open reading frame 186	-3.862	0.005590417
VWA3A	VWA3A	von Willebrand factor A domain containing 3A	-3.805	0.01136266
SAA1	SAA1	serum amyloid A1	-3.767	0.02941884
PIGR	PIGR	polymeric immunoglobulin receptor	-3.753	0.03403585
HPR	HPR	haptoglobin-related protein	-3.684	0.03047474
DNAJB13	DNAJB13	DnaJ (Hsp40) homolog, subfamily B, member 13	-3.656	0.007247798
SERPINA4	SERPINA4	serpin peptidase inhibitor, clade A (alpha-1 antiproteinase, antitrypsin), member 4	-3.655	0.02196197
SAA2	SAA2	serum amyloid A2	-3.62	0.03047474
MUC13	MUC13	mucin 13, cell surface associated	-3.487	0.01330222
LINC00930	LINC00930	long intergenic non-protein coding RNA 930	-3.439	0.002118548
HNF1B	HNF1B	HNF1 homeobox B	-3.438	0.02538975
SCGB3A1	SCGB3A1	secretoglobin, family 3A, member 1	-3.433	0.04138345
PAX8	PAX8	paired box 8	-3.402	0.01604042
CES3	CES3	carboxylesterase 3	-3.392	0.01777309
KIF12	KIF12	kinesin family member 12	-3.389	0.01561991
GABRP	GABRP	gamma-aminobutyric acid (GABA) A receptor, pi	-3.378	0.01616811
100130933	SMIM6	small integral membrane protein 6	-3.346	0.01604042
ALPPL2	ALPPL2	alkaline phosphatase, placental-like 2	-3.326	0.02803746

WFDC2	WFDC2	WAP four-disulfide core domain 2	-3.302	0.0110201
DACT2	DACT2	dishevelled-binding antagonist of beta-catenin 2	-3.259	0.003307791
AMY1A	AMY1C (includes others)	amylase, alpha 1A (salivary)	-3.215	0.01651241
SPINLW1	EPPIN	epididymal peptidase inhibitor	-3.179	0.0106195
LIMS3	LIMS3/LIMS3L	LIM and senescent cell antigen-like domains 3	-3.161	0.005839523
C10orf81	PLEKHS1	pleckstrin homology domain containing, family S member 1	-3.147	0.03547051
C1orf173	ERICH3	glutamate-rich 3	-3.106	0.01651241
CSF3	CSF3	colony stimulating factor 3 (granulocyte)	-3.106	0.02396597
CCDC33	CCDC33	coiled-coil domain containing 33	-3.08	0.008275055
LIMS3-LOC440895	LIMS3-LOC440895	LIMS3-LOC440895 readthrough	-3.076	0.002204647
TRIM15	TRIM15	tripartite motif containing 15	-3.072	0.01962442
MMP7	MMP7	matrix metallopeptidase 7	-3.069	0.02062955
ALPP	ALPP	alkaline phosphatase, placental	-3.057	0.03935181
OVGP1	OVGP1	oviductal glycoprotein 1, 120kDa	-3.029	0.005839523
CCL20	CCL20	chemokine (C-C motif) ligand 20	-3.018	0.01975898
CLDN2	CLDN2	claudin 2	-2.927	0.02941884
TRIM31	TRIM31	tripartite motif containing 31	-2.885	0.03738103
C10orf107	C10orf107	chromosome 10 open reading frame 107	-2.878	0.009361028
DYNLRB2	DYNLRB2	dynein, light chain, roadblock-type 2	-2.858	0.01304076
PGR	PGR	progesterone receptor	-2.856	0.04138345
PHYHD1	PHYHD1	phytanoyl-CoA dioxygenase domain containing 1	-2.849	0.006626255
HGD	HGD	homogentisate 1,2-dioxygenase	-2.848	0.04756977
ESR1	ESR1	estrogen receptor 1	-2.847	0.02280869
CAPS	CAPS	calcyphosine	-2.844	0.007795827
SLC47A1	SLC47A1	solute carrier family 47 (multidrug and toxin extrusion), member 1	-2.825	0.01975898
PLEKHG7	PLEKHG7	pleckstrin homology domain containing, family G (with RhoGef domain) member 7	-2.805	0.02538975
ADH6	ADH6	alcohol dehydrogenase 6 (class V)	-2.79	0.03845899
POU5F1B	POU5F1B	POU class 5 homeobox 1B	-2.788	0.005839523
C2orf62	CATIP	ciliogenesis associated TTC17 interacting protein	-2.783	0.01278143
C6orf97	CCDC170	coiled-coil domain containing 170	-2.775	0.01210502
STX19	STX19	syntaxin 19	-2.747	0.003759129
NXF3	NXF3	nuclear RNA export factor 3	-2.724	0.01604042
CXCL3	CXCL3	chemokine (C-X-C motif) ligand 3	-2.706	0.01426048
CCDC42B	CFAP73	cilia and flagella associated protein 73	-2.689	0.01651241
CCDC135	DRC7	dynein regulatory complex subunit 7	-2.668	0.03935181
CXCL2	CXCL2	chemokine (C-X-C motif) ligand 2	-2.667	0.01210502
TMEM211	TMEM211	transmembrane protein 211	-2.666	0.01330222
FAM166B	FAM166B	family with sequence similarity 166, member B	-2.663	0.04996141
CCDC108	CCDC108	coiled-coil domain containing 108	-2.657	0.02062955
TTLL2	TTLL2	tubulin tyrosine ligase-like family member 2	-2.609	0.009972617
RNF183	RNF183	ring finger protein 183	-2.606	0.009361028
TSIX	TSIX	TSIX transcript, XIST antisense RNA	-2.599	0.03047474
FUT6	FUT6	fucosyltransferase 6 (alpha (1,3) fucosyltransferase)	-2.589	0.02654826
C1orf194	C1orf194	chromosome 1 open reading frame 194	-2.585	0.04541916
ARSE	ARSE	arylsulfatase E (chondrodysplasia punctata 1)	-2.584	0.02941884
ACSL5	ACSL5	acyl-CoA synthetase long-chain family member 5	-2.58	0.01278143

ASB12	ASB12	ankyrin repeat and SOCS box containing 12	-2.572	0.005839523
ST6GALNAC1	ST6GALNAC1	ST6 (alpha-N-acetyl-neuraminyl-2,3-beta-galactosyl-1,3)-N-acetylgalactosaminide alpha-2,6-sialyltransferase 1	-2.57	0.0435928
POU5F1	POU5F1	POU class 5 homeobox 1	-2.565	0.01210502
CACNA1I	CACNA1I	calcium channel, voltage-dependent, T type, alpha 1I subunit	-2.55	0.03738103
SAA4	SAA4	serum amyloid A4, constitutive	-2.548	0.04917746
UBQLNL	UBQLNL	ubiquilin-like	-2.54	0.004403746
USH1C	USH1C	Usher syndrome 1C	-2.537	0.04756977
CALN1	CALN1	calneuron 1	-2.526	0.02538975
ZMYND12	ZMYND12	zinc finger, MYND-type containing 12	-2.524	0.005839523
FOXJ1	FOXJ1	forkhead box J1	-2.516	0.02538975
IL19	IL19	interleukin 19	-2.511	0.04944049
LRRC50	DNAAF1	dynein, axonemal, assembly factor 1	-2.507	0.02803746
CCDC17	CCDC17	coiled-coil domain containing 17	-2.493	0.008275055
CTSE	CTSE	cathepsin E	-2.487	0.02771723
GGTLC2	GGTLC2	gamma-glutamyltransferase light chain 2	-2.485	0.00882525
RP1	RP1	retinitis pigmentosa 1 (autosomal dominant)	-2.468	0.02654826
COL28A1	COL28A1	collagen, type XXVIII, alpha 1	-2.467	0.003449271
DEFB4A	DEFB4A/DEFB4B	defensin, beta 4A	-2.467	0.03303572
UBXN10	UBXN10	UBX domain protein 10	-2.452	0.02062955
TNFSF12-TNFSF13	TNFSF12-TNFSF13	TNFSF12-TNFSF13 readthrough	-2.432	0.01136266
TGM3	TGM3	transglutaminase 3	-2.428	0.03047474
TP53AIP1	TP53AIP1	tumor protein p53 regulated apoptosis inducing protein 1	-2.405	0.01330222
ACY3	ACY3	aminoacylase 3	-2.404	0.01330222
C19orf33	C19orf33	chromosome 19 open reading frame 33	-2.392	0.04996141
TMED6	TMED6	transmembrane p24 trafficking protein 6	-2.389	0.007247798
TMEM101	TMEM101	transmembrane protein 101	-2.381	0.01210502
400891	DLG4	discs, large homolog 4 (Drosophila)	-2.377	0.02396564
SPDEF	SPDEF	SAM pointed domain containing ETS transcription factor	-2.376	0.0435928
C19orf18	C19orf18	chromosome 19 open reading frame 18	-2.356	0.008275055
NME5	NME5	NME/NM23 family member 5	-2.354	0.01517195
GLYATL1	GLYATL1	glycine-N-acyltransferase-like 1	-2.323	0.03114439
FUT3	FUT3	fucosyltransferase 3 (galactoside 3(4)-L-fucosyltransferase, Lewis blood group)	-2.32	0.03403585
CNGA4	CNGA4	cyclic nucleotide gated channel alpha 4	-2.318	0.02631624
KLKB1	KLKB1	kallikrein B, plasma (Fletcher factor) 1	-2.31	0.02621325
TEKT3	TEKT3	tektin 3	-2.302	0.002204647
SRD5A2	SRD5A2	steroid-5-alpha-reductase, alpha polypeptide 2 (3-oxo-5 alpha-steroid delta 4-dehydrogenase alpha 2)	-2.301	0.02941884
AMY2A	AMY2A	amylase, alpha 2A (pancreatic)	-2.297	0.0110201
NOXA1	NOXA1	NADPH oxidase activator 1	-2.295	0.001248518
VWA3B	VWA3B	von Willebrand factor A domain containing 3B	-2.287	0.04258637
BCL2L15	BCL2L15	BCL2-like 15	-2.277	0.02396564
EYA2	EYA2	EYA transcriptional coactivator and phosphatase 2	-2.272	0.03403585
SIGLEC15	SIGLEC15	sialic acid binding Ig-like lectin 15	-2.261	0.03738103
C3	NAD+		-2.26	0.03240097
UNC93A	UNC93A	unc-93 homolog A (C. elegans)	-2.251	0.02206378
ASRGL1	ASRGL1	asparaginase like 1	-2.242	0.01210502
284276	LINC00908	long intergenic non-protein coding RNA 908	-2.238	0.01210502

STX18	STX18	syntaxin 18	-2.234	0.001608572
FGFR2	FGFR2	fibroblast growth factor receptor 2	-2.229	0.009972617
ABCA13	ABCA13	ATP-binding cassette, sub-family A (ABC1), member 13	-2.227	0.02396564
AMBP	AMBP	alpha-1-microglobulin/bikunin precursor	-2.198	0.04541916
AMY2B	AMY2B	amylase, alpha 2B (pancreatic)	-2.189	0.006249817
SPATA18	SPATA18	spermatogenesis associated 18	-2.189	0.01426048
HNF1A	HNF1A	HNF1 homeobox A	-2.188	0.01159343
CCDC129	CCDC129	coiled-coil domain containing 129	-2.188	0.04975449
DNAH6	DNAH6	dynein, axonemal, heavy chain 6	-2.181	0.02167748
DNAH9	DNAH9	dynein, axonemal, heavy chain 9	-2.163	0.04756977
SPATA17	SPATA17	spermatogenesis associated 17	-2.143	0.01517195
KIAA1324	KIAA1324	KIAA1324	-2.138	0.03047474
EMX2	EMX2	empty spiracles homeobox 2	-2.133	0.004001155
CASC1	CASC1	cancer susceptibility candidate 1	-2.124	0.03047474
GGTLC1	GGTLC1	gamma-glutamyltransferase light chain 1	-2.121	0.007247798
KISS1	KISS1	KiSS-1 metastasis-suppressor	-2.12	0.01874526
CUBN	CUBN	cubilin (intrinsic factor-cobalamin receptor)	-2.115	0.001248518
DLEC1	DLEC1	deleted in lung and esophageal cancer 1	-2.112	0.04541916
CFAP221	CFAP221	cilia and flagella associated protein 221	-2.108	0.04996141
PRSS33	PRSS33	protease, serine, 33	-2.107	0.02941884
LRRC46	LRRC46	leucine rich repeat containing 46	-2.104	0.02167748
TTC18	CFAP70	cilia and flagella associated protein 70	-2.092	0.01330222
APOL4	APOL4	apolipoprotein L, 4	-2.087	0.01159343
CRYBB1	CRYBB1	crystallin, beta B1	-2.084	0.03738103
OCA2	OCA2	oculocutaneous albinism II	-2.084	0.04684127
CFI	CFI	complement factor I	-2.079	0.01330222
TEKT4	TEKT4	tektin 4	-2.078	0.04756977
CPM	CPM	carboxypeptidase M	-2.076	0.02062955
KLK14	KLK14	kallikrein-related peptidase 14	-2.075	0.03738103
CYP3A5	CYP3A5	cytochrome P450, family 3, subfamily A, polypeptide 5	-2.075	0.04756977
TNNT2	TNNT2	troponin T type 2 (cardiac)	-2.06	0.01517195
LYPD1	LYPD1	LY6/PLAUR domain containing 1	-2.059	0.03935181
CD1A	CD1A	CD1a molecule	-2.052	0.01651241
SLC5A9	SLC5A9	solute carrier family 5 (sodium/sugar cotransporter), member 9	-2.041	0.008275055
AGBL2	AGBL2	ATP/GTP binding protein-like 2	-2.041	0.02538975
GGT1	GGT1	gamma-glutamyltransferase 1	-2.04	0.02654826
BCO2	BCO2	beta-carotene oxygenase 2	-2.037	0.006626255
NCRNA00116	LINC00116	long intergenic non-protein coding RNA 116	-2.037	0.03935181
LDLRAD2	LDLRAD2	low density lipoprotein receptor class A domain containing 2	-2.035	0.003219602
PCP2	PCP2	Purkinje cell protein 2	-2.031	0.009361028
C6orf41	LINC00240	long intergenic non-protein coding RNA 240	-2.027	0.01416221
C12orf27	HNF1A-AS1	HNF1A antisense RNA 1	-2.022	0.01604042
RSPH10B2	RSPH10B/RSPH10B2	radial spoke head 10 homolog B (Chlamydomonas)	-2.022	0.02167748
C10orf79	CFAP43	cilia and flagella associated protein 43	-2.021	0.02538975
C1orf88	PIFO	primary cilia formation	-2.02	0.04996141
TDGF1	TDGF1	teratocarcinoma-derived growth factor 1	-2.014	0.0435928
SEC16B	SEC16B	SEC16 homolog B, endoplasmic reticulum export factor	-2.004	0.004035443

RAP1GAP	RAP1GAP	RAP1 GTPase activating protein	-2.002	0.03547051
POSTN	POSTN	periostin, osteoblast specific factor	2.018	0.0110201
FAM21A	FAM21A/FAM21C	family with sequence similarity 21, member C	2.107	0.02062955
PCDHGA3	PCDHGA3	protocadherin gamma subfamily A, 3	2.128	0.01159343
PCDHGA2	PCDHGA2	protocadherin gamma subfamily A, 2	2.147	0.01075619
GAS1	GAS1	growth arrest-specific 1	2.162	0.01777309
CBS	CBS/CBSL	cystathionine-beta-synthase	2.166	0.02538975
SLC6A17	SLC6A17	solute carrier family 6 (neutral amino acid transporter), member 17	2.234	0.02179432
MED12L	MED12L	mediator complex subunit 12-like	2.288	0.009972617
PCOLCE2	PCOLCE2	procollagen C-endopeptidase enhancer 2	2.312	0.03454013
PTGER3	PTGER3	prostaglandin E receptor 3 (subtype EP3)	2.37	0.01651241
CACNA2D1	CACNA2D1	calcium channel, voltage-dependent, alpha 2/delta subunit 1	2.381	0.01598562
FOXD1	FOXD1	forkhead box D1	2.384	0.01962442
KCNH2	KCNH2	potassium channel, voltage gated eag related subfamily H, member 2	2.485	0.02803746
HOXA13	HOXA13	homeobox A13	2.523	0.01651241
PTGIS	PTGIS	prostaglandin I2 (prostacyclin) synthase	2.571	0.01777309
EIF3C	EIF3C	eukaryotic translation initiation factor 3, subunit C	2.588	0.0332791
PDLIM3	PDLIM3	PDZ and LIM domain 3	2.594	0.005299897
GPC3	GPC3	glypican 3	2.649	0.01159343
PCSK1	PCSK1	proprotein convertase subtilisin/kexin type 1	2.72	0.04530378
CHRDL1	CHRDL1	chordin-like 1	2.844	0.02894284
SIX2	SIX2	SIX homeobox 2	2.896	0.02538975
CHRDL2	CHRDL2	chordin-like 2	2.919	0.01210502
BIRC5	BIRC5	baculoviral IAP repeat containing 5	3.262	0.007247798

S12. Enriched Pathways in Training-1 Patients with Extreme MS7 Scores

Table S12 provides a summary of the top 25 enriched molecular pathways with significant differentially expressed genes in Training-1 patients with extreme MS7 classifier scores (upper quartile [Q4] vs. lower quartile [Q1]. A –log p-value ≥ 2.0). Z-score denotes putative pathway activation state 1. Decreased and elevated columns denote the number and percent of enriched genes decreased or elevated in the patients with a Q4 vs. Q1 MS7 score for a given pathway of interest. No overlap denotes the number and percent of canonical pathway genes not enriched in the primary dataset. Significant genes indicate specific data set enriched genes for each pathway.

Table S12. Enriched Pathways in Training-1 Patients with Extreme MS7 Scores

Canonical Pathways	-log (*P-value*)	z-score	Decreased	Elevated	No Overlap	Significant Genes
EIF2 Signaling	9.71	-3.024	28/187 (15%)	13/187 (7%)	146/187 (78%)	RPL11, RAF1, RPL22, PIK3CA, EIF3C, RPL39, RPLP2, PIK3R4, EIF4G1, EIF2S1, RPS4X, RPL27A, RPL13, EIF5, RPS9, RPL19, RPS2, GSK3B, RPS3, MAP2K1, RPL18, GRB2, RPL34, RPL17, RPS8, RPL29, AGO2, RPL23, RPL12, EIF2S2, RPL28, RPL9, RPS6, RPL15, RPS15, EIF4A3, RPL10, RPS25, RPL37, RPL6, RPL13A
Cell Cycle Control of Chromosomal Replication	5.84	NaN	0/27 (0%)	11/27 (41%)	16/27 (59%)	MCM3, MCM6, CDC45, MCM2, CDT1, CDK4, RPA1, MCM4, CDK2, MCM7, ORC1
Role of BRCA1 in DNA Damage Response	5.57	1.732	0/78 (0%)	19/78 (24%)	59/78 (76%)	ARID1A, TOPBP1, FANCG, BARD1, MDC1, RPA1, RFC1, PLK1, FANCE, RAD51, RFC4, FANCD2, MSH2, E2F1, MSH6, FAAP24, RFC2, BRIP1, HLTF
Mismatch Repair in Eukaryotes	4.72	NaN	0/18 (0%)	8/18 (44%)	10/18 (56%)	PCNA, MSH2, RFC4, MSH6, RFC2, FEN1, RFC1, RPA1
Hereditary Breast Cancer Signaling	4.55	NaN	2/131 (2%)	22/131 (17%)	107/131 (82%)	PIK3CA, ARID1A, POLR2D, GADD45B, HDAC8, FANCG, BARD1, CDK4, RPA1, RFC1, PIK3R4, RAD51, FANCE, PALB2, HDAC3, FANCD2, MSH2, RFC4, XPC, E2F1, MSH6, FAAP24, RFC2, HLTF
Role of CHK Proteins in Cell Cycle Checkpoint Control	3.86	0.816	0/55 (0%)	13/55 (24%)	42/55 (76%)	PCNA, RFC4, PPP2R2A, PPP2R5D, E2F1, RFC2, MDC1, CLSPN, PLK1, RFC1, RPA1, CDK2, CDC25A
Estrogen Receptor Signaling	3.80	NaN	7/128 (5%)	15/128 (12%)	106/128 (83%)	MED18, MED12L, RAF1, PRKDC, POLR2D, GRB2, MED1, TBP, TAF10, GTF2A1, G6PC3, MED14, PGR, HDAC3, CDK8, TAF4, MED15, TRRAP, ERCC3, GTF2H5, MAP2K1, ESR1
Estrogen-mediated S-phase Entry	3.67	1.414	1/24 (4%)	7/24 (29%)	16/24 (67%)	CCNA2, E2F1, CDK4, CDKN1B, ESR1, CDK2, SKP2, CDC25A
IL-17A Signaling in Airway Cells	3.58	1	8/66 (12%)	6/66 (9%)	52/66 (79%)	PIK3CA, CCL20, IKBKE, IL17RA, PIK3R4, IL19, CXCL3, MAPK14, TRAF3IP2, NFKBIA, MUC5B, GSK3B, DEFB4A/DEFB4B, MAP2K1

Mitotic Roles of Polo-Like Kinase	3.58	0	2/66 (3%)	12/66 (18%)	52/66 (79%)	KIF23, ESPL1, PPP2R2A, PPP2R5D, PRC1, CDC23, PLK1, SMC1A, ANAPC4, HSP90AB1, FBXO5, CDC16, RAD21, CDC25A
Regulation of eIF4 and p70S6K Signaling	3.20	0.707	9/150 (6%)	14/150 (9%)	127/150 (85%)	RAF1, EIF3C, PIK3CA, GRB2, PPP2R2A, PPP2R5D, RPS8, AGO2, PIK3R4, EIF4G1, EIF2S1, RPS4X, EIF2S2, RPS6, RPS15, MAPK14, EIF4EBP3, EIF4A3, RPS9, RPS25, RPS2, RPS3, MAP2K1
Protein Ubiquitination Pathway	2.84	NaN	12/259 (5%)	21/259 (8%)	226/259 (87%)	USP14, DNAJC17, HSPA14, PSMA7, PSMB10, CDC23, DNAJC10, USP39, DNAJB13, USP13, HSP90AB1, DNAJC4, USP40, DNAJC22, BIRC3, HSPA4L, DNAJB12, PSMD13, USP9X, HSPA9, USP19, USP1, PSMD3, PSMD8, SKP2, HSPA8, ANAPC4, PSMD11, HSCB, UBE2G1, PSMD2, PSMD1, VHL
Cyclins and Cell Cycle Regulation	2.81	0	1/78 (1%)	13/78 (17%)	64/78 (82%)	RAF1, HDAC8, PPP2R2A, PPP2R5D, CDK4, SKP2, CCNA2, HDAC3, E2F1, TGFB3, CDKN1B, GSK3B, CDK2, CDC25A
NRF2-mediated Oxidative Stress Response	2.79	2.309	10/180 (6%)	15/180 (8%)	155/180 (86%)	AKR7A2, RAF1, USP14, DNAJC17, PIK3CA, DNAJB12, PPIB, DNAJC10, JUNB, PIK3R4, DNAJB13, MAFG, GSR, FOS, MAPK14, MGST2, DNAJC4, STIP1, DNAJA3, GSK3B, FKBP5, MAP2K1, HACD3, MAP2K5, GSTK1
BER pathway	2.78	NaN	0/13 (0%)	5/13 (38%)	8/13 (62%)	PCNA, POLG, FEN1, OGG1, PARP1
Assembly of RNA Polymerase III Complex	2.78	NaN	1/13 (8%)	4/13 (31%)	8/13 (62%)	GTF3C4, TBP, SF3A1, BRF1, POLR3D
Adipogenesis pathway	2.68	NaN	8/135 (6%)	12/135 (9%)	115/135 (85%)	ATG7, KAT7, HDAC8, TNFRSF1A, HAT1, SAP30L, FGFR2, SAP130, SENP2, SAP30, BSCL2, FGF1, HDAC3, PAXIP1, KAT6A, AGPAT2, EZH2, ERCC3, GTF2H5, RBBP4
IL-17A Signaling in Fibroblasts	2.48	NaN	5/35 (14%)	3/35 (9%)	27/35 (77%)	FOS, MAPK14, TRAF3IP2, NFKBIA, IKBKE, GSK3B, NFKBIZ, IL17RA
p53 Signaling	2.28	-1.667	2/98 (2%)	13/98 (13%)	83/98 (85%)	PRKDC, TP53AIP1, PIK3CA, GADD45B, MED1, TOPBP1, CDK4, PIK3R4, BIRC5, PCNA, MAPK14, E2F1, GSK3B, CTNNB1, CDK2
Molecular Mechanisms of Cancer	2.18	NaN	12/373 (3%)	29/373 (8%)	332/373 (89%)	RAF1, GAB2, PIK3CA, PSENEN, ADCY4, GNA11, CDK4, BMPR1B, PIK3R4, NFKBIA, FANCD2, GNA15, PLCB1, GSK3B, CTNNB1, MAP2K1, BIRC3, CDC25A, PRKDC, STK36, GRB2, HAT1, ARHGEF15, ADCY3, PRKAR2A, AURKA, RAC3, FOS, DAXX, MAPK14, RHOA, E2F1, TGFB3, PLCB3, IHH, CDKN1B, ELK1, GNAL, CDK2, CAMK2G, PSEN1
Cell Cycle: G1/S Checkpoint Regulation	2.18	-1	2/64 (3%)	9/64 (14%)	53/64 (83%)	RPL11, HDAC3, HDAC8, E2F1, CDK4, TGFB3, CDKN1B, GSK3B, CDK2, SKP2, CDC25A
Acute Phase Response Signaling	2.13	0.258	16/171 (9%)	6/171 (4%)	149/171 (87%)	RAF1, SERPING1, PIK3CA, HPX, ITIH3, TNFRSF1A, GRB2, AMBP, IKBKE, SAA2, SAA4, HNF1A, FOS, KLKB1, IL18, HP, MAPK14, NFKBIA, C4BPA, SAA1, ELK1, MAP2K1
Chronic Myeloid Leukemia Signaling	2.10	NaN	2/93 (2%)	12/93 (13%)	79/93 (85%)	RAF1, GAB2, PIK3CA, HDAC3, HDAC8, GRB2, CRKL, E2F1, TGFB3, CDK4, IKBKE, CDKN1B, PIK3R4, MAP2K1
Aldosterone Signaling in Epithelial Cells	2.09	1.667	9/162 (6%)	12/162 (7%)	141/162 (87%)	RAF1, DNAJC17, PIK3CA, DNAJB12, HSPA14, PDIA3, HSPA9, DNAJC10, PIK3R4, DNAJB13, HSPA8, HSP90AB1, DNAJC4, HSCB, PLCB3, PLCB1, DNAJC22, MAP2K1, PIP4K2C, PI4KA, HSPA4L
PPARα/RXRα Activation	2.08	-1.265	8/183 (4%)	15/183 (8%)	160/183 (87%)	RAF1, PRKAB1, GRB2, MED1, PDIA3, CPT1B, ADCY4, ADCY3, GNA11, PRKAR2A, NCOA6, IKBKE, TGS1, CAND1, MAPK14, NFKBIA, HSP90AB1, GNA15, FASN, TGFB3, PLCB3, PLCB1, MAP2K1

S13. Pathway Analyses of Differentially Expressed Transcripts in Endometrioid Endometrial Cancer Patients Classified with High versus Low Risk of Metastasis.

In Figure 4, data reflects the percent contribution of transcripts to enriched canonical pathways that are elevated (highlighted red) or decreased (highlighted green) in high versus low risk patients. Predicted activation states of canonical pathways are based on abundance trends of associated transcripts relative to the putative functional role of associated candidates in the pathway of interest. Pathways predicted to be activated (highlighted orange, z-score $\geq$ 1.0) or inhibited (highlighted blue, z-score $\leq$ -1.0) are further denoted. Canonical pathways are ranked by most (EIF2 Signaling) to least (PPARα/ RXRα Activation) significantly enriched. Pathway analyses was performed using Ingenuity Pathway Analysis software (Qiagen). Although an inverse relationship was observed between MS7 score and ESR1 transcript expression, functional pathway analysis suggests that estrogen signaling pathways continue to function as potential drivers in patients with metastatic disease, possibly by ligand-dependent and independent signaling mechanisms, such as via AKT-dependent regulation of ESR1. Pathways predicted to be activated in Q4 patients included regulation of DNA damage response (DDR) and steroid hormone signaling, i.e. aldosterone and estrogen-mediated S-phase entry signaling. BRCA1-associated DDR signaling was further predicted to be activated in Q4 patient tissues suggesting that patients with metastatic disease have increased homologous recombination DNA repair activities as this DDR mechanism is commonly associated with BRCA1 function. Enrichment of DNA replication and orthogonal DNA repair pathways corresponded with elevations of the cell proliferation marker MKI67 in the metastatic patients (0.982, Log2 fold-change, q-value = 0.03). Pathways predicted to be inhibited in Q4 patient tissues include p53 signaling as well as G1/S checkpoint regulation. TBRG1, a member of the MS7 risk score, has been shown to promote TP53 activation and may

lead to impaired TP53 signaling in metastatic patients. Table S8 highlights the differentially expressed genes in Q4 vs. Q1 patients with the most extreme fold changes.

REFERENCES for SUPPLEMENT S13.

- Kramer A, Green J, Pollard J Jr, Tugendreich S. Causal analysis approaches in Ingenuity Pathway Analysis. Bioinformatics 2014; 30 (4):523-30.
- Vilgelm A, Lian Z, Wang H, Beauparlant SL, Klein-Szanto A, Ellenson LH, Di Cristofano A. (2006) Akt-mediated phosphorylation and activation of estrogen receptor alpha is required for endometrial neoplastic transformation in Pten+/- mice. Cancer Res 66, 3375-3380.
- Shah YM, Rowan BG. The Src kinase pathway promotes tamoxifen agonist action in Ishikawa endometrial cells through phosphorylation-dependent stabilization of estrogen receptor (alpha) promoter interaction and elevated steroid receptor coactivator 1 activity. Mol Endocrinol. 2005;19: 732-748.
- Powell SN, Kachnic LA. Roles of BRCA1 and BRCA2 in homologous recombination, DNA replication fidelity and the cellular response to ionizing radiation. Oncogene 2003;22(37):5784-91.
- Tompkins VS, et al. A novel nuclear interactor of ARF and MDM2 (NIAM) that maintains chromosomal stability. J Biol Chem. 2007;282(2):1322-33.
- Reed SM, et al. Nuclear interactor of ARF and Mdm2 regulates multiple pathways to activate p53. Cell Cycle 2014;13(8):1288-98.

S14. Stage Distribution of RNA Sequencing-Based MS7 Score

Figure S14 illustrates the stage distribution of MS7 Scores in endometrioid endometrial cancer patients from the UCEC TCGA Research Network.

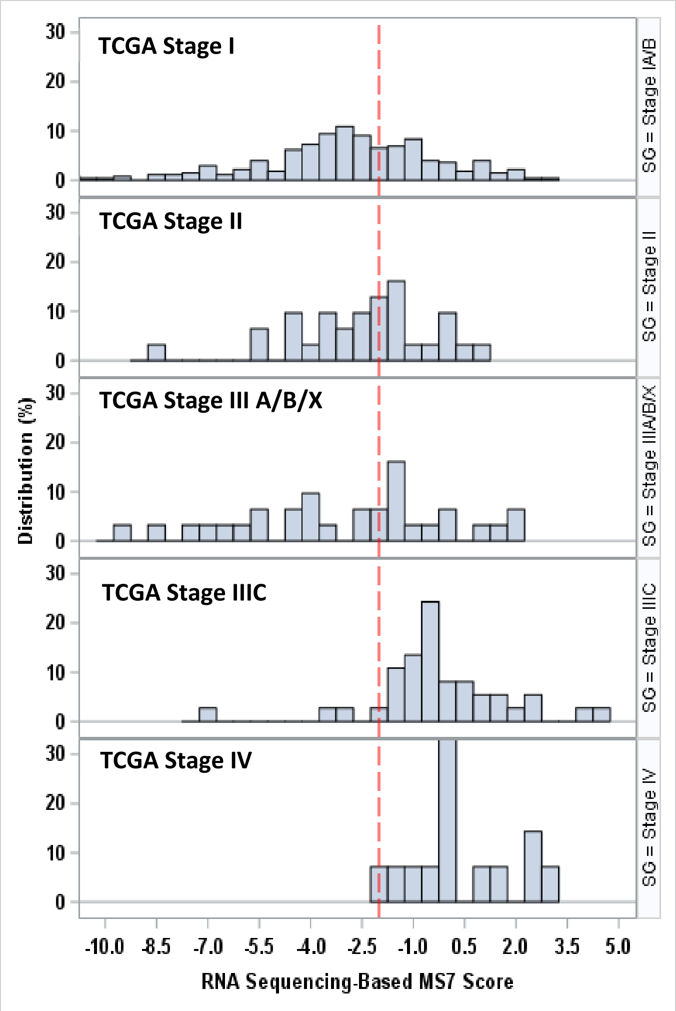

Figure S14. The distribution of 389 endometrioid endometrial cancer (EEC) patients from the Cancer Genome Atlas (TCGA) Uterine Corpus Endometrial Carcinoma (UCEC) Research Network by stage and MS7 score with the optimal cut-point indicated using a red dashed line.

Supplement S1 to S15

S15. Additional Characteristics of the Cohorts and Exploratory Evaluations of the Potential Prognostic Relevance of the MS7 Classifier

This section starts with additional characteristics for the Training and Validation Cohorts (**Table S15.1**). The relationship between high relative low RNA sequencing based MS7 score and measures of clinical outcome including progression-free survival and overall survival are displayed in **Table S15.2** using unadjusted, stage adjusted or fully adjusted Cox analyses. The fully adjusted Cox analyses included corrections for age, stage and tumor grade. Additional analyses of progression-free survival were performing using the MS7 classifier and clinical characteristics with adjuvant treatment (S15.3) or without adjuvant treatment (**Table S15.4**). **Table S15.5** compares the prediction of disease progression using the clinical variables compared with the clinical variables and the MS7 classifier. **Table S15.6** shows the relationship between different biomarkers including the MS7 classier and progression-free survival in all the endometrioid endometrial cancer patients from the Cancer Genome Atlas (TCGA) Research Network in the Uterine Corpus Endometrial Carcinoma (UCEC) project.

Supplement S15.1 Treatment and outcome characteristics for the endometrioid endometrial cancer patients with stage I, IIIC or IV disease and RNA sequencing data or Affymetrix Plus 2.0 microarray data.

	**RNA Sequencing Data**		**Affymetrix Microarray Data**	
	Training-1	***Validation-1***	***Training-2***	***Validation-2***
Treatment				
No Adjuvant Therapy	28 (37.3)	134 (58.3)	-	-
Endocrine Therapy	1 (1.3)	3 (1.3)	-	-
Radiotherapy (RT)	9 (12.0)	64 (27.8)	-	-
Chemotherapy	14 (18.7)	6 (2.6)	-	-
Chemotherapy and RT	23 (30.7)	23 (10.0)	-	-
Unknown	-	*15*	*64*	*81*
Progression within 60 months				
No Progression	*37 (59.7)*	*179 (82.9)*	*51 (79.7)*	-
Progression	*25 (40.3)*	*37 (17.1)*	*13 (20.3)*	-
Not Evaluable or Missing	*13*	*14*		*81*
Survival within 60 months				
Alive	*60 (80.0)*	*212 (92.2)*	*48 (75.0)*	-
Dead	*15 (20.0)*	*18 (7.80*	*16 (25.0)*	-
Unknown	-	*15*	-	*81*

Criteria for the stage I cases in the training compared with the validation cohorts was strict for the former and loose for the later (see Methods for details). Interquartile range [IQR]. Percent provided in parentheses.

Table S15.2. Relationship between High relative to Low MS7 Score and Clinical Outcomes including Progression-Free Survival and Overall Survival in Women with Stage I-IV Endometrioid Endometrial Cancer from the Cancer Genome Atlas (TCGA) Research Network Uterine Corpus Endometrial Carcinoma (UCEC) Project

Type of Clinical Outcome	Type of Cox Model	Hazard Ratio (95% Confidence Interval)
Progression-Free Survival	Unadjusted	2.51 (1.59 – 3.95)
	Stage Adjusted	1.87 (1.13 – 3.07)
	Fully Adjusted	1.89 (1.05 – 3.42)
Overall Survival	Unadjusted	2.48 (1.35 – 4.56)
	Stage Adjusted	1.63 (0.83 – 3.21)
	Fully Adjusted	1.10 (0.49 – 2.46)

Stage classified as: Stage I vs. Stage II vs. Stage III vs. Stage IV. Tumor grade (G) classified as: G1 vs. G2 vs. G3. Myometrial Invasion (MI) classified as 0-50% vs. ≥50%. Unadjusted hazard ratio and 95% confidence interval estimated from Cox model without control for other variables. Stage-adjusted hazard ratio and 95% confidence interval estimated from Cox model with control for stage. Fully adjusted hazard ratio and 95% confidence interval estimated from Cox model with control for age (continuous variable), stage, tumor grade and MI.

Table S15.3 Association between Clinical Variables and MS7 with Progression-free Survival (PFS) in Endometrioid Endometrial Cases from the Cancer Genome Atlas (TCGA) Provided Adjuvant Treatment.

	Univariate Analysis		Multivariate Analysis	
	HR (95% C.I.)	*P* value	HR (95% C.I.)	*P* value
Age (years)				
≥65 vs. <65	1.17 (0.75 – 1.83)	0.479	1.40 (0.84 – 2.31)	0.195
Stage				
Advanced vs. Early	2.91 (1.86 – 4.54)	<0.0001	2.11 (1.25 – 3.59)	0.006
Tumor Grade				
High vs. Low	1.76 (1.12 – 2.75)	0.014	0.95 (0.55 – 1.64)	0.857
MI				
≥50% vs. <50%	1.90 (1.17 – 3.06)	0.009	1.42 (0.87 – 2.34)	0.164
Adjuvant Treatment				
Yes vs. No	2.77 (1.67 – 4.60)	<0.0001	1.80 (0.97 – 3.33)	0.062
MS7				
High vs. Low	2.53 (1.60 – 3.99)	<0.0001	1.93 (1.08 – 3.47)	0.027

Table S15.4 Association between Clinical Variables and MS7 with Progression-free Survival (PFS) in Endometrioid Endometrial Cases from the Cancer Genome Atlas (TCGA) Not Given Adjuvant Treatment.

	Univariate Analysis		Multivariate Analysis	
	HR (95% C.I.)	*P* value	HR (95% C.I.)	*P* value
Age (years)				
≥65 vs. <65	1.17 (0.75 – 1.83)	0.479	1.29 (0.78 – 2.12)	0.317
Stage				
Advanced vs. Early	2.91 (1.86 – 4.54)	<0.0001	2.50 (1.49 – 4.17)	0.005
Tumor Grade				
High vs. Low	1.76 (1.12 – 2.75)	0.014	1.00 (0.58 – 1.71)	0.989
MI				
≥50% vs. <50%	1.90 (1.17 – 3.06)	0.009	1.56 (0.85 – 2.55)	0.076
MS7				
High vs. Low	2.53 (1.60 – 3.99)	<0.0001	2.03 (1.13 – 3.65)	0.019

Table S15.5 Prediction of Disease Progression based on Clinical Variables Compared to Clinical with the MS7 Classifier

	Predictors	Likelihood Ratio $\chi 2$	CPE
Model 1	clinical variables (age + stage + tumor grade + MI)	27.854	0.647
Model 2	clinical variables (age + stage + tumor Grade + MI) + MS7	33.486	0.667

Difference in likelihood ratio $\chi 2$ between two models: $\Delta\chi 2 = 5.632$, p=0.018.

Table S15.6 Association between ARID1A, CTNNB1, MKI67, ESR1 and MS7 with Progression-free Survival (PFS) in the Stage I-IV Endometrioid Endometrial Cases from the Cancer Genome Atlas (TCGA) including those who did and did not receive adjuvant treatment.

	Univariate Analysis		Multivariate Analysis	
	HR (95% C.I.)	*P* value	HR (95% C.I.)	*P* value
ARID1A	0.96 (0.78 – 1.20)	0.729	0.86 (0.62 – 1.19)	0.370
CTNNB1	0.99 (0.80 – 1.22)	0.892	0.95 (0.74 – 1.20)	0.652
MKI67	1.27 (1.01 – 1.59)	0.043	1.10 (0.86 – 1.41)	0.444
ESR1	1.36 (1.13 – 1.65)	0.001	1.20 (0.97 – 1.49)	0.086
MS7	1.51 (1.18 – 1.94)	0.001	1.39 (1.03 – 1.87)	0.029

HR estimated for increasing one standard deviation (SD) of ARID1A, CTNNB1, MKI67, MS7, or decreasing one SD of ESR1.
